# PhyloAcc-GT: A Bayesian Method for Inferring Patterns of Substitution Rate Shifts on Targeted Lineages Accounting for Gene Tree Discordance

**DOI:** 10.1093/molbev/msad195

**Published:** 2023-09-04

**Authors:** Han Yan, Zhirui Hu, Gregg W C Thomas, Scott V Edwards, Timothy B Sackton, Jun S Liu

**Affiliations:** Department of Statistics, Harvard University, Cambridge, MA, USA; Department of Statistics, Harvard University, Cambridge, MA, USA; Gladstone Institute of Data Science and Biotechnology, San Francisco, CA, USA; Informatics Group, Harvard University, Cambridge, MA, USA; Department of Organismic and Evolutionary Biology, Harvard University, Cambridge, MA, USA; Informatics Group, Harvard University, Cambridge, MA, USA; Department of Statistics, Harvard University, Cambridge, MA, USA

**Keywords:** molecular evolution, Bayesian phylogenetics, phylogenetic discordance

## Abstract

An important goal of evolutionary genomics is to identify genomic regions whose substitution rates differ among lineages. For example, genomic regions experiencing accelerated molecular evolution in some lineages may provide insight into links between genotype and phenotype. Several comparative genomics methods have been developed to identify genomic accelerations between species, including a Bayesian method called PhyloAcc, which models shifts in substitution rate in multiple target lineages on a phylogeny. However, few methods consider the possibility of discordance between the trees of individual loci and the species tree due to incomplete lineage sorting, which might cause false positives. Here, we present PhyloAcc-GT, which extends PhyloAcc by modeling gene tree heterogeneity. Given a species tree, we adopt the multispecies coalescent model as the prior distribution of gene trees, use Markov chain Monte Carlo (MCMC) for inference, and design novel MCMC moves to sample gene trees efficiently. Through extensive simulations, we show that PhyloAcc-GT outperforms PhyloAcc and other methods in identifying target lineage-specific accelerations and detecting complex patterns of rate shifts, and is robust to specification of population size parameters. PhyloAcc-GT is usually more conservative than PhyloAcc in calling convergent rate shifts because it identifies more accelerations on ancestral than on terminal branches. We apply PhyloAcc-GT to two examples of convergent evolution: flightlessness in ratites and marine mammal adaptations, and show that PhyloAcc-GT is a robust tool to identify shifts in substitution rate associated with specific target lineages while accounting for incomplete lineage sorting.

## Introduction

The ongoing deluge of whole-genome sequences across the tree of life, combined with new phylogenetic methods, have provided comparative biologists with powerful opportunities for a detailed understanding of variation in substitution rates among genes and lineages, with the aim of identifying regions of the genome evolving by natural selection and potentially linked to phenotypic evolution. Regions of the genome that are conserved between species are generally considered to be functional, with purifying selection resulting in lower substitution rates than expected under conditions of neutrality (Cooper, Stone, Asimenos, Program, et al. 2005). For example, in protein-coding genes, the rate of synonymous substitution is generally much higher than the rate of nonsynonymous substitution because nonsynonymous changes are more likely to be deleterious and removed by selection. In contrast, regions of the genome exhibiting accelerated substitution rates may have undergone positive directional selection or relaxation of purifying selection. Identifying these regions in a phylogenetic framework can therefore provide insight into the selective pressures acting on them and may enable the identification of potential changes in function in lineages of interest (Sackton et al. 2019; Kowalczyk et al. 2020; Espindola-Hernandez et al. 2022; Pollard et al. 2006).

A number of sophisticated methods exist to model how substitution rates in protein-coding genes vary across codons and lineages, such as PAML (Yang 1997b) branch-site models (Zhang et al. 2005), and models implemented in HyPhy (Pond and Muse 2005) including aBSREL (Smith et al. 2015) and BUSTED (Murrell et al. 2015). These models have been modified to account for multinucleotide mutations (Venkat et al. 2018; Lucaci et al. 2021), and some have been implemented to estimate changes in selective constraint (e.g., RELAX Wertheim et al. 2015). However, protein-coding genes are only a small fraction of the sequence that is conserved between species. Although comparative studies frequently estimate that 3–8% of vertebrate genomes are conserved, a significant majority of these regions are noncoding (Siepel et al. 2005; Consortium 2020). A number of popular methods exist to estimate simple models of variable conservation and acceleration across the genome (e.g., PHAST: Siepel et al. 2005; Hubisz et al. 2011, phyloP: Pollard et al. 2010, GERP: Cooper, Stone, Asimenos, Green, et al. 2005), but these approaches have largely focused on finding regions of conservation amongst the vast quantity of unconstrained sequence in the genome. Of these methods, phyloP (Pollard et al. 2010) from the PHAST (Hubisz et al. 2011) package conducts likelihood ratio tests to identify conservation in specific loci, as well as acceleration on prespecified lineages, modeling substitution rates on the target lineages using a scaling factor relative to the background rate. The BEAST package (Drummond and Suchard 2010) assumes a random local clock model, using an indicator variable to denote rate changes in each node and a Possion prior to control the total number of rate changes on the tree.

Other methods exist that jointly model substitution rates and phenotypic traits, one approach of the general effort to link genomic and phenotypic variation via phylogenetic trees (PhyloG2P; Smith et al. 2020). CoEvol (Lartillot and Poujol 2011) jointly models genomic substitution rates or presence/absence of genomic loci and continuous phenotypic traits using a multivariate Brownian diffusion process, or which identify deletions of loci associated with specific target lineages. In the “Forward Genomics” framework (Hiller et al. 2012; Prudent et al. 2016), genome sequences are imputed in ancestral species and compared among species groups with and without the trait of interest to identify associations between presence–absence of genomic loci and phenotypic variation. O’Connor and Mundy (2009, 2013) use the likelihood ratio test to detect associations between genotypes and a discrete phenotype. Under the null model (genotype and phenotype are independent), the rate matrices of the genotype and phenotype are independent, while a scaling factor depending on the phenotype is multiplied to the rate matrix of the genotype under the alternative model. TraitRate (Mayrose and Otto 2011; Levy Karin et al. 2017) also use likelihood methods to detect molecular rate changes associated with discrete phenotypes. Kowalczyk et al. (2019) developed RERconverge to estimate lineage-specific substitution rates on a phylogeny and demonstrated its use in linking substitution rates and mammalian lifespan (Kowalczyk et al. 2020). However, many of these methods lack complexity compared to their counterparts designed for protein-coding regions, which limit their ability to detect complex patterns of rate shifts, particularly when the species of interest do not form a monophyletic clade. There is thus a need for flexible methods that allow researchers to ask whether noncoding regions of the genome are accelerated specifically on branches of interest that may be associated with a trait or trait value of interest.

Recently, we developed PhyloAcc (Hu et al. 2019) (pronounced “Phylo-A-see-see”), a Bayesian method to quantify multiple shifts in substitution rate on a phylogeny. It infers the most probable pattern of shifts in substitution rate from sequence alignments and identifies loci with lineage-specific accelerations using Bayes factors, with many possible applications. For example, PhyloAcc and RERconverge have both been applied to test for correlations between convergent phenotypic states in a phylogeny and substitution rates (Chikina et al. 2016; Partha et al. 2017; Hu et al. 2019; Sackton et al. 2019; Tong et al. 2022). Whereas RERconverge is designed to test one pattern of rate shifts at a time on the tree, PhyloAcc can fit an unrestrained, full model to the input sequences, with rates and rate shifts estimated for each locus on each branch of the tree. Such a model allows researchers to ask general questions about genome-wide rate shifts, making possible tests for general patterns of evolution (e.g., “Which loci are accelerated on a prespecified branch or set of branches?”; “Which branches have an excess of rate shifts across all loci?”).

Although the methods mentioned above all estimate substitution rates along a phylogeny in different ways to assess shifts in evolutionary rates, they all accept as input a single species tree, and tacitly assume that the gene tree toplogies for all regions of the genome are identical to each other and to the species tree. However, phylogenies for different regions of the genome (which we refer to as gene trees by convention, even for nongenic regions of the genome) can differ from the species history and from other genomic regions due to multiple biological processes such as incomplete lineage sorting (ILS) or deep coalescence, which occurs when variation in ancestral species persisted after speciation, as well as introgression, and gene duplication and loss (Maddison 1997; Avise and Robinson 2008; Edwards 2009). Phylogenetic discordance is commonly observed across the tree of life (Jarvis et al. 2014; Pease et al. 2016; Lopes et al. 2021; Sun et al. 2021) and failure to account for it can lead to mis-estimation of substitution rates when sequences from discordant loci are mapped onto the species tree (Mendes and Hahn 2016) as well as incorrect inference of divergence times (Jennings and Edwards 2005; Angelis and Dos Reis 2015). Hahn and Nakhleh (2016) address the importance of considering gene tree topology variation when attempting to correlate substitution rates and phenotypic traits, specifically in the context of convergent evolution. Additionally, even when the gene tree and species tree are topologically identical, the two can still differ in their branch lengths (Edwards 2009).

Recently, the multispecies coalescent ILS-aware software Bayesian Phylogeography and Phylogenetics (BPP) was extended to include relaxed molecular clocks (Rannala and Yang 2017; Flouri et al. 2022). However, this model estimates overall rates of each branch of the species tree, as opposed to estimating rates of individual loci along each branch of the species tree. Ogilvie et al. (2017) improved the relaxed random clock model by considering the multispecies coalescent for more accurate inference of per-species substitution rates, while still assuming a common rate across loci per branch. Earlier works also exist for estimating a per branch evolutionary rate, while not accounting for ILS (Thorne et al. 1998; Kishino et al. 2001). In general, macroevolutionary models of molecular clocks and substitution rates have yet to embrace the widespread heterogeneity in gene trees found across the Tree of Life, with unknown consequences for molecular dating, PhyloG2P, and other questions in evolutionary biology (Bravo et al. 2019).

To more accurately estimate substitution rates and identify noncoding sequences that may have experienced accelerated evolution on particular lineages of a tree, here we extend the Bayesian model implemented in PhyloAcc to account for phylogenetic (henceforth “gene tree”) discordance. In our new model, named PhyloAcc-GT, we specify a prior distribution for the gene tree of each locus according to the multispecies coalescent model (Rannala and Yang 2003; Rannala et al. 2020). The full likelihood of the observed sequences from extant species and unobserved sequences from extinct species is defined conditioning on the latent gene tree estimated based on DNA substitution models. To sample gene trees from the posterior distribution, we also develop a Markov chain Monte Carlo (MCMC) algorithm (Liu 2008) using a new Metropolis–Hastings (MH) proposal distribution targeting the conditional posterior distribution of the gene tree conditioning on the species tree, sequence alignment and other parameters. We use subtree pruning and re-grafting when proposing new gene tree topologies, but carefully select candidate locations when re-grafting the tree to improve sampling efficiency. Through extensive simulations with various acceleration scenarios, we show that PhyloAcc-GT outperforms both PhyloAcc and *BEAST2 (Heled and Drummond 2009; Ogilvie et al. 2017), another Bayesian method for detecting substitution rate variation while accounting for ILS. We use PhyloAcc-GT to re-analyze two datasets, one consisting of 43 bird species with a focus on convergent loss of flight in ratites (Hu et al. 2019; Sackton et al. 2019) and the other consisting of 62 mammal species with a focus on convergent evolution of traits linked to marine life (Hu et al. 2019). We show that, after accounting for gene tree discordance PhyloAcc-GT is able to distinguish spurious signals of acceleration due to gene tree variation from true rate shifts. Finally, we also greatly improved the usability and efficiency of our software by developing a command-line user interface that facilitates preprocessing and postprocessing analyses and provides adaptive method selection (PhyloAcc vs. PhyloAcc-GT) based on site concordance factors (Ané et al. 2007; Minh, Hahn, et al. 2020) in the input alignments.

## Methods

### Bayesian Model to Estimate Substitution Rates in the Presence of Gene Tree Discordance

For a given sequence alignment of a locus, we estimate substitution rates in the presence of gene tree discordance based on an input species tree, hereafter denoted as T, and population size parameter $\theta \equiv 4N_{e}\mu$, where $N_{e}$ is the effective population size and μ is the mutation rate per site per generation. Parameter θ, whose estimation will be discussed next in Estimating Population Size Parameters section, measures the rate of coalescence in a species and is required when applying the multispecies coalescent model. T is a rooted bifurcating tree having *N* nodes, *S* extant species, and its branch lengths represent the expected number of neutral substitutions per site. Let $\Theta =(\theta_{1},\ldots ,\theta_{N})$ represent population sizes for the *N* species on the species tree. A set of target lineages in the phylogeny to test for acceleration can also be provided if known a priori.

To model patterns of shifts in substitution rate, PhyloAcc-GT follows the original PhyloAcc model and assumes that substitution rates can only take three values corresponding to three conservation states. The original PhyloAcc model uses three states to closely follow the modeling framework of phyloP (Pollard et al. 2010), which defined conserved, neutral, and accelerated states for individual loci. We use $Z=(Z_{1},\ldots ,Z_{N})\in \{0,1,2{\}}^{N}$ to represent these latent conservation states for the *N* species on the tree, where $Z_{s}=0$ indicates the background state with the background rate $r_{0}=1$, and $Z_{s}=1,2$ represent the conserved and the accelerated states, respectively, with the corresponding conserved rate $r_{1}<1$, and accelerated rate $r_{2}>r_{1}$. In this way, we frame our test for accelerated substitution rates relative to a premeasured background or neutral rate of substitution across the genome. Rates are inferred for up to three models: a null model that restricts all lineages in T to the background $r_{0}$ or conserved rate $r_{1}$, a restricted model in which the target lineages, if present, are allowed to evolve at $r_{2}$, and a full model in which all lineages can have any of the three *r* values.

We assume that the transition between states is Markovian with a prior transition probability matrix $\Phi =\left(\begin{array}{ccc} 1-\alpha & \alpha & 0\\ 0 & 1-\beta & \beta \\ 0 & 0 & 1 \end{array}\right)$. Here, α is the prior probability of a locus becoming conserved from the background state in a lineage, and β is the prior probability of losing conservation. We put uniform priors on the hyperparameters α and β. Substitution rates $r_{1}$ and $r_{2}$ follow gamma distributions a priori.

The genealogical relationships and branch lengths among sequences of a locus are modeled by a latent gene tree variable, denoted by G. The prior distribution of a gene tree given the species tree and population sizes is defined according to the standard multispecies coalescent model (Rannala and Yang 2003), which we briefly review here. For each species, we record the coalescence events backwards in time until speciation. Suppose for an ancestral species *s* with branch length $t_{s}$, there are $m_{s}$ sequences entering *s* at time 0, and $n_{s}$ leaving at time $t_{s}$, with $n_{s}<m_{s}$. Let $\tau_{m}^{s},\tau_{m-1}^{s},\ldots ,\tau_{n+1}^{s}$ be the coalescent times for the time ordered $(m-n{)}^{\mathrm{th}}$ coalescent events, and $\tau_{n}^{s}=t_{s}-\sum_{k=n_{s}+1}^{m_{s}}\tau_{k}^{s}$ be the remaining time from the last coalescent event to the next speciation event. The prior density of a gene tree G is


$$\begin{array}{rl} & f(G|T,\Theta )=\prod_{s=S+1}^{N}\{\prod_{k=n_{s}+1}^{m_{s}}\frac{2}{\theta_{s}}\exp\;\left(-\frac{k(k-1)}{\theta_{s}}\tau_{k}^{s}\right)\\ & \;\;\;\;\cdot \exp\;\left[-\frac{n_{s}(n_{s}-1)}{\theta_{s}}\left(t_{s}-\sum_{k=n_{s}+1}^{m_{s}}\tau_{k}^{s}\right)\right]\}\\ & \;\;\;\;\cdot \prod_{k=2}^{m_{N}}\frac{2}{\theta_{N}}\exp\;\left(-\frac{k(k-1)}{\theta_{N}}\tau_{k}^{N}\right) \end{array}$$

**Fig. 1.**
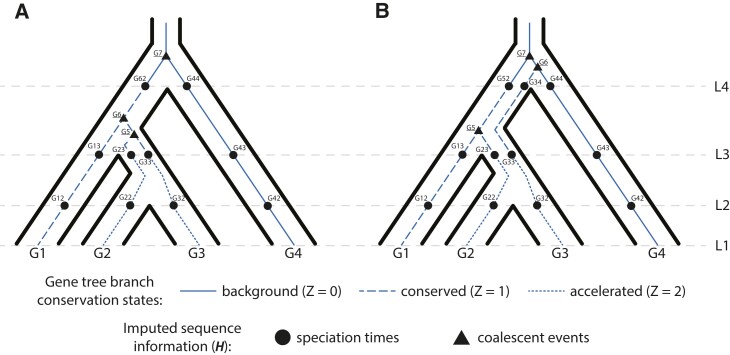
Conservation states and DNA evolution given a species tree and two gene trees. For each panel, the species tree is represented with the bold lines and encompasses a gene tree with thinner solid, dashed, or dotted lines. (A) A gene tree with a topology identical to the species tree, but with different branch lengths (coalescent times). (B) A gene tree that has a topology that is discordant with the species tree. For both panels, there are S = 4 extant species in the tree. L1 represents the current time and L2-L4 represent speciation times. A gene tree branch can span speciation times and can therefore be in different conservation states at different times. For both gene trees, gene sequences (G1 to G4) are observed and stored as ***Y***. Labeled points on the gene tree represent sequences imputed by PhyloAcc-GT. Triangular shaped points (G5, G6, and G7, underlined) represent gene sequence information at coalescent events prior to the speciation of those lineages. Circular points represent sequence information imputed at speciation times of any two lineages (L2, L3, or L4). Sequences are imputed at speciation times for every species in the locus, not just the two that are speciating. Imputed sequence information at both circular points and triangular points are stored in ***H***. For example, in panel A, ***H***^6^ = (***H***(6,1), ***H***(6,2), …, ***H***(6,5) = G13, G23, G33, G5, G6) stores the three sequences, G13, G23, and G33, at speciation time L3 (m_6_ = 3), two sequences G5 (coalescence of G2 and G3) and G6 (coalescence of G1 and G5) between speciation times L3 and L4, and one sequence, G62, at speciation time L4 (n_6_ = 1). The coalescence times τ^6^_3_, τ^6^_2_, and τ^6^_1_ correspond to the branch lengths from G23 to G5, G5 to G6, and G6 and G62, respectively.

Note that we model DNA sequences evolving according to a continuous-time Markov process defined on the gene tree, whereas the substitution rates are determined by the conservation states in each branch of the species tree. See figure 1 for an illustration.

Under the GTR substitution model, substitutions on one branch of the gene tree follow a continuous-time Markov process with the stationary distribution π and a rate matrix ***Q***. Instead of assuming a fixed and known stationary distribution of the base frequencies, $\pi =(\pi_{A},\pi_{C},\pi_{G},\pi_{T})$, for all loci as in the original PhyloAcc, in PhyloAcc-GT we model the stationary distribution of each locus independently. Here, we use the strand-symmetry model (Bielawski and Gold 2002; Singh et al. 2009) and assume that substitution rates are the same on the two DNA strands, that is, $\pi_{A}=\pi_{T}$ and $\pi_{G}=\pi_{C}$. Thus, we have only one free parameter $\pi_{A}$, for which we impose a half-Beta prior: $2\pi_{A}∼\text{Beta}(\gamma ,\gamma )$. The strand-symmetry assumption can be relaxed, in which case the Beta prior can be replaced by a Dirichlet distribution that can model a vector of probabilities of any finite dimensions.

For one locus of length *l*, let $Y=(Y_{\;j,s}{)}_{\;j=1\;:\;l}^{s=1\;:\;S}$ denote the observed aligned sequences in the *S* extant species. We use X={Y,H} to represent the complete data, where H stands for the unobserved sequences in ancestral species at both coalescent events on the gene tree and speciation events on the species tree.

Given all parameters and latent variables, the complete likelihood function is


(1) $$\begin{array}{rl} & P(X|Z,r,G,\Phi ,T,\Theta ,Q,\pi )\\ & \;=\prod_{\;j=1}^{l}(\prod_{s=1}^{N-1}\left(\prod_{k=1}^{2m_{s}-n_{s}}{\left(Pe^{r_{Z_{s}}t_{k}^{s}\Lambda }P^{-1}\right)}_{X_{\;j,(s,k)},X_{\;j,pa(s,k)}}\right)\\ & \;\cdot \prod_{k=1}^{2m_{N}-2}{\left(Pe^{r_{Z_{N}}t_{k}^{N}\Lambda }P^{-1}\right)}_{X_{\;j,(N,k)},X_{\;j,pa(N,k)}}\\ & \;\cdot \pi (X_{\;j,(N,2m_{N}-1)})), \end{array}$$

where $X_{\;j,(s,\cdot )}$ contains base pair information at position *j* of the locus for all sequences recorded in species *s*, and $X_{\;j,(s,k)}$ for sequence *k* in *s*. $t_{k}^{s}$ is the branch length from gene node (s,k) to pa((s,k)). $X_{\;j,(s,k)}$ is the $j^{\mathrm{th}}$ base pair in the $k^{\mathrm{th}}$ sequence entering species *s* when $k=1,\ldots ,m_{s}$, and is the $j^{\mathrm{th}}$ base pair in gene node (s,k) generated by the $(k-m_{s}{)}^{\mathrm{th}}$ coalescent event in species *s* when $k=m_{s}+1,\ldots ,2m_{s}-n_{s}$.

The posterior distribution of all the latent variables (G, Z, H) and unknown parameters (r,π,Φ) is proportional to the product of the likelihood of the complete data given the latent gene tree G, conservation states Z, and parameters r,π,Φ, and their joint prior distribution. We use MCMC to sample from the posterior distribution and make posterior inference.

### Estimating Population Size Parameters

PhyloAcc-GT requires an estimate of the population size for each species, θ, which can be challenging in many cases. Some approaches (Rannala and Yang 2017; Flouri et al. 2018) provide direct estimates of θ for both current (when more than one allele per extant species is sampled) and ancestral species; other approaches, such as the “two-step” species tree methods, which are helpful in cases of large, genome-wide datasets, estimate branch lengths in coalescent units ($t/2N_{e}$), from which θ could be extracted if one knows the number of generations per branch (Degnan and Rosenberg 2009; Liu et al. 2010, 2015; Mirarab et al. 2014). Additionally, whereas some phylogeographic approaches for estimating ancestral population sizes can benefit from the information from multiple loci (Flouri et al. 2018), here we try to estimate rate parameters for a single locus, which alone cannot yield robust estimates of branch-specific population sizes. In our approach, we estimate genome-wide θ first, then treat θ as a fixed input that we condition on to estimate other parameters.

For a given branch on a tree, PhyloAcc-GT requires a length $l_{1}$ in units of expected number of substitutions per site. This is a common output of phylogenetic software packages (e.g., RAxML: Stamatakis 2014, IQ-TREE: Nguyen et al. 2015) and, if estimated from unconstrained sites, can be related to the neutral substitution rate as $l_{1}=t\mu$, where *t* is the number of generations. Other software such as MP-EST (Liu et al. 2010) and ASTRAL (Mirarab et al. 2014) estimate branch lengths in coalescent units, which are defined with respect to the number of generations *t*. For a given branch, the length in coalescent units is $l_{2}=t/(2N_{e})$. Using these two definitions of branch length, we estimate θ at branch *l* as: $\hat{\theta_{l}}=2l_{1}/l_{2}$. For all extant species, θ is set to 0 as only one sequence per extant species is usually available, and θ for the root node is set as the average θ values among the internal branches of the species tree. PhyloAcc-GT performs this calculation internally both with the species tree provided by the user, with branch lengths in units of expected substitutions per site under the neutral rate, as well as with a topologically identical species tree with branch lengths in coalescent units estimated using one of the methods mentioned above. If this second tree is not pre-estimated, PhyloAcc-GT automates its estimation with a Snakemake (Mölder et al. 2021) pipeline that uses IQ-TREE to estimate individual locus trees for up to 5,000 of the longest input loci and ASTRAL to obtain branch lengths in coalescent units.

### MCMC Procedure for Posterior Inference

Here, inferring the substitution rates r and the conservation states Z for each lineage are of the greatest interest, allowing us to identify the most probable pattern of substitution rate shifts along the phylogeny for each locus. However, other variables, for example, the gene tree G and the ancestral sequences H, cannot be easily integrated out. As such, we use collapsed Gibbs sampling (Liu 1994) to make posterior inference of all parameters. For each locus, we iteratively impute ancestral DNA sequences H and, conditional on the imputed H, sample conservation states Z, substitution rates r, the stationary distribution of base frequencies π, gene trees G, and the hyperparameters from their conditional posterior distributions.

We use the forward–backward (Felsenstein 1973) algorithm to compute conditional likelihoods and sample Z and H, and use the MH algorithm to sample r. Because the substitution rate matrix ***Q*** depends on $\pi_{A}$, we employ the MH algorithm to sample the posterior distribution of π.

When proposing a new gene tree G for a given locus, we use two MH moves (supplementary fig. S2, Supplementary Material online). The first move proposes to change the tree topology of the locus. We randomly select a gene tree branch *s*, disconnect the subtree rooted at *s* from the remaining tree, and graft it back at a new position in the remaining tree. When proposing the new position, we use the already imputed ancestral sequences H to compute transition probabilities of the sequence from all candidate grandparent nodes compatible with the species tree and the current gene tree structure to *s*. A candidate node is chosen with probability proportional to its transition probability. Such a proposed move takes into account both the sequence information and the tree structure. Second, we update gene tree branch lengths locally by shifting the height of each internal node in the gene tree without altering the gene tree topology using an MH algorithm with uniform proposals centering around the current node position. The correctness of the MCMC algorithm is supported by the analysis in supplementary material S3, Supplementary Material online.

The strategy of subtree pruning and re-grafting for updating the tree topology has been explored previously (Rannala and Yang 2003, 2017). However, to the best of our knowledge, our design is the first to utilize sequence information to guide the MCMC move directly. Rannala and Yang (2003) randomly select a feasible branch to graft back to, while Rannala and Yang (2017) prefer smaller topological changes by selecting a new position with probability inversely proportional to the number of nodes on the path to the dissolved branch. Felsenstein et al. (1999) use the gene tree conditional prior distribution as the proposal distribution, which would result in lower efficiency as sequence length increases.

### Detecting and Reconstructing Patterns of Acceleration Based on Bayes Factors and Estimated Conservation States

PhyloAcc-GT fits up to three nested models to each input alignment and selects the best one based on marginal likelihoods (Bayes factors) of the models.

When a set of target lineages are specified, we run all three models. Under the null model $M_{0}$, we assume no species is in the accelerated state. Under the lineage-specific model $M_{1}$, we only allow lineages leading to specified target species to potentially be in the accelerated state. Finally, we run a full model, $M_{2}$, allowing all species not in the outgroup to potentially be in the accelerated state. We identify target lineage-specific accelerations from loci that best fit $M_{1}$ based on two Bayes factors: $BF1=\frac{P(Y|M_{1})}{P(Y|M_{0})}$, which reflects support for the target-restricted model compared to the conserved model, and $BF2=\frac{P(Y|M_{1})}{P(Y|M_{2})}$, which reflects support for the target-restricted model compared to the unrestricted model. Loci with BF1 and BF2 greater than some prespecified thresholds larger than 1 favor the lineage-specific model ($M_{1}$), and are most likely to have experienced target lineage-specific accelerations.

PhyloAcc was originally designed to identify convergent rate shifts related to phenotypic convergence, under which it was proven to outperform existing methods. Under such scenarios, target lineages consist of all extant species having the convergent phenotype. However, PhyloAcc can be used more generally, and allows users to specify any combination of lineages as the target set and identify loci that are accelerated within target lineages, or to provide no target lineages to see which loci are best explained by $M_{2}$. In our application here, as previously (Hu et al. 2019), we do so while also satisfying the condition of Dollo irreversibility of acceleration. For the analyses in this paper, we have elected to retain the assumption as a fair comparison with the original PhyloAcc paper. In cases of convergent evolution, such as those of flightless birds and marine mammals presented here and in the original PhyloAcc paper, we posit that the Dollo assumption makes sense because we want to detect elements similarly accelerated in convergent lineages. This is especially true for loss of flight in birds. By assuming Dollo irreversibility, we also restrict ourselves to a smaller search space of all possible patterns of acceleration, and thereby gain statistical power, especially when the sequence length is short. This approach might actually be favored in many real-world situations. On the other hand, our software is capable of running PhyloAcc(-GT) models with or without the Dollo assumption, based on user choice (see Discussion section).

The identified loci that favor $M_{1}$ can have varying patterns of acceleration, because not all species in the target group are necessarily accelerated. We identify accelerated lineages by filtering out $P(Z_{s}|Y,T,M_{1})\geq 0.5$ or higher for each lineage *s* in the target group inferred under $M_{1}$. Patterns of acceleration can be similarly inferred based on $P(Z_{s}|Y,T,M_{2})$ for loci favoring $M_{2}$ with or without an input target set.

When a target set is not specified, we recommend running both model $M_{0}$ and $M_{2}$ to detect loci experiencing rate acceleration in any lineage. Loci having $BF3\;:\;\equiv \frac{P(Y|M_{2})}{P(Y|M_{0})}=\frac{BF1}{BF2}$ greater than some threshold (at least 1) are likely to have experienced accelerations in some branches of the tree. The precise pattern of acceleration can be inferred from the Z vector estimated under $M_{2}$, in the same way as under $M_{1}$, and they imply potential commonalities among accelerated lineages that may not have previously been evident.

To compute the marginal likelihood of the observed sequences under each model, we need to integrate out both the gene tree topology and the branch lengths. We use the Wang-Landau mixture method in Dai and Liu (2020) to estimate marginal likelihoods of the three models, which are in turn used to calculate the Bayes factors. This method works well for both continuous and discrete latent variables. We partition Y into equally sized data blocks, $Y^{1},\ldots ,Y^{b}$, and recursively apply the Wang-Landau mixture method with a sequence of target and surrogate distributions. In the first step, we take the prior distribution as the surrogate distribution and $P(Z,r,G,\Phi ,\pi |Y^{1},M)$ as the target distribution to estimate $P(Y^{1}|M)$. In the subsequent step *i*, the target distribution from the previous step $P(Z,r,G,\Phi ,\pi |Y^{1\;:\;i-1},M)$ becomes the new surrogate distribution and $P(Z,r,G,\Phi ,\pi |Y^{1\;:\;i},M)$ becomes the new target distribution. In the last step, we get an estimate of P(Y∣M).

### Simulating Sequence Data

**Fig. 2.**
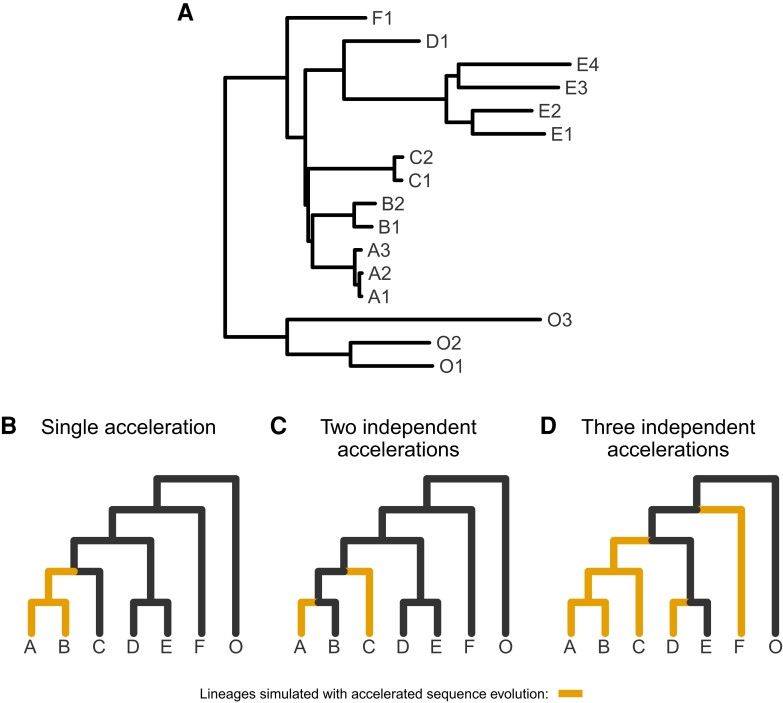
Trees representing simulated scenarios of accelerated sequence evolution. (*A*) The full tree used for simulations with topology and branch lengths based on the ratite phylogeny (supplementary fig. S2, Supplementary Material online). For visualization only, (*B*–*D*) represent collapsed versions of the tree in *A* with arbitrary branch lengths and tip labels representing monophyletic clades. (*B*) A single monophyletic acceleration. (*C*) Two independent accelerations. (*D*) Three independent accelerations.

To test the accuracy of PhyloAcc-GT and compare it to other methods, we simulated sequence data given a species tree under several scenarios of substitution rate acceleration, where we allow either a single monophyletic acceleration, two independently accelerated clades, or three independently accelerated clades (fig. 2). The full species tree used in the simulations is shown in figure 2*A*, with both tree topology and branch lengths borrowed from a ratite tree. Species O1, O2, and O3 are the outgroups. We simulated sequences using the “SIMULATE” function in PhyloAcc-GT. The SIMULATE function takes as input a species tree with branch lengths in expected number of substitutions, population size parameters, a DNA substitution stationary distribution, and a rate matrix ***Q***. For each locus, the function first generates a gene tree according to the multispecies coalescent model (see supplementary material S4, Supplementary Material online), and the DNA sequence at the root of the gene tree following a simulated stationary distribution based on the Beta distribution: $2\pi_{A}∼Beta(10,10)$. Subsequent sequences are generated using the continuous-time Markov model, but only those for extant species are output. The conserved and accelerated rates are generated from Gamma distributions: Gamma(5,0.04) and Gamma(10,0.2), respectively. The two distributions correspond to a mean rate of 0.2 and 2. More simulation analysis using different priors are detailed in supplementary material S7, Supplementary Material online. The population size parameters for the simulations are estimated from real data based on ratites (see below). For our simulations, we first simulated 400 loci with conserved rates in every lineage. Then, for each scenario outlined above, we combined these 400 loci with up to 100 loci simulated with accelerated substitution rates in the specified lineages. All loci are simulated to be 100 base pairs (bp) long.

We used these simulated datasets in several ways to compare PhyloAcc-GT’s accuracy in identifying both genomic loci experiencing acceleration and lineages harboring those loci that are accelerated. First, we calculated the area under the precision-recall curve (AUPRC) based on BF1. Precision is the proportion of true positives out of all called positives. Recall is the percentage of true positives identified out of all true positives. When a dataset contains many more negatives (i.e., loci without any acceleration along the tree) than positives (i.e., loci having at least one acceleration event on a target lineage), the precision-recall curve has been shown to be a more informative measure of a method’s performance than receiver operating characteristic (ROC) curves (Davis and Goadrich 2006). AUPRC varies as a function of the proportion of positives in the dataset (Saito and Rehmsmeier 2015), measuring model performance under different degrees of data skewness. We therefore vary the ratio of the number of accelerated to the number of conserved conserved loci from 1 to 100, and compare AUPRC between PhyloAcc-GT and the original PhyloAcc species tree model (henceforth just “PhyloAcc”).

We also examined how well PhyloAcc-GT identifies specific lineages with accelerated substitution rates under the optimal model inferred. Here, we compared the performance of PhyloAcc-GT, PhyloAcc, and the random local clock model implemented in *BEAST2 (Ogilvie et al. 2017). *BEAST2 also estimates substitution rates along a phylogeny within a Bayesian framework, but does not restrict rate variation to three distinct classes. Because *BEAST2 does not explicitly calculate the probability of acceleration per lineage for a given locus, to compare the performance of *BEAST2 with that of PhyloAcc-GT and PhyloAcc, we estimate P(Z=2∣Y) by the proportion of MCMC outputs in which the branch is accelerated. We treat a branch to be in the accelerated state if its estimated rate is greater than the estimated rate of its parent branch, or if its estimated rate equals that of its parent, and its parent is in the accelerated rate. *BEAST2 does not require input θ, but models and integrates out population size. However, for a fair comparison with PhyloAcc-GT, we input and fix the θ parameters to *BEAST2 as well. We also input and fix the species tree when running *BEAST2. More details on identifying acceleration from results by *BEAST2, as well as results using several alternative criteria to identify accelerations in *BEAST2’s results can be found in supplementary material S6, Supplementary Material online.

To test how PhyloAcc-GT handles phylogenetic discordance, we varied **θ** in our simulated data. When **θ** increases, the mean and variance of coalescent times between sister lineages on the tree increase, leading to an increased probability of discordance. We multiplied the θ values estimated from the ratite data by 3, 6, or 10 and use these new parameters to simulate new sequences under the three previously described scenarios. We also tested the robustness of PhyloAcc-GT to θ mis-specifications.

### Ratite and Marine Mammal Data

To further compare PhyloAcc-GT with PhyloAcc, we use data from two systems: birds and mammals. We previously analyzed these data with PhyloAcc and identified genomic loci associated with loss of flight in birds (ratites) and the transition to aquatic lifestyles in mammals (marine mammals) (Hu et al. 2019). The bird dataset consists of 43 species, including 9 flightless birds (ratites: ostrich, moa, 2 species of rhea, emu, cassowary, and 3 species of kiwi), 27 volant bird species, and 7 reptiles as outgroup species (supplementary fig. S2, Supplementary Material online). We used the alignment of 284,001 conserved noncoding loci, the species tree, and genome-wide estimates of neutral substitution rates from Sackton et al. (2019) and Hu et al. (2019).

For the mammal data, we previously used the alignments of 283,369 conserved noncoding loci from 62 species (Hu et al. 2019), a species tree (supplementary fig. S3, Supplementary Material online), and genome-wide estimates of neutral substitution rates from the UCSC 100-way vertebrate alignment (Blanchette et al. 2004). We identified conserved noncoding loci using PHAST (Hubisz et al. 2011) and estimated neutral substitution rates from 4-fold degenerate sites using phyloFit (Hubisz et al. 2011); see Sackton et al. (2019) and Hu et al. (2019) for full description of these methods. From these datasets, since we are interested in comparisons of PhyloAcc-GT with PhyloAcc, we limit our comparisons to the loci previously inferred to be accelerated in either ratites (806 loci based on Bayes factor cutoffs of $\log\;BF_{1}>20$ and $\log\;BF_{2}>0$) or marine mammals (2,106 loci based on Bayes factor cutoffs of $\log\;BF_{1}>5$ and $\log\;BF_{2}>5$) (Hu et al. 2019).

For both datasets, we estimate Θ based on the species tree topology as described above, using gene trees from 20,000 randomly selected loci. For each set of gene trees, we ran MP-EST 5 times and used the branch lengths from the run with the maximum likelihood. $\hat{\Theta }$ is then calculated based on the branch lengths of the two trees (one with branch lengths in units of relative number of substitutions and one with branch lengths in coalescent units) as outlined in the section above (Estimating Population Size Parameters section). We repeated this process 50 times and averaged the θs as the population size parameters for each dataset. We used the estimates from the ratite data as $\hat{\Theta }$ for the simulated datasets described above.

We observe that the estimated θ’s exhibit small variations across 50 estimations using different subsets of loci. For example, in the mammal data set, sample standard deviations range from 0.94% to 5% of sample means in 11 branches. Only 1 branch has larger variation: the standard error is 12.7% of the mean. Thus, with different runs of algorithms RAxML and MP-EST, we achieved θ estimates in the range $\left[0.85\hat{\theta },1.15\hat{\theta }\right]$ most of the time.

### Site Concordance Factors

Because our ILS-aware method requires significantly greater computing time than PhyloAcc, we use site concordance factors (sCF) to determine on a locus-by-locus basis whether to use the PhyloAcc-GT method, which accounts for phylogenetic discordance in the input locus, or the original PhyloAcc species tree method, which uses only a single species tree for all loci. Concordance factors (Ané et al. 2007; Baum 2007) were first implemented on a per-site basis by Minh, Hahn, et al. (2020) in IQ-TREE2 (Minh, Schmidt, et al. 2020) to summarize discordance among genes relative to a species tree. Briefly, sCF is calculated for a given branch in the species tree by first calculating concordance factors among sub-alignments of quartets of species sampled from that branch (CF${}_{q}$). For each quartet, we count the number of sites in the alignment of those species that match the topology in the species tree [e.g., ((A,A),(G,G))] and divide that number by the total number of decisive alignment sites (see Minh, Hahn, et al. 2020, eq. 2). In IQ-TREE-2 (Minh, Schmidt, et al. 2020), these values of CF${}_{q}$ are calculated over all sites in every input alignment and averaged to obtain an overall summary of discordance in the dataset. Here, we re-implement the sCF calculation to be applied to each individual locus, resulting in a value for each branch in the species tree for each locus. We then use the sCF values for each locus to guide the selection of the PhyloAcc gene tree or species tree method. This can be specified in two ways by the user: 1) if the average of all sCF values for the locus are below some threshold this locus will be run with the gene tree method, otherwise it will be run with the species tree method and 2) if the proportion of branches with a sCF below some threshold exceeds another threshold, this locus will be run with the gene tree method, otherwise it will be run with the species tree method. Thresholds are specified with user inputs and are meant to limit the number of loci run with the computationally more intensive gene tree method.

### Benchmarking With Simulated Data

We benchmarked both the PhyloAcc-GT and PhyloAcc species tree algorithms by using simulated datasets. We simulated loci on species trees of various sizes (9, 13, or 17 species). For each species tree, we simulated 100 sequences of various length (100, 200, 400, and 600 bp) and ran each locus through both programs in batches of 10 loci with each batch using 4 threads. We measured average run time and average maximum memory use on each batch and divided by batch size to get average resource use per locus. We ran these benchmarks on the Harvard Research Computing Cannon Cluster.

## Results

The PhyloAcc-GT algorithm is implemented in a C++ codebase that accounts for phylogenetic discordance in the input loci while estimating substitution rates across a phylogeny. This algorithm, along with the original PhyloAcc codebase, which uses a single species tree for all input loci, and a newly implemented command-line user interface, are packaged together to form the PhyloAcc software (https://phyloacc.github.io/). The user interface is implemented in Python and provides the ability to easily batch input loci into separate runs for PhyloAcc, which can be partitioned between the species tree and gene tree methods. These batches are then executed via an automatically generated Snakemake (Mölder et al. 2021) file that can submit batches in parallel as separate jobs to a high-performance computing cluster with job scheduling software (e.g., SLURM).

### Model Performance With Correct Input Targets

**Fig. 3.**
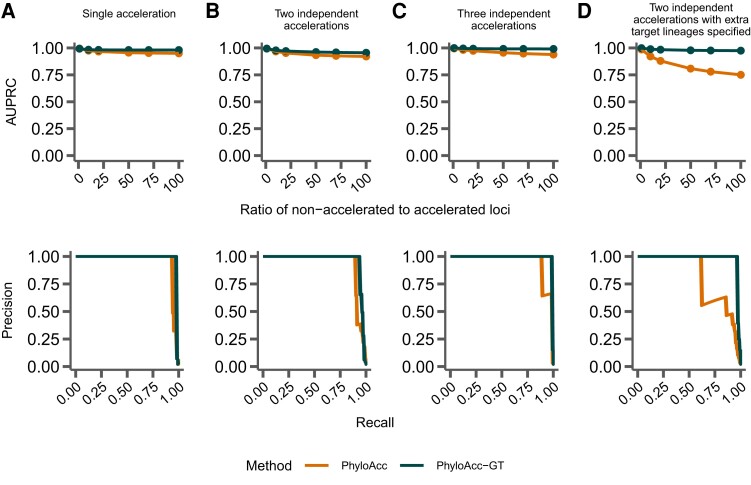
Comparing performance between PhyloAcc and PhyloAcc-GT. The top row shows the AUPRC while varying the ratio of simulated conserved to accelerated loci. The bottom row shows a single precision-recall curve at a ratio of 50 conserved loci per accelerated locus. In *A*–*C*, the specified target lineages match those lineages on which accelerations were simulated. (*A*) Loci simulated with a single monophyletic acceleration. (*B*) Loci simulated with two independently accelerated clades. (*C*) Loci simulated with three independently accelerated clades. (*D*) Loci simulated with two independently accelerated clades, but with additional target lineages provided to each method.

To measure their ability to differentiate loci in the accelerated state from loci not in the accelerated state with respect to a set of target lineages, we input the correct (i.e., simulated) target set to PhyloAcc-GT and PhyloAcc, using three sets of simulated data (single accelerated clade; two independent accelerations; and three independent accelerations; see fig. 2). We then measure the AUPRC of logBF1 while varying the proportion of loci in the accelerated state. We find that PhyloAcc-GT has high precision and recall as measured by AUPRC (fig. 3). As the proportion of target-specific accelerated loci decreases, it becomes harder to detect these loci from the remaining conserved ones because more conserved loci can be falsely identified as in the accelerated state at any fixed logBF1 cutoff. However, the AUPRC for PhyloAcc-GT never falls below 95% regardless of the type of acceleration scenario or the fraction of input loci having target lineage in the accelerated state (fig. 3). By contrast, the original PhyloAcc always has a lower AUPRC, especially when lineages that are truly in the accelerated state are a subset of the input targets (e.g., fig. 3*D*). When the ratio of conserved to accelerated loci is 100:1, PhyloAcc-GT can identify true positive cases more than 95% of time, while PhyloAcc’s performance can drop to 75%. The precision-recall curves at ratio 50:1 conserved to accelerated loci are also shown in figure 3. In all three simulated cases, PhyloAcc-GT also estimate more accurate rates than PhyloAcc (supplementary material S5, Supplementary Material online).

**Fig. 4.**
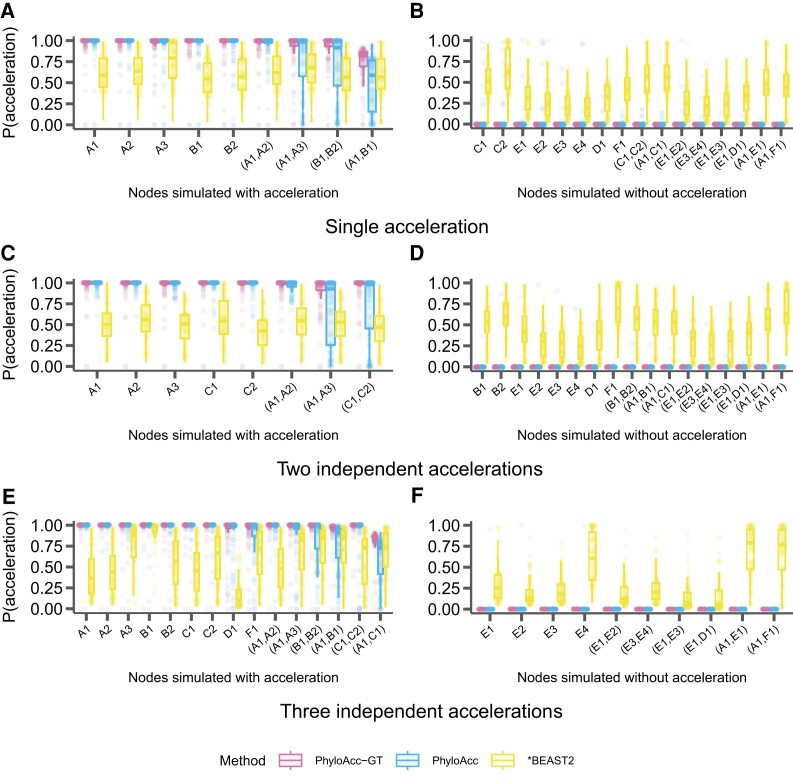
Comparison of the identification of lineage-specific rate accelerations between three methods, PhyloAcc-GT (leftmost boxplot for each branch on the x-axis), PhyloAcc (middle boxplot for each branch on the x-axis), and *BEAST2 (rightmost boxplot for each branch on the x-axis) when the input target lineages match lineages that are truly in the acccelerated state. Each distribution corresponds to the estimated P(Z=2∣Y)s of a branch from 100 simulated loci. Branches are indicated on the x-axis of each plot and correspond to those in figure 2*A*. Distributions on the left correspond to lineages simulated to have accelerated sequence evolution in each of the three scenarios in figure 2, whereas distributions on the right correspond to those without accelerated sequence evolution. (*A* & *B*) The probability of being in the accelerated state for each locus and lineage using sequences simulated with a single accelerated clade (fig. 2*B*). (*C* & *D*) Probability of being in the accelerated state for each locus and lineage using sequences simulated with two independently accelerated clades (fig. 2*C*). (*E* & *F*) Probability of being in the accelerated state for each locus and lineage using sequences simulated with three independently accelerated clades (fig. 2*D*).

In addition to assessing model selection accuracy by locus, we also check for accuracy of predicting lineages in the accelerated state by examining the posterior probability of having the accelerated rate in each branch P(Z=2|Y) under the most favored models based on Bayes Factors. We find that both PhyloAcc-GT and PhyloAcc can precisely identify terminal branches that are in the accelerated state. However, PhyloAcc-GT is much better at identifying internal branches of the tree that are in the accelerated state than PhyloAcc (fig. 4). Under the multispecies coalescent, gene tree branch lengths for extant species are longer than the branches of the species tree, whereas the same is not necessarily true for internal branches (fig. 1). As such, PhyloAcc tends to overestimate substitution rates along terminal branches more than along internal branches.

We also compare the ability of PhyloAcc-GT to detect lineages in the accelerated state to *BEAST2. We find that *BEAST2 reports lower posterior probabilities for being in the accelerated state for most positive branches (i.e., branches that are truly in the accelerated state) than both PhyloAcc-GT and PhyloAcc (fig. 4*A*, *C*, and *E*). The average estimated posterior probabilities of being in the accelerated state across positive branches are 0.62 for the single acceleration case, 0.59 for two accelerated clades, and 0.5 for three accelerated clades. These values, while generally over 0.5, fall below a conservative threshold that one may use to identify accelerated lineages. Additionally, *BEAST2 has less resolution in discerning positive lineages from the rest, with several lineages not in the accelerated states having an average posterior probability of being in the accelerated state above 0.5, which may lead to a higher false positive rate (FPR) in detecting loci in the accelerated state on a given branch (fig. 4*B*, *D*, and *F*).

### Model Performance With Mis-specified Targets

**Fig. 5.**
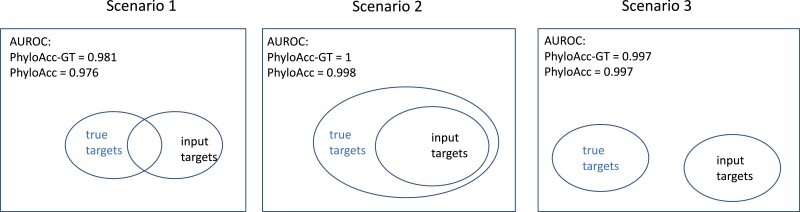
Scenarios for testing model performance with mis-specified targets, along with area under the ROC for both PhyloAcc-GT and PhyloAcc.

To test the ability of PhyloAcc-GT to distinguish target-specific acceleration from acceleration in nontarget branches using logBF2, we consider three scenarios where the specified target lineages include only some or none of the lineages that are simulated to be in the accelerated state (fig. 5). In scenario 1, the input target species partially overlap species that are truly in the accelerated state: we simulate two independently accelerated clades, and specify one of them as the target lineage and the other as a nontarget clade. In scenario 2, the input target species are a subset of species that are simulated to be in the accelerated state: we simulate three independently accelerated clades, and specify as targets only one of those clades. In scenario 3, the species that are simulated to be in the accelerated state do not intersect with input target species. Area under the ROC (AUROC) curve between PhyloAcc and PhyloAcc-GT are recorded in figure 5’s legend. We use AUROC to measure model performance because the input set of targets and specified set of targets can be any two acceleration patterns. It is reasonable to not assume that loci in the accelerated state under one pattern (the input target set under model $M_{1}$) are significantly more frequent than the other (the input target set under Model $M_{2}$). Both methods are highly accurate in excluding nonspecific accelerated loci. AUROC are close to 1 as presented in table 1. We also compute the true positive rate (TPR) at 1% and 5% FPR cutoffs. In all scenarios, PhyloAcc-GT has higher accuracy than PhyloAcc.

**Table 1.** Comparing TPR at Different FPR Cutoffs Using logBF2 to Distinguish Target-Specific Accelerated Loci From Nontarget-Specific Accelerated Loci Under Different Scenarios of Target Mis-specification Between PhyloAcc-GT and PhyloAcc.

Testing	Method	TPR	TPR
Scenario		@1%FPR	@5%FPR
1	PhyloAcc-GT	0.89	0.96
	PhyloAcc	0.76	0.84
2	PhyloAcc-GT	0.99	1
	PhyloAcc	0.92	1
3	PhyloAcc-GT	0.97	0.98
	PhyloAcc	0.94	0.97

Note.—Species that are truly in the accelerated state either overlap (rows 1 & 2), include (rows 3 & 4) or are completely different from input target species (rows 5 & 6).

Next, we assess the inference of conservation states, specifically P(Z=2|Y), or the probability of being in the accelerated state along a given branch, of all branches by PhyloAcc-GT and PhyloAcc under the above scenarios of target mis-specification. Results using *BEAST2 are not presented because it does not allow prior selection of targets.

**Fig. 6.**
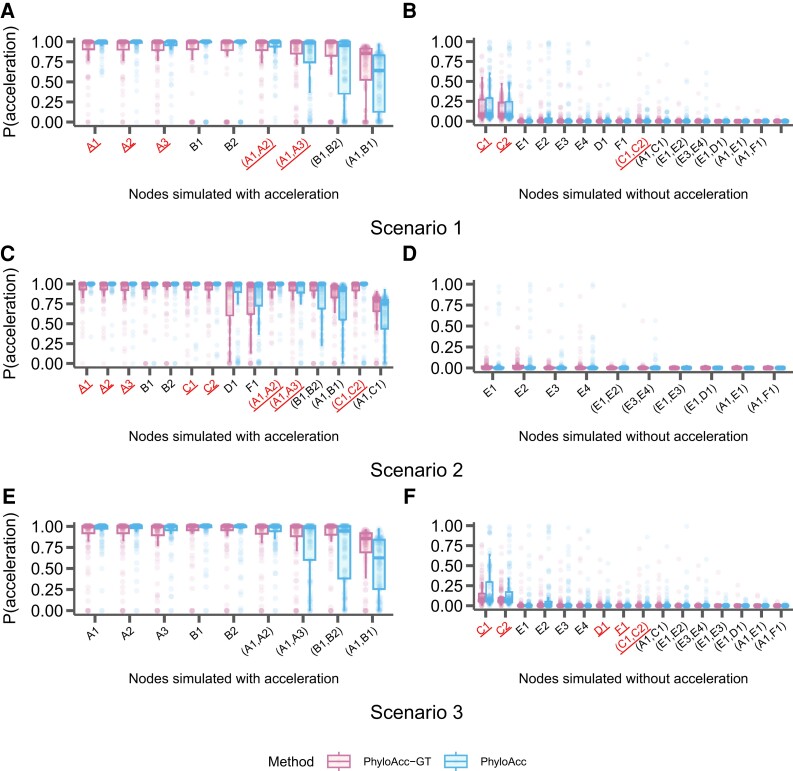
Distributions of the probability of being in the accelerated state [P(Z=2∣Y)] for each branch in the input species tree when specified target lineages are mis-specified. Branches are indicated on the x-axis of each panel and correspond to those in figure 2*A*. Distributions on the left correspond to lineages simulated to have accelerated sequence evolution in each of the three scenarios in figure 2, and distributions on the right correspond to those without accelerated sequence evolution. Branches underlined on the x-axis are those that were specified as target lineages for M1 in each run of PhyloAcc or PhyloAcc-GT and the three scenarios correspond to those outlined in figure 5. Each point represents one simulated locus. (*A* & *B*) The probability of being in the accelerated state using sequences simulated with a single monophyletic acceleration (fig. 2*B*) and targets specified that partially overlap lineages that are truly in the accelerated state. (*C* & *D*) The probability of being in the accelerated state using sequences simulated with two independent accelerations (fig. 2*C*) and targets specified as a subset of lineages that are truly in the accelerated state. (*E* & *F*) The probability of being in the accelerated state using sequences simulated with three independent accelerations (fig. 2*D*), and no lineages that are truly in the accelerated state being specified as targets.

We find that PhyloAcc-GT is more accurate in identifying branches in the accelerated state than PhyloAcc (fig. 6). Although PhyloAcc-GT produces a slightly wider range of probabilities of being in the accelerated state across lineages that are truly in the accelerated state than PhyloAcc, almost all probabilities are still above 0.75. Consistent with the previous analysis, PhyloAcc-GT performs much better than PhyloAcc in detecting internal branches in the accelerated state. For branches not in the accelerated state, both methods tend to have higher estimated posterior probabilities of being in the accelerated state in clade C compared to other species that are not in the accelerated state (e.g., scenario 1). The higher probabilities are probably due to the shorter branch lengths of C1 and C2, and their proximity to branches that are truly in the accelerated state. Compared with the case of a single acceleration in figure 4 when $M_{1}$ is the true model, correctly identifying $M_{1}$ in PhyloAcc-GT or PhyloAcc can reduce the posterior probability of being in the accelerated state in branches that are not in the accelerated state. However, as these posterior probabilities are still below 0.5 in most loci, the ability in inferring the correct acceleration pattern and the number of independent acceleration events is largely not affected by the input target species.

### Identifying Accelerated Lineages With No Input Target Set

Although a model that tests for being in the accelerated state on specific target lineages may prove a better fit than a full model, often this information is unavailable, or we may want to ask general questions about our sample (e.g., “How many loci are in the accelerated state in any lineage?”, “Which lineages have the most loci that are in the accelerated state?”). To test PhyloAcc-GT’s performance under such scenarios, we use the same set of simulations as previously described (fig. 2), but now use logBF3 to identify loci that fit $M_{2}$, and then use P(Z=2∣Y) to reconstruct the patterns of acceleration.

**Fig. 7.**
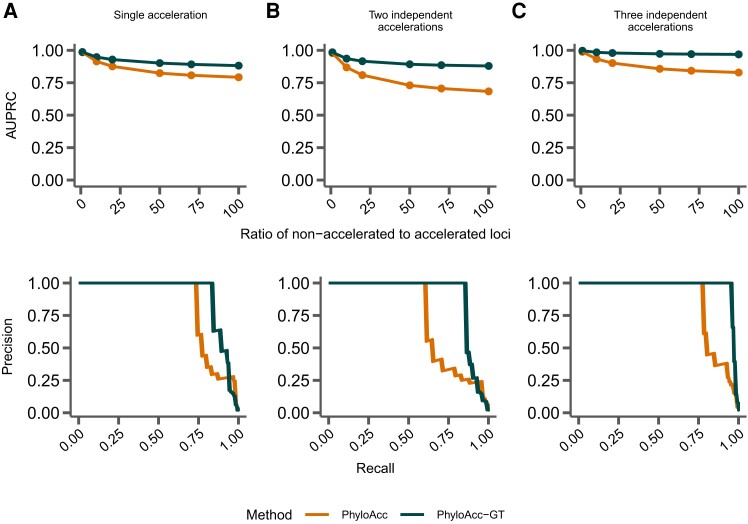
Comparing performance between PhyloAcc and PhyloAcc-GT without specifying target lineages. The top row shows AUPRC for both methods while varying the ratio of number of loci that are in nonaccelerated state to accelerated state. The bottom row shows a single precision-recall curve at a ratio of 50 loci in the nonaccelerated state per locus that is in the accelerated state. (*A*) Loci simulated with a single, monophyletic acceleration. (*B*) Loci simulated with two independent accelerations. (*C*) Loci simulated with three independent accelerations.

**Fig. 8.**
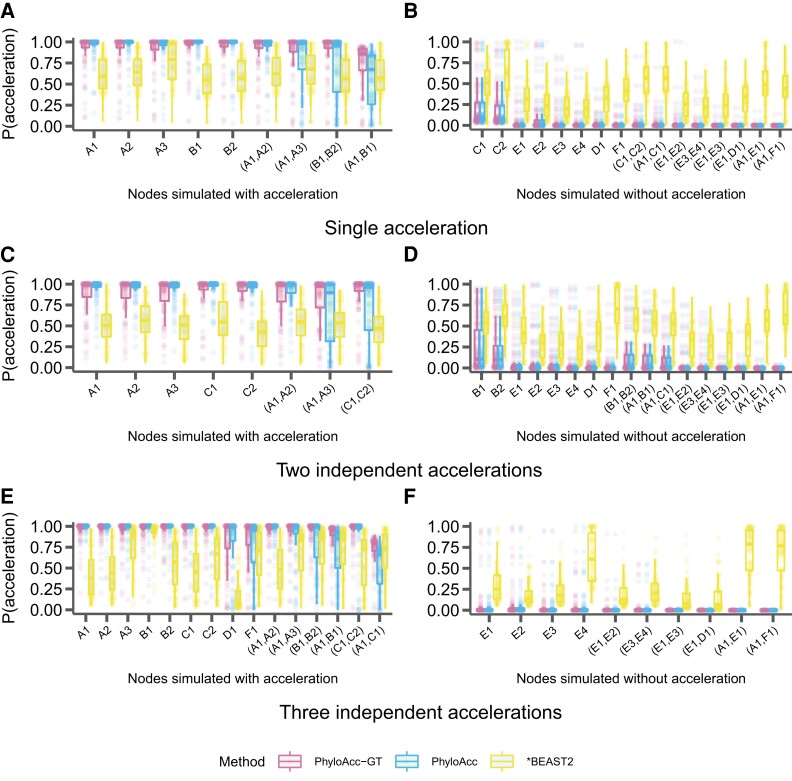
Comparison of the identification of lineage-specific rate accelerations between three methods, PhyloAcc-GT (leftmost boxplot for each branch), PhyloAcc (middle boxplot for each branch), and *BEAST2 (rightmost boxplot for each branch), when no target lineages are provided (i.e., from $M_{2}$). Each distribution corresponds to the estimated P(Z=2∣Y)s of a branch from 100 simulated loci. Branches are indicated on the x-axis of each plot and correspond to those in figure 2*A*. Distributions on the left correspond to lineages simulated to have accelerated sequence evolution in each of the three scenarios in figure 2, whereas distributions on the right correspond to lineages simulated without accelerated sequence evolution. (*A* & *B*) The probability of being in the accelerated state for each locus and lineage using sequences simulated with a single accelerated clade (fig. 2*B*). (*C*& *D*) Probability of being in the accelerated state for each locus and lineage using sequences simulated with two independent accelerations (fig. 2*C*). (*E* & *F*) Probability of being in the accelerated state for each locus and lineage using sequences simulated with three independent accelerations (fig. 2*D*).

We again find that PhyloAcc-GT more accurately identifies loci in the accelerated state than PhyloAcc in all scenarios (fig. 7). The differences in performance by the two methods are more pronounced as the percentage of nonaccelerated loci in the data increases, and the performance gap is larger than when testing a set of target lineages with logBF1 (fig. 3). We also find similar patterns in the distribution of P(Z=2∣Y) for branches that are truly in the accelerated state whether we input the correct target set or not (fig. 8 vs. fig. 4). However, when identifying lineages in the accelerated state for a given locus without specifying targets, we see larger variation in P(Z=2∣Y) among branches that are not in the accelerated state but near those in the accelerated state on the species tree (fig. 8*B*, *D*, and *F*, compared to the results when target branches are specified (fig. 4*B*, *D*, and *F*), and branches with short branch lengths in the accelerated state (e.g., clade A). However, these posterior probabilities generally do not exceed 0.5 for branches not in the accelerated state, and are mostly above 0.5 for branches that are truly in the accelerated state. When only a single clade is truly in the accelerated state, we observe more variation in posterior probabilities when an input set is not specified. In this case, when lineages in the accelerated state are correctly specified in the input set, no false positives are observed among 17 branches that are not in the accelerated state under 100 simulations. When using $P(Z=2|Y,M_{2})$, the FPR is 4% and the false negative rate increases from 3% to 9%.

This result implies that specifying a target set is beneficial, and if one has logical target lineages in mind, we recommend using them to reconstruct patterns of acceleration using results from $M_{1}$ for those selected loci, to achieve a slightly lower FPR. However, if an input set cannot be specified, our method still reliably identifies loci that are in the accelerated state and infers patterns of acceleration using $M_{2}$, with only minor reductions in accuracy.

### Robustness to Phylogenetic Discordance

**Fig. 9.**
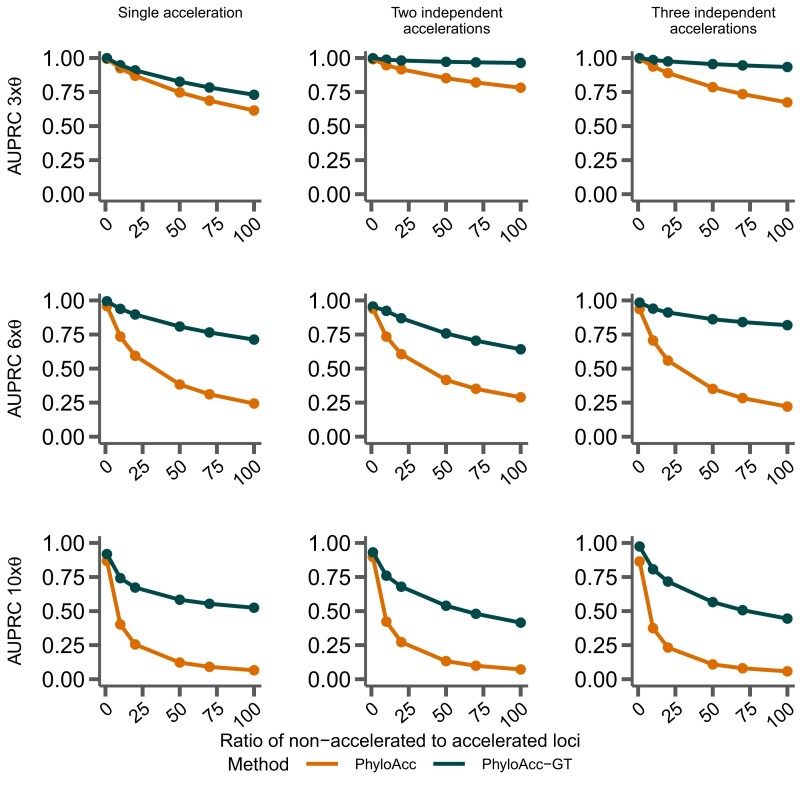
Comparing performance between PhyloAcc and PhyloAcc-GT with varying levels of Θ and the ratios of nonaccelerated to accelerated loci. Rows represent different scales of the θ (3×, 6×, or 10×) values estimated from the ratite dataset (see Methods section), while columns represent different simulation scenarios.

The amount of phylogenetic discordance present within the input loci affects the identification of both loci and lineages experiencing accelerated substitution rates. To assess how PhyloAcc-GT performs with varying levels of phylogenetic discordance due to ILS, we varied the population size parameter **θ** in each of our three simulation cases. We find that in each case when considering logBF1, as **θ** increases the AUPRC of PhyloAcc-GT decreases depending on the fraction of loci that have branches in the accelerated state (fig. 9). However, in every case PhyloAcc-GT achieves a higher AUPRC than PhyloAcc, especially when the θs are large and the proportion of loci having branches in the accelerated state is low.

**Fig. 10.**
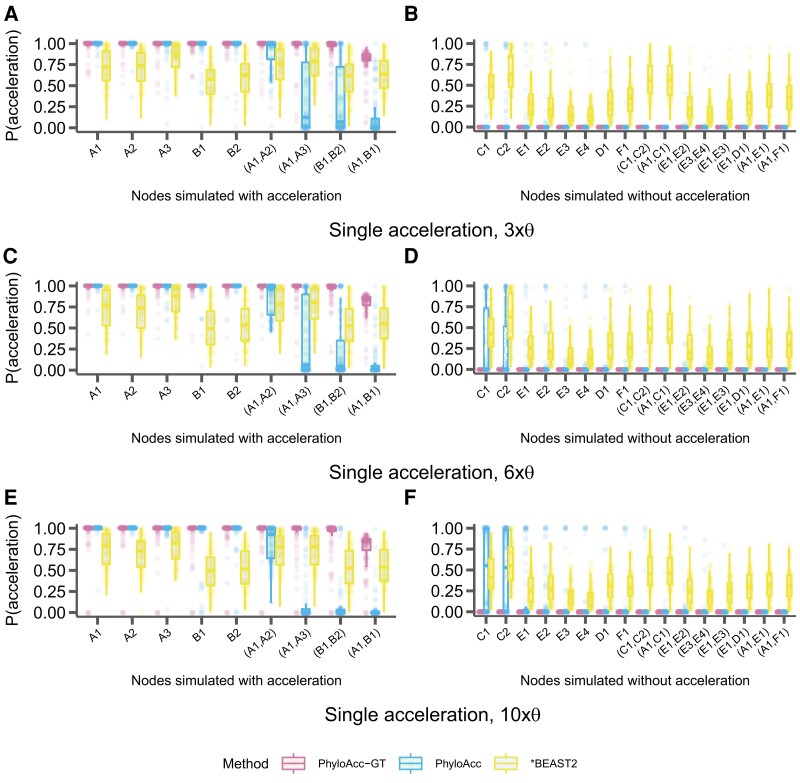
Distributions of the probability of being in the accelerated state [P(Z=2∣Y)] using PhyloAcc-GT (leftmost boxplot for each branch), PhyloAcc (middle boxplot for each branch), and *BEAST2 (rightmost boxplot for each branch) while scaling the population size parameter, Θ. Distributions shown for data simulated with a single acceleration in the *A* and *B* clades (fig. 2*B*). Branches are indicated on the x-axis of each panel and correspond to those in figure 2*A*. Distributions on the left correspond to lineages simulated to have accelerated sequence evolution while the distributions on the right correspond to lineages simulated without accelerated sequence evolution. (*A* & *B*) Sequences simulated with 3 times the expected θ. (*C* & *D*) Sequences simulated with 6 times the expected θ. (*E* & *F*) Sequences simulated with 10 times the expected $\theta^{\prime }$.

We also find that PhyloAcc-GT consistently outperforms PhyloAcc in identifying lineages in the accelerated state while minimizing false positives, regardless of the extent of ILS (figs. 10; supplementary material S40 and S41, Supplementary Material online). For PhyloAcc-GT, the posterior probabilities of branches in the accelerated state are mostly above 0.75 and in most cases close to 1, while the probabilities are close to 0 for branches not in the accelerated state. Again, we see that PhyloAcc also performs quite well when identifying acceleration on terminal branches of the species tree, but its performance on internal branches is greatly affected by the amount of ILS. In many cases, the average posterior probability of being in the accelerated state on an internal branch that is truly in the accelerated state falls below 0.2 and even close to 0 for very high levels of ILS. In general, *BEAST2’s performance does not seem to be affected by varying amounts of ILS. Lineages in the accelerated state also consistently have an average probability of being in the accelerated state > 0.5 when analyzed with *BEAST2. However, in most instances this probability is less than 0.75 and has large variation. *BEAST2 also has a high variance in posterior probabilities of being in nonaccelerated states for branches that are not in the accelerated state, which are routinely between 0.25 and 0.5, and can be up to 0.75 in some branches, possibly leading to false positives.

### Robustness to Mis-specification of Theta

Θ is a key input to PhyloAcc-GT. In simulation studies, we have assumed Θ is known, and is equal to the true Θ used to simulate the sequences. In practice, we do not know the true Θ, so we tested the performance of PhyloAcc-GT when Θ is mis-specified.

To test the robustness of our method to mis-specification of Θ, we conducted experiments using data simulated with both one acceleration and two independent accelerations (see fig. 2*B* and *C* for acceleration patterns). Under each acceleration pattern, we tested 6 cases of Θ mis-specifications. In the first 4 cases, we input θs that are systematically down-scaled or up-scaled from the true θs by a common scaling factor: 0.5, 0.8, 1.5, or 2. In case 5 [Unif(0,2)], each input θ is a random scaling of the true θ, where the random number is sampled from the uniform distribution between 0 and 2. In the last case, we input Θ that is estimated by the procedure described in Estimating Population Size Parameters section. For each test case, we analyzed 100 loci.

Under mis-specification of **θ**, we still identify numerous loci that favor a model of target-specific acceleration with both BF1 and BF2 being positive. We find that PhyloAcc-GT correctly identifies accelerated loci over 97% of the time when the scaling factor of θ is between 0.5 and 2 (our tested cases). At 5% FPR, the TPRs are all above 0.98 across scenarios (table 2).

**Table 2.** Test Sensitivity of PhyloAcc-GT to θ Mis-specifications.

No. Indep.	Scaling	TPR@	TPR@	Average	Average
Acceleration	Factor	1%FPR	5%FPR	${\hat{r}}_{1}-r_{1}$	${\hat{r}}_{2}-r_{2}$
1	0.5	0.98	0.98	0.019	0.063
	0.8	0.98	0.99	0.014	0.027
	1.5	0.97	0.98	0.001	− 0.037
	2	0.90	0.98	− 0.001	− 0.047
	Unif(0,2)	0.94	0.97	0.019	− 0.015
	Estimated θ	0.98	0.98	0.012	− 0.038
2	0.5	0.99	1	0.016	0.075
	0.8	0.98	0.99	0.008	0.008
	1.5	0.97	1	− 0.002	− 0.040
	2	0.97	1	− 0.008	− 0.047
	Unif(0,2)	0.99	0.99	0.001	0.047
	Estimated θ	0.96	0.99	− 0.004	− 0.045

Note.—TPRs at the two FPR cutoffs are computed based on logBF1 among null loci and accelerated loci.

In addition to model selection, estimates of the conserved and accelerated substitution rates, $r_{1}$ and $r_{2}$ respectively, are influenced by θ as well, though in general the biases tend to be small. When we input underestimated θs, the model will overestimate $r_{1}$ and $r_{2}$ and vice versa. When the input value of θ for each branch is a random scaling of the true θ, the direction of estimated bias depends on all the realized θ’s along the tree. When we use the estimated Θ as input, in both acceleration patterns, PhyloAcc-GT tends to underestimate $r_{2}$.

### Identifying Accelerated Loci in Ratites

We applied PhyloAcc-GT to the 806 conserved noncoding loci previously detected by PhyloAcc (Hu et al. 2019) to have strong evidence for ratite-specific acceleration (BF1>20 and BF2>0), possibly linking them to the loss of flight. When accounting for phylogenetic discordance with PhyloAcc-GT, we found that 88% (713) of the loci still favor $M_{1}$, indicating ratite-specific acceleration, whereas 8% (67) of those loci previously identified now fall under $M_{0}$ and do not show any rate acceleration. Examining the 67 loci favoring $M_{0}$, we found that 11 of these loci do not have any target lineage with a high probability to be in the accelerated state [P(Z=2|Y)>0.5] under PhyloAcc (see supplementary material S9, Supplementary Material online).

To determine which loci still show strong evidence of ratite-specific accelerations after accounting for phylogenetic discordance with PhyloAcc-GT, we first determined new Bayes factor cutoffs for the ratite data based on simulated data. We find that the ratio of BF1 between PhyloAcc and PhyloAcc-GT for data generated under $M_{1}$ (two accelerated clades) is 1.8, meaning that BF1 tends to be higher when using PhyloAcc. To account for this, we adjust our BF1 cutoff to identify ratite-specific accelerations when using PhyloAcc-GT from 20 down to 10. The BF2 cutoff remains 0. Using these cutoffs, we identify 509 out of the original 806 loci (63%) with strong evidence for ratite-specific acceleration. The average estimated accelerated rate ($r_{2}$) is 2.5, while the mean conserved rate ($r_{1}$) is 0.16. Eighty-eight percent of these loci have accelerated rate greater than 1, and 56% are greater than 2. Similar to PhyloAcc’s result, the rhea clade is most likely (60%) to experience acceleration among all lineages. Almost all accelerations in this clade are inferred to have occurred in the most recent common ancestor of the two extant rhea species, rather than two independent accelerations. The emu and cassowary branches are the second most likely (40%) lineages to be accelerated, and 80% of the accelerations occurred along their ancestral branch. The ostrich branch is the least likely extant species to have experienced accelerations.

Among accelerated loci, 291 are inferred to have accelerated on only one branch by PhyloAcc-GT. Forty-three percent of these single-branch accelerations occur along the ancestral rhea branch, followed by 11% in moa and 11% in the most recent common ancestor of cassowary and emu. The original PhyloAcc, without considering ILS, detected only 265 single-branch accelerations. In some cases, PhyloAcc inferred separate accelerations in sister branches, whereas PhyloAcc-GT infers only a single acceleration in the ancestral branch of the two sibling branches. For example, PhyloAcc estimates locus mCE1745684 having two independent accelerations in cassowary and emu, whereas PhyloAcc-GT infers the acceleration to have occurred in their parent species.

Recently an alternative but weakly supported species tree for palaeognaths has been advocated, suggesting that rheas are sister to kiwis, emus, cassowaries, and tinamous (Simmons et al. 2022). Re-running PhyloAcc using the alternative tree identifies 817 (log-BF1>20, log-BF2>0 as in Hu et al. 2019) loci being accelerated. Among these loci, 717 loci overlap with the 806 loci (89%) identified using the original tree. For the remaining loci that are detected under the original tree but not in alternative tree, 77 loci still have the maximum marginal likelihood under model $M_{1}$, that is, favoring a pattern of ratite-specific acceleration over no acceleration or acceleration in nonratites. When running PhyloAcc-GT with the alternative tree, PhyloAcc-GT selects $M_{1}$ as the optimal model in 713 loci. Six hundred and seventy-one loci (94%) show evidence of ratite-specific accelerations under both species tree specifications with PhyloAcc-GT, whereas only 89% of loci show the same pattern in both trees with PhyloAcc, indicating that PhyloAcc-GT is more robust to different species tree topologies than PhyloAcc.

### Identifying Accelerated Loci in Marine Mammals

We also re-ran PhyloAcc-GT on 1,276 conserved noncoding loci that were previously inferred to have marine mammal specific accelerations using the original PhyloAcc species tree model with BF1 and BF2 cutoffs of 4 (Hu et al. 2019). We find that 1,034 (81%) loci still have the highest marginal likelihood under model $M_{1}$, while 225 (17.6%) loci now favor the null model. Setting cutoff at 2 for both log Bayes factors, we estimate 882 loci to have strong target lineage-specific acceleration. The average conserved rate is 0.17 and the average accelerated rate is 2.66, with 761 loci having an accelerated rate greater than 1.

Using PhyloAcc-GT, we find that the branch leading to dolphins experiences the largest number of rate accelerations (606), followed by killer whale (539). Additionally, 403 accelerations occurred in the ancestral cetacean branch. These results differ from using the original PhyloAcc model, which identified, only 279 accelerations in the ancestral cetacean lineage. Among the loci identified as accelerated in this branch by PhyloAcc-GT, PhyloAcc is more likely to identify the acceleration in only one of the two extant species (dolphin or killer whale), with 26 loci actually identified as having independent accelerations in both. For example, for locus VCE173687, PhyloAcc estimates a posterior probability of acceleration of 0.89 in the killer whale branch, but only 0.64 in dolphin. However, PhyloAcc-GT infers that there is an acceleration event the ancestral cetatcean branch, and the posterior probabilities of acceleration of the parent and child branches are all greater than 0.88. Other than this difference, inference of conservation states of other target species are the same: both PhyloAcc and PhyloAcc-GT infer an independent acceleration in manatee with posterior probability greater than 0.99, and posterior probabilities of being in the accelerated states for seal and walrus are all below 0.7.

The number of accelerations in manatee, seal, and walrus are 219, 205, and 235, respectively. As opposed to the cetacean clade which has many accelerations in the ancestral branch, in the pinniped clade, most rate shifts happen independently in either the walrus or seal lineages. Only 77 loci are estimated to have experienced one acceleration along the ancestral pinniped branch. This is similar to PhyloAcc’s result: there are 201, 190, and 235 loci accelerated in manatee, seal, and walus, and 65 accelerations in walrus and seal started in their parent species.

### Benchmarking & Implementation

**Fig. 11.**
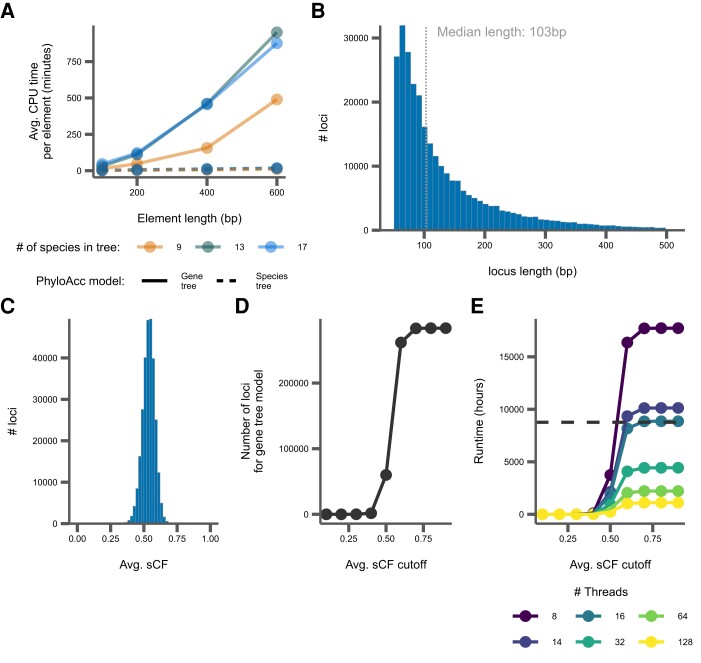
Summaries of benchmarking and concordance factor analysis. (*A*) Average CPU time per simulated locus in minutes. (*B*) Distribution of locus lengths from ratite data, with the median length labeled and indicated by the dotted line. (*C*) Distribution of sCF per locus from ratite data. (*D*) The number of ratite loci that would be run with the gene tree model with various sCF cutoffs. (*E*) The expected run time for all loci to complete with the gene tree model from the ratite dataset with various sCF cutoffs. The solid lines correspond to varying the number of threads per locus. The dashed line corresponds to a time of 1 year.

We benchmarked PhyloAcc-GT and the original PhyloAcc by running the programs on loci simulated on species trees of various sizes with sequences of varying length. We found that run times for both programs varied depending on both the number of species in the input phylogeny and the length of the input alignment. However, for the gene tree model, sequence length was the more important factor, with simulated datasets with more than 9 species having roughly the same run times, though this result likely depended on which branches species are added to. We found that for short sequences (100 bp), average run times per locus ranged from 14–46 min depending on the number of species in the phylogeny (fig. 11*A*). However, as sequence length increases, run times also increase substantially. A sequence length of 400 bp, on a tree with 9 species yielded an average run time per locus of 155 min, but a tree with 13 species averaged 460 min (fig. 11*A*). For the species tree model, run times were still correlated with both sequence length and tree size, but are substantially reduced compared to the gene tree model. With the species tree model, average times per locus ranged from just 1.5 s in a tree with 9 species and loci 100 bp to 17 s on a tree with 17 species and sequences 600 bp long (fig. 11*A*). The ratite dataset contains 284,001 noncoding DNA loci with a median length of only 103 bp, meaning that real datasets should be mostly confined to these lower run time estimates (fig. 11*B*). Memory use also scaled with tree size and sequence length, but always remains below 200MB.

As these benchmarks show, the sampling of locus trees implemented in the gene tree model is a computationally intensive process, requiring substantial CPU time to infer substitution rates even for a single locus compared to the species tree model. To address this, we have implemented an adaptive model selection procedure in the user interface that uses site concordance factors (sCF) calculated on each locus to determine whether or not they need to be run with the computationally intensive PhyloAcc-GT, or if the original species tree model in PhyloAcc will suffice. Users provide cutoff values to determine which loci will be run through which model. We show that for the ratite dataset, the average sCF per locus is above 0.5, meaning for most loci, more than 50% of sites support the relationships inferred in the species tree (fig. 11*C*). We varied the average sCF cutoff for these data to see how many loci would be run through PhyloAcc-GT as opposed to the PhyloAcc species tree model and the subsequent effect on estimated run time (assuming linear scaling with increased threads) for the loci that are input to PhyloAcc-GT (fig. 11*D* and *E*). We find that both the number of loci and the estimated run time both increase as the average sCF cutoff is increased, sometimes becoming excessive with run times over 1 year. However, with a low enough cutoff (e.g., below 0.4), we achieve more reasonable run times when only using PhyloAcc-GT on loci with many discordant sites in many branches of the tree.

With the user interface we also provide summary statistics for the input alignments as well as the option to prebatch files for submission to a compute cluster via Snakemake. This batching further reduces run time as batches can be run in parallel.

## Discussion

Detecting complex patterns of substitution rate variation in specific lineages of a phylogeny is an important task that may facilitate the association between small-scale sequence evolution with other biological processes, such as structural variation, habitat or environmental shifts, or even phenotypic evolution (Partha et al. 2019; Smith et al. 2020). However, most tests for rate variation across the tree are usually restricted to protein-coding regions (Yang 1997b; Pond and Muse 2005) and nearly all such methods for detecting such shifts, whether designed for coding or noncoding regions, do not account for ILS and deep coalescence, which can arise in many commonly encountered situations and can induce false signatures of rate variation when ignored (Mendes and Hahn 2016). Here, we present PhyloAcc-GT, which extends PhyloAcc to detect shifts in substitution rate of noncoding loci on phylogenetic trees in the presence of deep coalescence. Through simulation we have shown that accounting for gene tree variation significantly reduces FPRs when detecting rate acceleration on specific branches. PhyloAcc-GT has higher AUPRC than PhyloAcc, especially when the number of conserved loci significantly outnumbers the number of accelerated loci. PhyloAcc-GT is also superior to PhyloAcc and *BEAST2 in identifying patterns of acceleration along a phylogenetic tree and their associated rates. Compared to *BEAST2, PhyloAcc-GT is more confident in identifying all branches in the accelerated state, for both terminal and internal branches. Compared to PhyloAcc, PhyloAcc-GT has better power in identifying internal branches that are in the accelerated state, resulting in more accurate estimation of substitution rates and inference of whether a locus experienced multiple independent accelerations or a single acceleration in an ancestral species. With the introduction of logBF3, which tests support for a model that allows rate acceleration on any lineage, PhyloAcc and PhyloAcc-GT can also be used to test more general hypotheses about molecular evolution in a given phylogeny, such as quantifying which loci are accelerated across the most lineages or which lineages contain the most accelerated loci.

PhyloAcc-GT also provides flexibility in allowing different stationary distributions of DNA substitution models across the genome by inferring the distribution for each locus from the data. Simulations (see supplemantary material S8, Supplementary Material online) show that modeling the stationary distribution of each locus leads to better inference of substitution rates than PhyloAcc, which uses a fixed stationary distribution across all loci and can show poor performance when this global distribution differs significantly from the distribution of a given locus. Here, we have assumed the strand-symmetry model of DNA substitution π; however, the model is easily extendable to other substitution models and priors, such as the Dirichlet distribution. Applying PhyloAcc-GT to accelerated loci in genome-wide bird and mammal datasets, we find that nearly 20% of the loci previously identified by PhyloAcc as accelerated in specific target lineages are likely spurious due to false signatures of acceleration induced by ILS. Thus, for these two datasets, both of which are known to experience ILS, PhyloAcc results in substantial improvements in our ability to identify truly accelerated loci.

An important challenge in considering gene tree variation in the PhyloAcc framework is obtaining parameters of population size θ for each branch of the species tree. Estimating θ for each branch from sequence data or from gene trees is challenging in part because rate variation among loci can mimic variation in coalescence times among loci, sometimes causing identifiability problems (Yang 1997a; Zhu and Yang 2021). Currently, our approach uses separate estimates of branch lengths in substitutions per site (via concatenation) and in coalescent units (via a species tree method such as MP-EST: Liu et al. 2010 or ASTRAL: Mirarab et al. 2014) on a prespecified species tree to obtain estimates of θ, which can therefore vary from branch to branch. This approach likely incurs biases, because, even when working with the same species tree topology, the branch lengths obtained via concatenation are likely mis-estimated and do not precisely correspond to branch lengths in a species tree obtained via coalescent approaches (Edwards 2009; Edwards et al. 2016; Rannala et al. 2020). Additionally, it is well known that methods such as ASTRAL and MP-EST that rely on estimating species tree branch lengths from fixed gene trees estimated in a separate, previous step, result in overestimates of ancestral θ (Yang 1997a, 2002). Still, our analysis of the bird and mammal datasets shows that θs obtained in this manner yields reasonable values of θ, with small differences in θ for most branches, as expected. Additionally, our simulations shows that PhyloAcc-GT is robust to mis-specification of θ when model selection is the focus. However, it can overestimate substitution rates when θs are consistently underestimated, and underestimate them when θs are consistently overestimated. When working with data generated from the null model, using underestimated θs leads to PhyloAcc-GT detecting more false positive cases, while using overestimated θs do not seems to result in more false positive. Adjusting the stringency of model selection via the Bayes Factors will be useful in modulating the FPR in PhyloAcc-GT.

PhyloAcc and PhyloAcc-GT together provide a flexible framework to identify changes in substitution rates along phylogenetic trees with or without deep coalescence. Our current implementation (https://phyloacc.github.io/) also incorporates many improvements in ease of installation (through bioconda) and use. Although the increased model complexity of the gene tree model (PhyloAcc-GT) provides increased accuracy in the presence of ILS, it also incurs increased use of computational resources, sometimes becoming realistically intractable (fig. 11). This naturally comes with the additional cost of higher energy use and a larger carbon footprint when running the more complex model, which is becoming an increasing concern for bioinformatics software developers (Grealey et al. 2022). Considering the tradeoff between the increased accuracy of a more complex model and the increased resource use those models require, it is valuable to develop novel heuristics to guide users to the appropriate method for the given data – in essence not every locus may need to be analyzed with the most complex model. In our case, we developed an adaptive method selection (PhyloAcc vs. PhyloAcc-GT) for different loci within a dataset using site concordance factors (sCF; Minh, Hahn, et al. 2020) to determine the loci that may be most impacted by phylogenetic discordance. By varying the cutoffs for sCF required to run a locus with the PhyloAcc-GT model, we can drastically reduce run time and energy use with minimal impact on analytical results (fig. 11), though some post-hoc analyses may be required to assess rates of error.

Going forward, accurate detection of loci across the genome undergoing rate changes in specific target lineages must eventually grapple with well-known complexities of the genome. For example, our current models assume a single neutral substitution rate across all loci in the genome. However, different regions of the genome likely experience different neutral substitution rates, thereby requiring greater model complexity (Hodgkinson and Eyre-Walker 2011; Eyre-Walker and Eyre-Walker 2014). One way to improve the accuracy of estimation of substitution rates with PhyloAcc might be to use the regions flanking each conserved locus to estimate the local neutral substitution rate for a given locus. Additionally, here we have assumed that all branches in the accelerated rate class share a single substitution rate. This constraint can easily be relaxed to allow independent accelerations on a tree to have different rates. However, we also show in the supplementary material S7, Supplementary Material online that the current PhyloAcc-GT with only three rate values still performs well on data that are generated using more than three rate values. Throughout our manuscript, we assume Dollo’s irreversibility condition such that after an acceleration event occurs on a branch for a given locus, all descendent species remain in the accelerated state. This assumption could be relaxed by allowing for some probability of reverting from an accelerated to a conserved state via the Z matrix; in our software implementation, whether to assume the Dollo model is a user-specified option.

PhyloAcc and PhyloAcc-GT currently focus on conserved noncoding loci that use standard models of nucleotide substitution. Arguably, the much large number of conserved noncoding loci than genes or exons in genomes and their likely widespread role in driving phenotypic evolution make a focus on noncoding variation a profitable place to start (Mattick 2005; Marcovitz et al. 2016; Lewis et al. 2019; Sackton et al. 2019). However, we can extend this model to detect rate shifts in protein-coding regions as well. Finally, for $M_{1}$, PhyloAcc, and PhyloAcc-GT currently focus on sets of target lineages that are in or not in a designated target set or are characterized by a binary trait. We have relatively few models that explicitly model associations of genomic substitution rates with continuous phenotypes (Kowalczyk et al. 2019, 2020, 2022; Lartillot and Poujol 2010). Such continuous phenotypes likely better characterize many traits, and may provide additional power to link genotype and phenotype via phylogenetic trees.

## Supplementary Material

msad195_Supplementary_Data

## Data Availability

PhyloAcc and PhyloAcc-GT are open source software under the GNU General Public License (v3.0) and are freely available at https://phyloacc.github.io/. All input and output files for the analysis of the simulated data, ratite data, and mammal data as well as the scripts used to generate the figures in this manuscript are also available at https://github.com/phyloacc/Yan-etal-2022, with the exception of nucleotide alignments. These are available in the original PhyloAcc paper (Hu et al. 2019).
