# Supplementary material for: PhyloAcc-GT: A Bayesian Method for Inferring Patterns of Substitution Rate Shifts on Targeted Lineages Accounting for Gene Tree Discordance
Source: Mol Biol Evol. 2023 Sep 4;40(9):msad195. doi: 10.1093/molbev/msad195 (PMC10540510; doi:10.1093/molbev/msad195)
Supplement: msad195_Supplementary_Data [file msad195_supplementary_data.pdf]

S1 Gene tree proposal algorithm

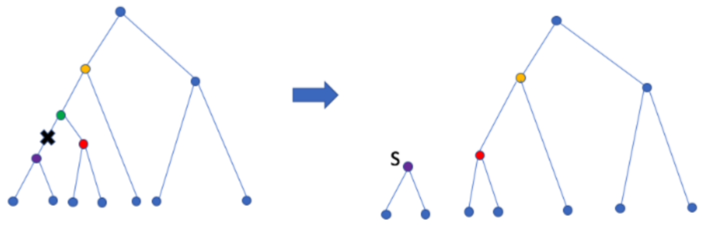

(a) Step 1: an internal branch is selected and dissolved. This results in two subtrees.

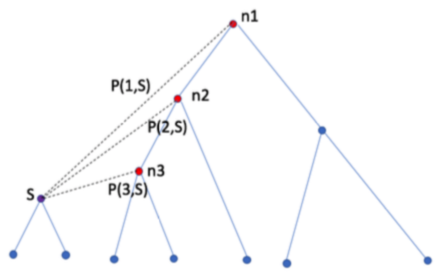

(b) Step 2 and 3: identify all potential grand-parent nodes of node s, and calculate the transition probability from each candidate node to s.

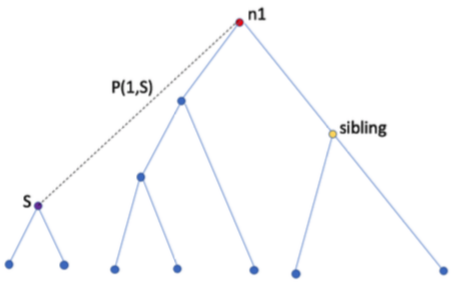

(c) Step 4 and 5: sample a candidate node, e.g., n1, to be the new grand-parent node of s, and sample a sibling node for s from the two child nodes of n1.

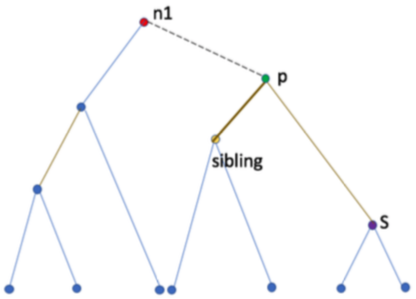

(d) Step 6: sample a position along the sibling branch to be the proposed position of the new parent node p of s and the sibling node.

Figure S1: The proposal algorithm used when sampling a distribution of gene trees while estimating rates for a locus.

S2 Species trees for ratites and mammals

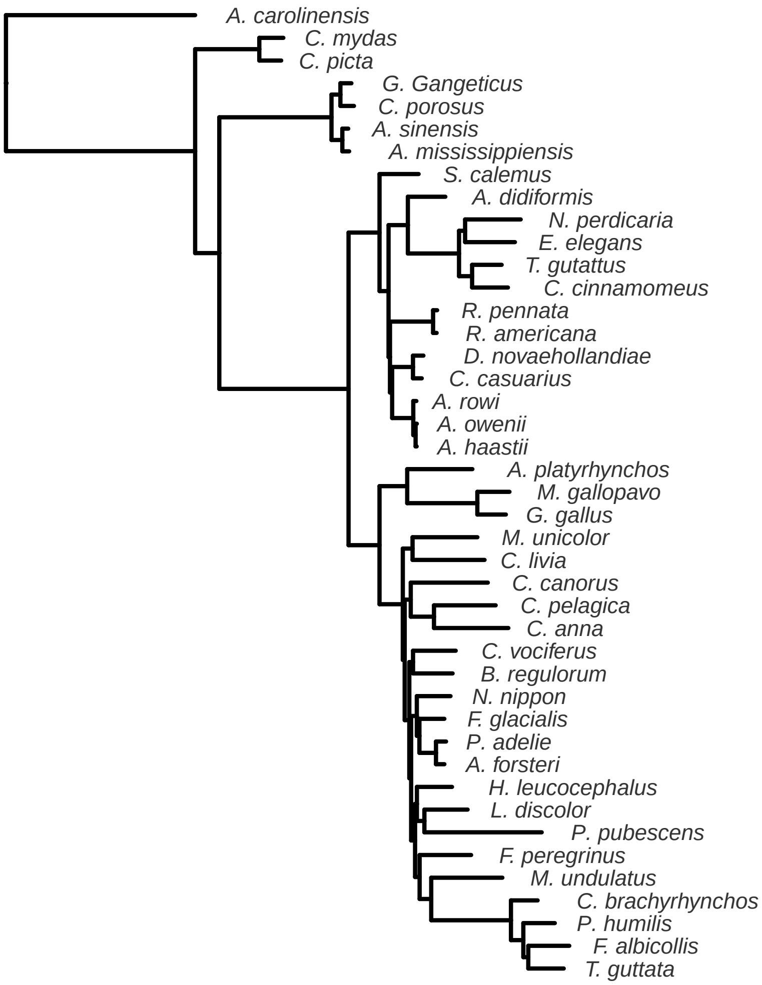

Figure S2: The full avian phylogeny used in this study, from Sackton et al. (2019) and Hu et al. (2019).

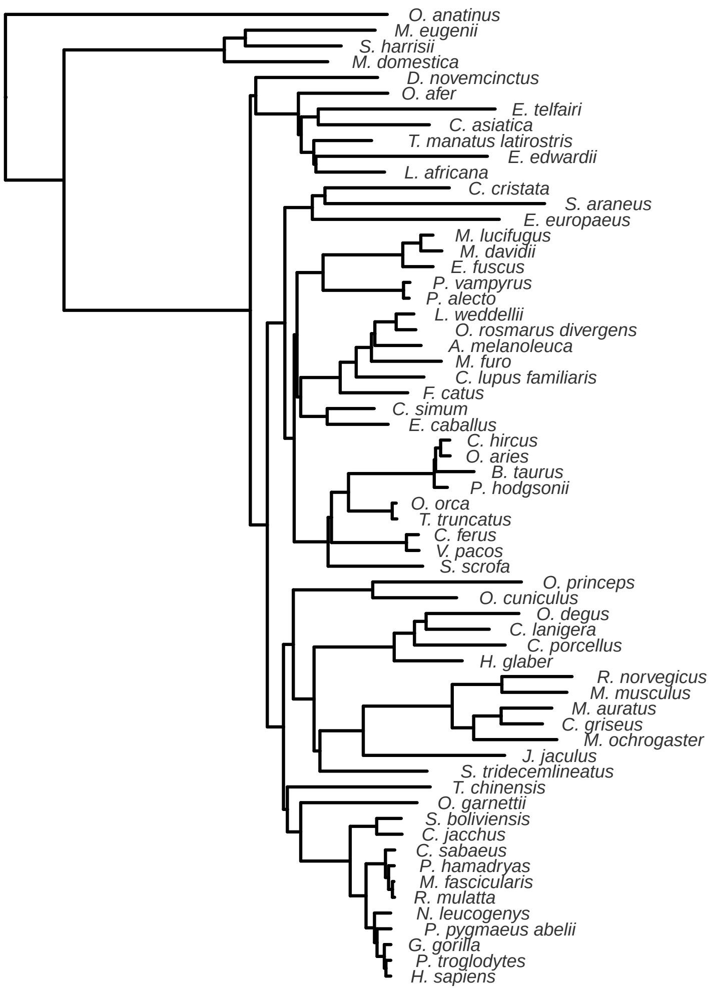

Figure S3: The full mammal phylogeny used in this study, from the UCSC 100-way vertebrate alignment Blanchette et al. (2004) and Hu et al. (2019).

S3 Correctness of the proposed MCMC algorithm for gene tree inference

Case 1: Inferring branch length on a 2-leaf tree

To check the performance of the proposed MCMC algorithm for estimation of gene tree branch lengths, we first examine the simplest case: a 2-leaf tree. In this case, the two

lineages only coalesce in the root species. There is no node above the root node on the gene tree to constrain our sampling. We set a hard threshold: $10 \times \frac{\theta_{root}}{2}$ as the maximum height of the gene tree. Inferring the coalescent time is the same as inferring the position of the gene tree root. The Metropolis algorithm uses the uniform distribution centered at the current root node position as the proposal distribution. The step size is set as $\frac{\theta}{2} \times \delta$, where $\delta \in [0.1, 5]$ is adaptive to ensure a reasonable acceptance rate. When the acceptance rate is too high, we will scale δ by a factor of 2; if the acceptance rate is too low, we will scale down δ to $\frac{\delta}{2}$. The proposal distribution is also constrained by the species tree and the upper limit of gene tree height that we set.

In this simple case, we can estimate some statistics of the posterior distribution, e.g., the posterior mean of the branch length, l, using numerical integration.

$$E[l \mid Y] = \int lf(l \mid Y)dl \approx \sum_i l_i f(l_i \mid Y)\Delta_l = \frac{\sum_i l_i f(Y \mid l_i) f(l_i)\Delta_l}{\sum_i f(Y \mid l_i) f(l_i)\Delta_l}$$

Case 2 - Inferring gene tree topology and branch lengths on a 3-leaf tree

To estimate the posterior probability of a gene tree topology, using Bayes' theorem, we can write the posterior probability as:

$$P(G_{top} \mid Y) = \frac{P(Y \mid G_{top})P(G_{top})}{P(Y)} \tag{S1}$$

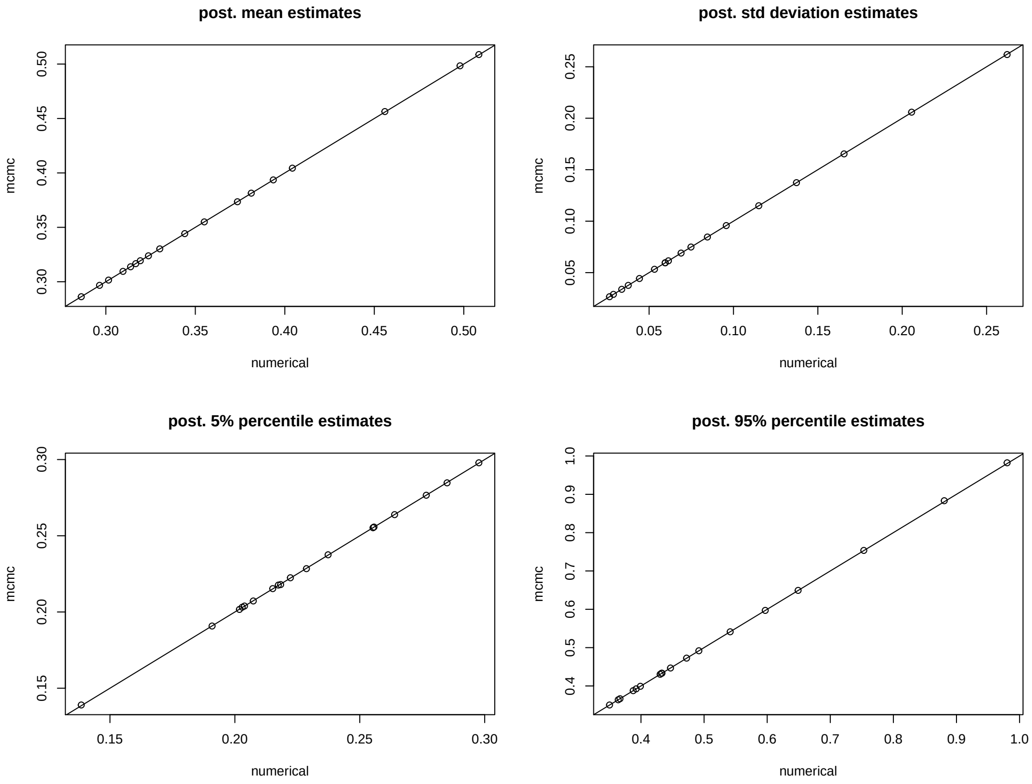

Figure S4: Comparison between our MCMC algorithm and numerical integration on some summary statistics of posterior distributions of coalescent time in the 2-leaf tree case. We ran the experiment under different number of base pairs ranging from 50 to 5000. We estimated posterior mean, standard deviation, tail probabilities: i.e. 5% quantile and 95% quantile using both MCMC sampling output and numerical integration. The x-axis represents results using numerical integration and the y-axis corresponds to the MCMC output. The line in each plot is $y = x$. For all four statistics, estimation results using the two methods fall almost perfectly along the $y = x$ line.

We estimate $P(\boldsymbol{Y})$ by $P(\boldsymbol{Y}) = E_G[P(\boldsymbol{Y} \mid G)] \approx \frac{1}{N} \sum_{i=1}^{N} P(Y \mid G_i)$, where a large number

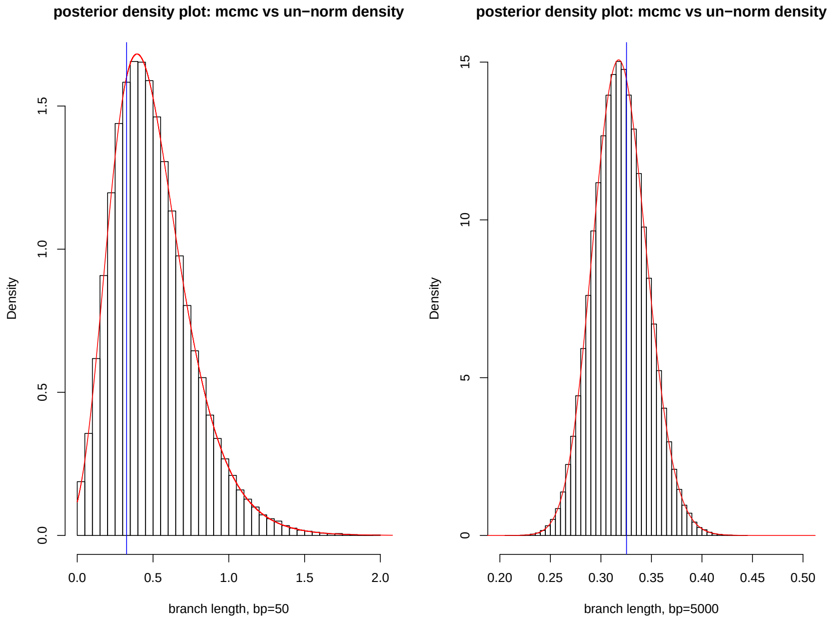

Figure S5: Posterior density plots in the 2-leaf tree case. The left plot corresponds to the posterior distribution of coalescent time with 50 base pairs, and the right plot is for 5000 base pairs. Histograms are based on MCMC sampling output. Red curves are plotted based on un-normalized posterior densities on grid points. Blue vertical lines are the true coalescent time. The red curves and histograms align very well, indicating our MCMC algorithm is sampling from the targeted posterior distributions. As the number of base pairs increases from 50 to 5000, the posterior distribution becomes more concentrated around the true coalescent time.

N of gene trees are simulated from its prior distribution. $P(Y \mid G_{top}) = \int P(Y \mid G)P(G \mid G_{top})dG \approx \frac{1}{N_1}\sum_{i=1}^{i=N_1} P(Y \mid G_i)$, where $G_i, i = 1, \cdots, N_1$ are N_1 prior trees with the sample topology G_{top}. $P(G_{top})$ can be estimated by the sampling proportion of the N prior trees with the particular topology denoted by G_{top}. For a 3-leaf tree, the prior probability of each

gene tree topology can be analytically calculated by integrating out all branch lengths. Let G_1 denote the gene tree topology that is the same as the species tree (T) topology, and let G_2 and G_3 be the remaining two gene tree topologies.

$$P(G_1 \mid T, \Theta) = 1 - \frac{2}{3}exp(-\frac{2}{\theta_1}l_1)$$
$$P(G_2 \mid T, \Theta) = P(G_3 \mid T, \Theta) = \frac{1}{3}exp(-\frac{2}{\theta_1}l_1)$$

where l_1 is the branch length on the species tree from the root to the first speciation event, and θ_1 is the population size parameter of the species before the first speciation event. So Equation S1 can be approximated by:

$$P(G_{top} \mid Y) \approx \frac{\frac{1}{N_1}\sum_{i=1}^{i=N_1} P(Y \mid G_i)P(G_{top})}{\frac{1}{N}\sum_{i=1}^{N} P(Y \mid G_i)} \tag{S2}$$

We also checked the correctness of our MCMC algorithm in inferring coalescent time under the 3-leaf tree case. The results are summarized in Figure S7 and S8.

S4 Correctness of PhyloAcc-GT's sampling algorithm for gene tree prior distribution

In PhyloAcc-GT we have implemented an algorithm to sample gene trees from their prior distribution conditioning on a species tree according to the multispeices coalescent model (Rannala and Yang, 2003). We use a simulation study to show that our sampling algorithm

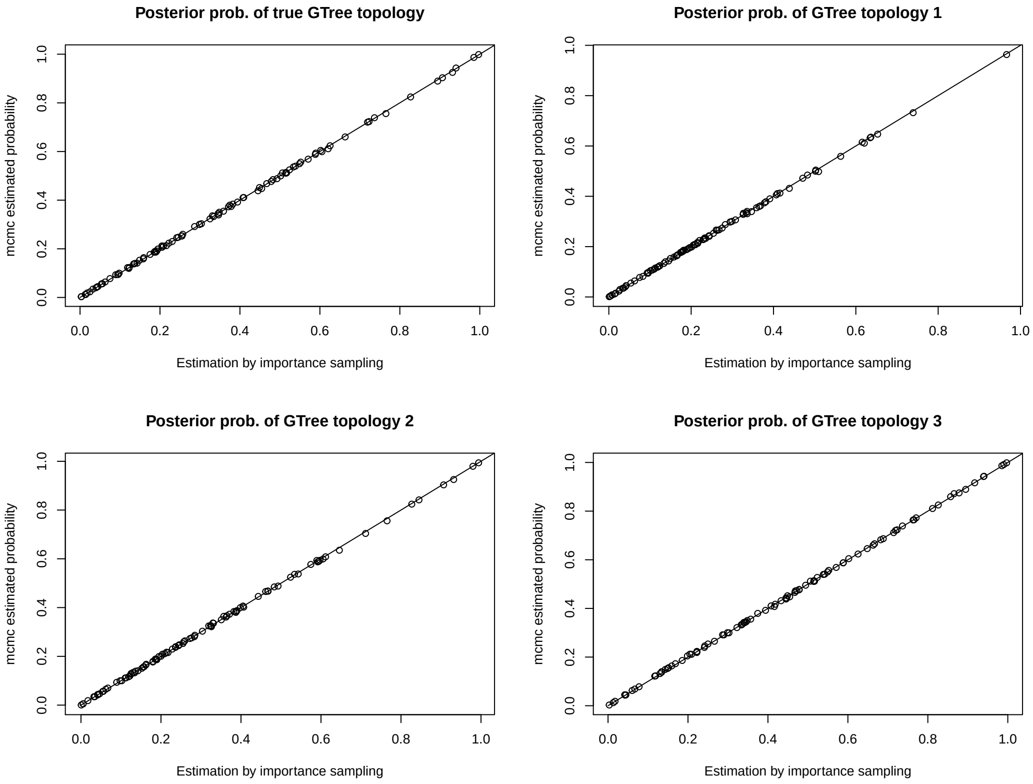

Figure S6: Estimating posterior probabilities of different gene tree topologies in the 3-leaf tree case. The x-axis corresponds to estimation using Equation S2. The y-axis corresponds to the estimation using sampling frequencies of our MCMC algorithm. We generated 100 loci: i.e. 100 gene trees and base pairs based on each gene tree. The dots represent the estimation results for the 100 loci. The top-left plot compares estimation results for the posterior probability of the true gene tree topology. The remaining three plots are estimation results for the posterior probabilities of each of the three possible gene tree topologies. In all cases, the estimations by MCMC sampling frequency and by Equation S2 are well aligned along the $y = x$ line, indicating the correctness of our algorithm.

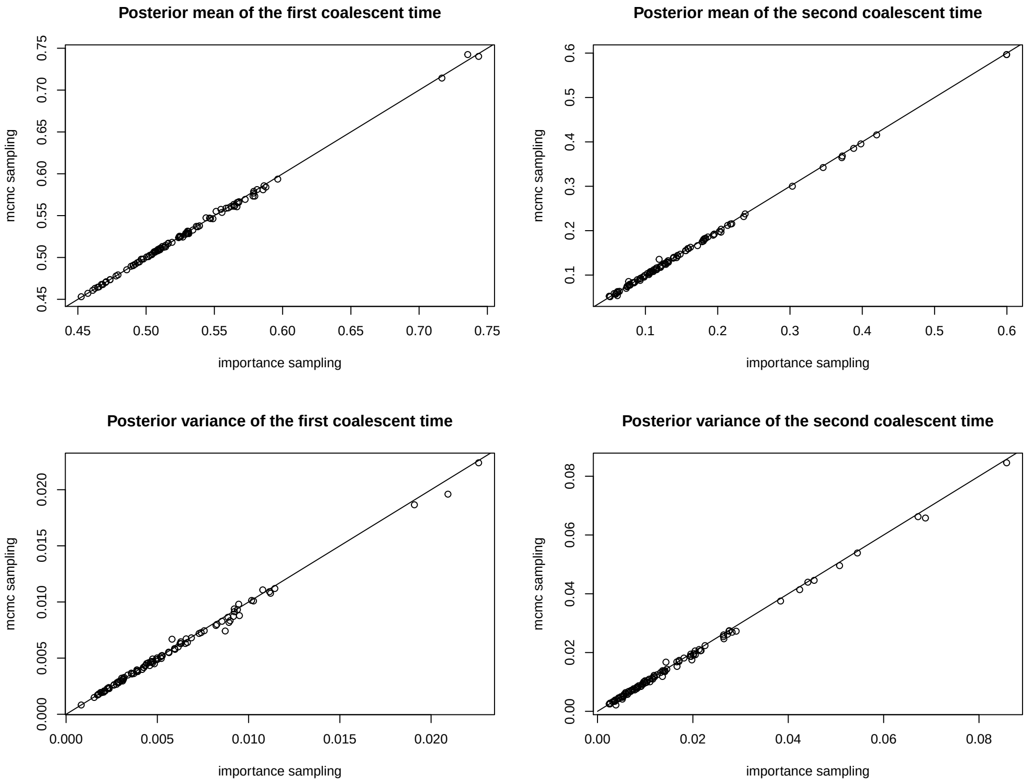

Figure S7: Correctness of the MCMC algorithm in inferring the posterior mean and variance of the two coalescent times in the 3-leaf tree case. Left plots correspond to the posterior mean and variance of the first coalescent time, and the plots on the right correspond to the second coalescent time. The x-axis corresponds to approximating the estimate by sampling branch lengths from the conditional prior distributions and approximating expectations by sample averages, similar to Equation (S2). The two estimation methods are very close to each other.

is correct. We show that several characteristics of the sampled gene trees match those of gene trees sampled from Phybase (Liu and Yu, 2010) in R given the sample species tree. We fix the species tree as: "$((A : 0.05\#0.01, B : 0.05\#0.01) : 0.05\#0.08, ((C : 0.06\#0.01, D :$

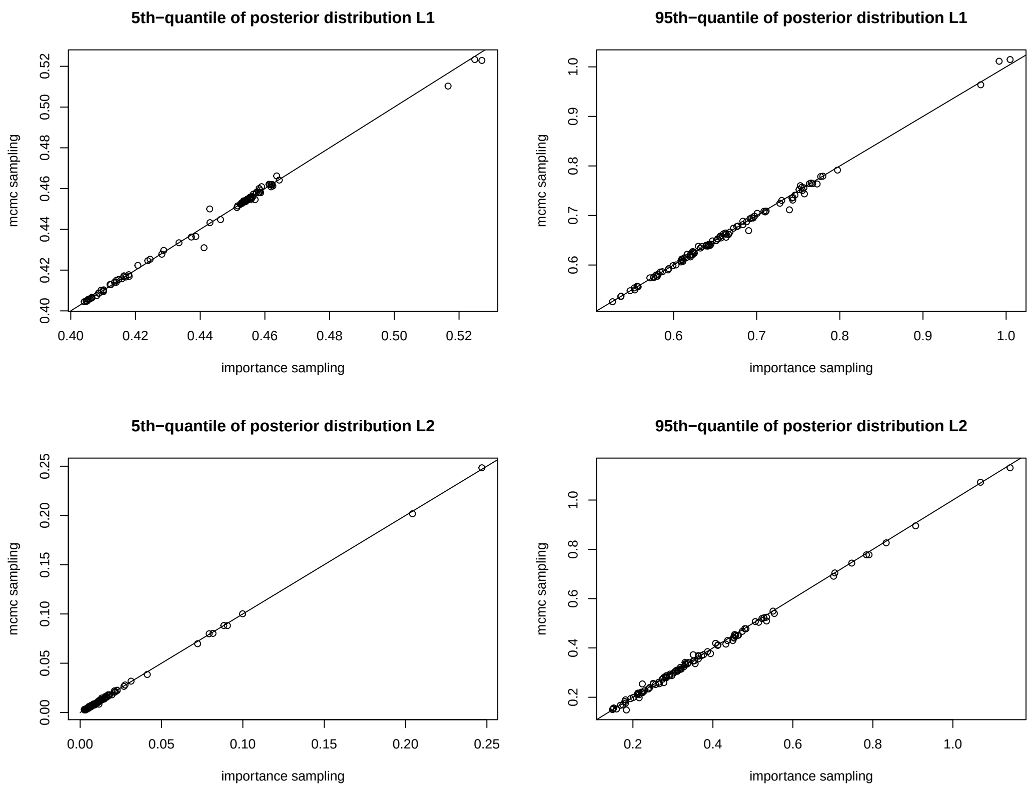

Figure S8: Correctness of the MCMC algorithm in estimating tail probabilities, i.e. 5% and 95% quantiles of the posterior distributions of the two coalescent times in the 3-leaf tree case. Upper plots are for the first coalescent time, and lower plots are for the second coalescent time. The two estimation methods gives very similar results, indicating the correctness of our MCMC algorithm.

0.06#0.01) : 0.02#0.06, E : 0.08#0.01) : 0.02#0.05)#0.02;", with topology plotted in Figure S9.

For each leaf node, we plot the histogram of its branch length. See Figure S10.

We also plot the histograms of the branch lengths of internal nodes of the most sampled gene tree topology in Figure S11.

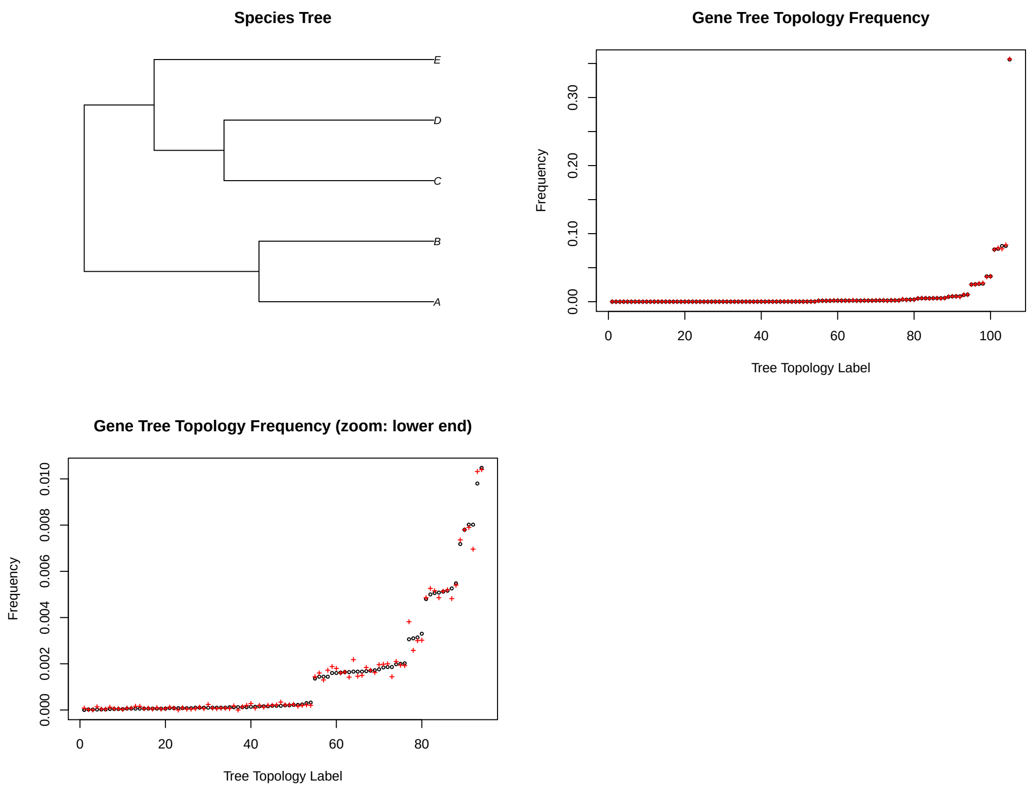

Figure S9: The species tree topology for simulation study in Section S4, and gene tree sampling frequencies using PhyloAcc-GT and Phybase. Total number of samples is 50,000. The top right figure shows that sampling frequencies at various tree topology using PhyloAcc-GT (black dots) and Phybase (red plues) are very close to each other. The bottom left figure focuses on the low frequency end of the top right figure. With five extant species, the total number of rooted gene tree topologies is 105. The most frequently sampled gene tree topology matches the species tree topology in both algorithms. The corresponding frequencies are around 35%.

Lastly we plot the histograms of some most frequently sampled internal nodes in Figure S12.

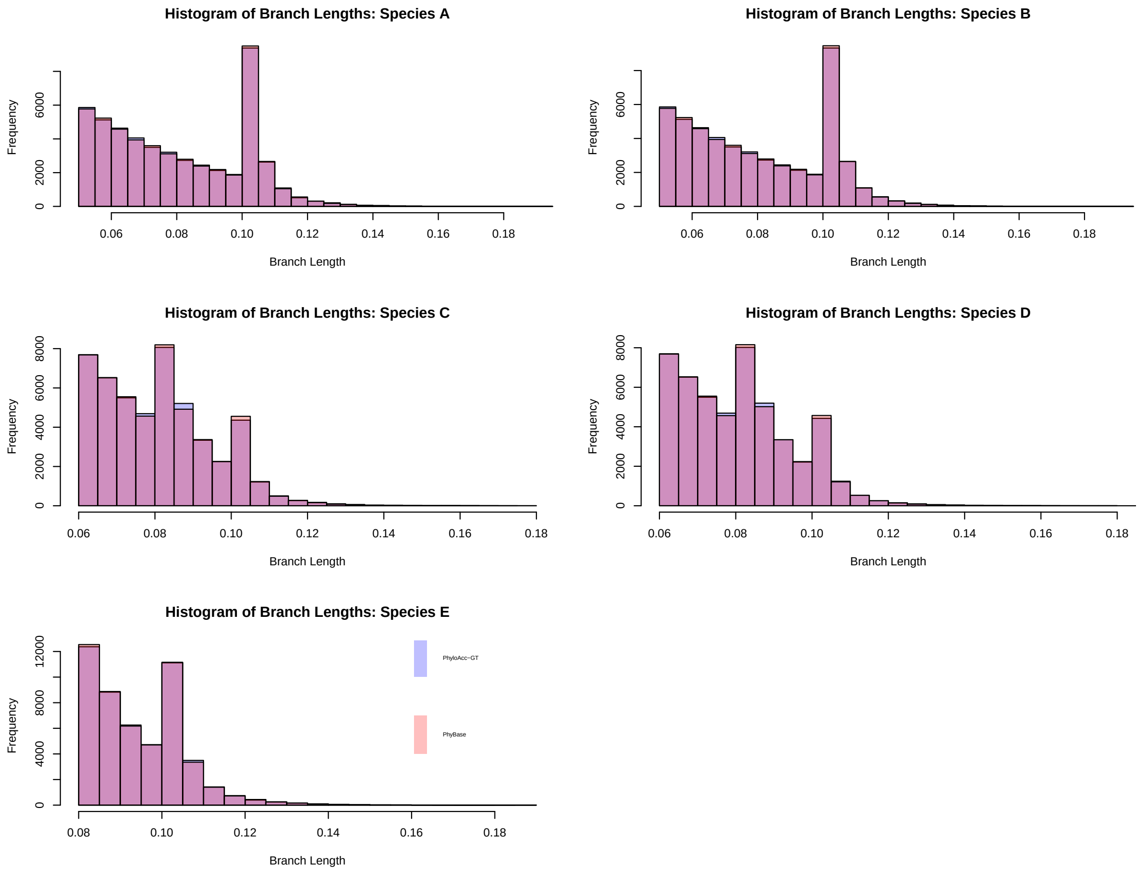

Figure S10: Overlapping histograms of sampled branch lengths of leaf nodes under PhyloAcc-GT and Phybase. For all 5 extant species, the sampling distributions of their branch lengths are very similar between PhyloAcc-GT and Phybase. The sampling distributions of the branch length of species A and the branch length of species B are very similar. The sampling distributions of the branch length of species C and the branch length of species D are also very similar. This result arises because in our samples, the genes in species A and B are most likely to coalesce first before coalescing with other lineages. A similar situation occurs with species C and D.

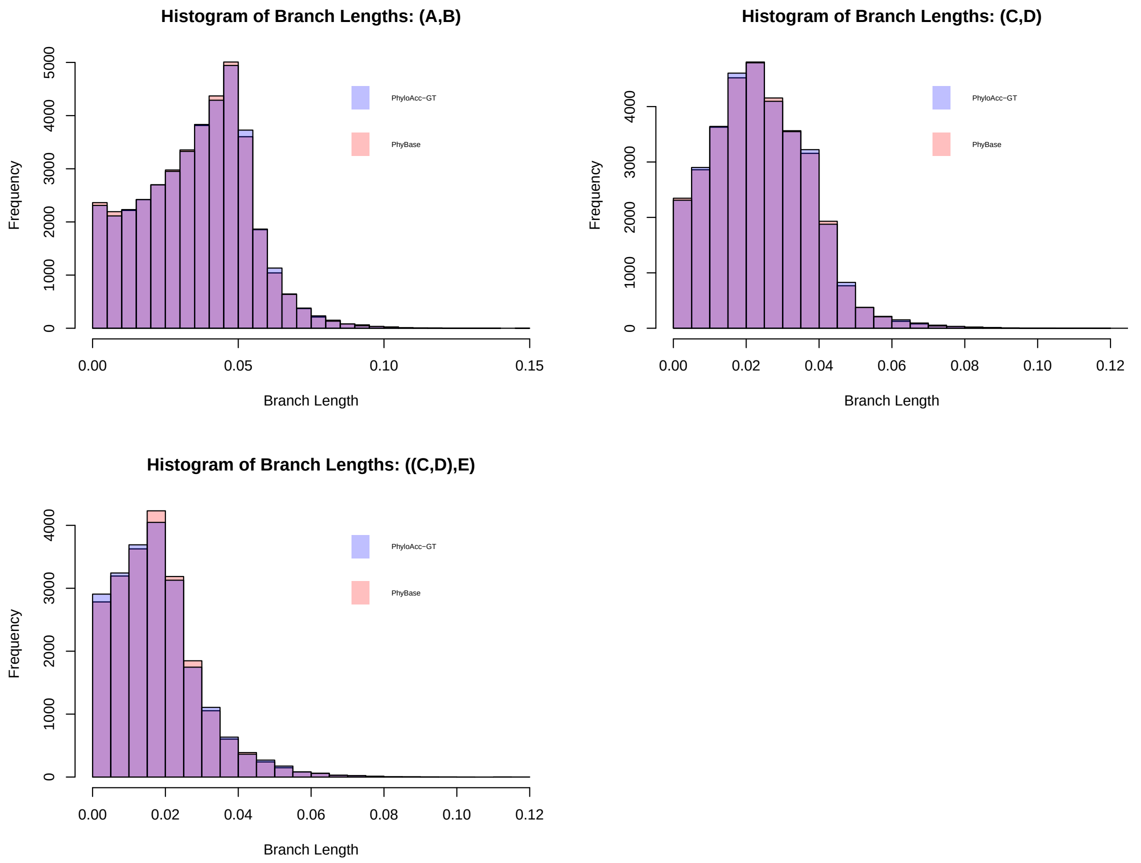

Figure S11: Overlapping histograms of some of the most frequently sampled internal nodes. The sampling distributions are similar between PhyloAcc-GT and Phybase.

S5 Analyzing estimated rates

For PhyloAcc-GT and PhyloAcc, we also compare their estimated conserved rate and non-conserved rate under different patterns of acceleration. The result is shown in Figure S13. For all cases, rates estimated by PhyloAcc-GT have higher correlations with the underlying true rates than rates estimated by PhyloAcc. PhyloAcc tends to overestimate rates, especially for the non-conserved rates, as can be seen in Figure S14, S15 and S16. The overestimation

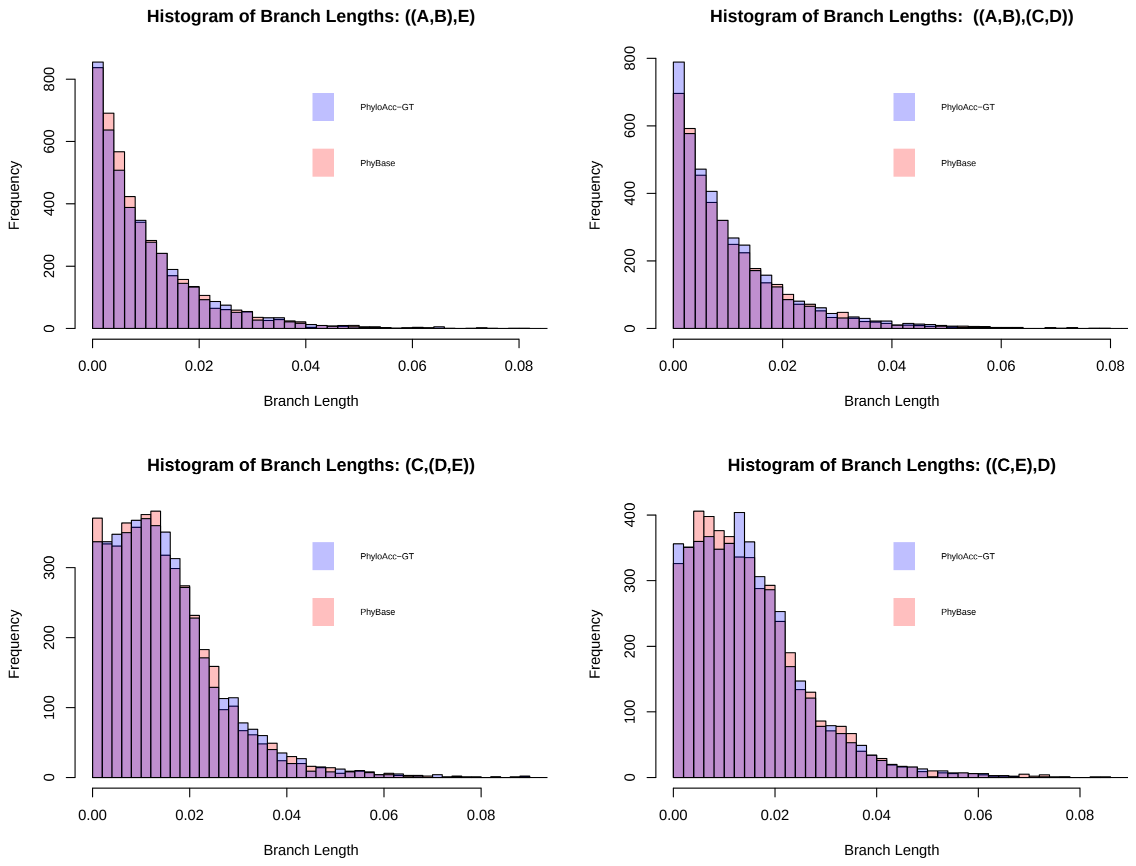

Figure S12: Overlapping histograms of some of the most frequently sampled internal nodes. The sampling distributions are similar between PhyloAcc-GT and Phybase.

is caused by ignoring gene tree heterogeneity due to incomplete lineage sorting, as well as the stationary distributions of nucleotide frequencies.

Our algorithm also gives good estimates of the frequencies of different nucleotides in the stationary distribution. We use the posterior mode as its point estimate. Plots of the estimated versus the true frequency of adenine in the stationary distribution are shown in Figure S17 for two independent accelerations (Figure 2C). Relationships are very similar for the cases of a single acceleration (Figure 2B) and three independent accelerations (Figure

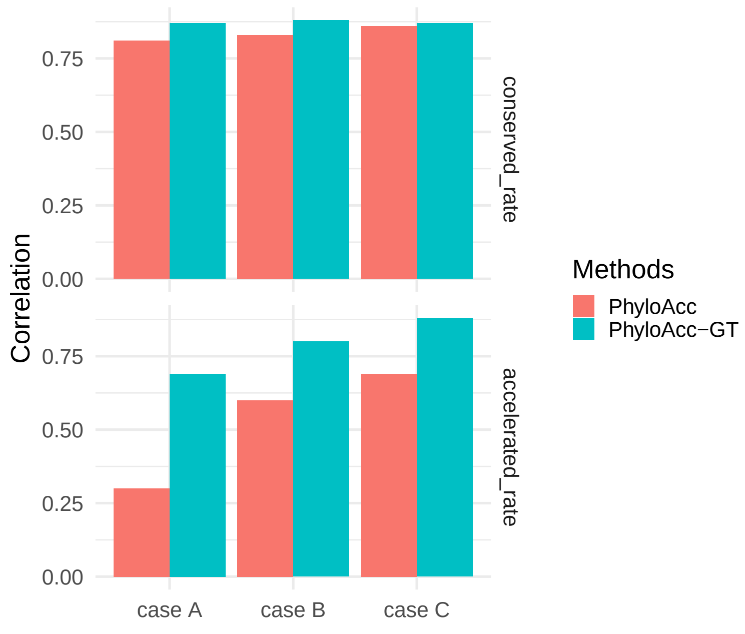

Figure S13: Comparing correlations between true rates and estimated ones by PhyloAcc-GT and PhyloAcc.

2D), hence they are omitted. The correlations between the two are 0.927, 0.9 and 0.935 in the three simulation cases Figure 2. Regressing the estimated π_A against the true π_A without an intercept term, the regression coefficient for two independent accelerations (Figure 2C) is 1.001, and 0.989 and 0.986 for a single acceleration (Figure 2B) and three independent accelerations (Figure 2D) respectively.

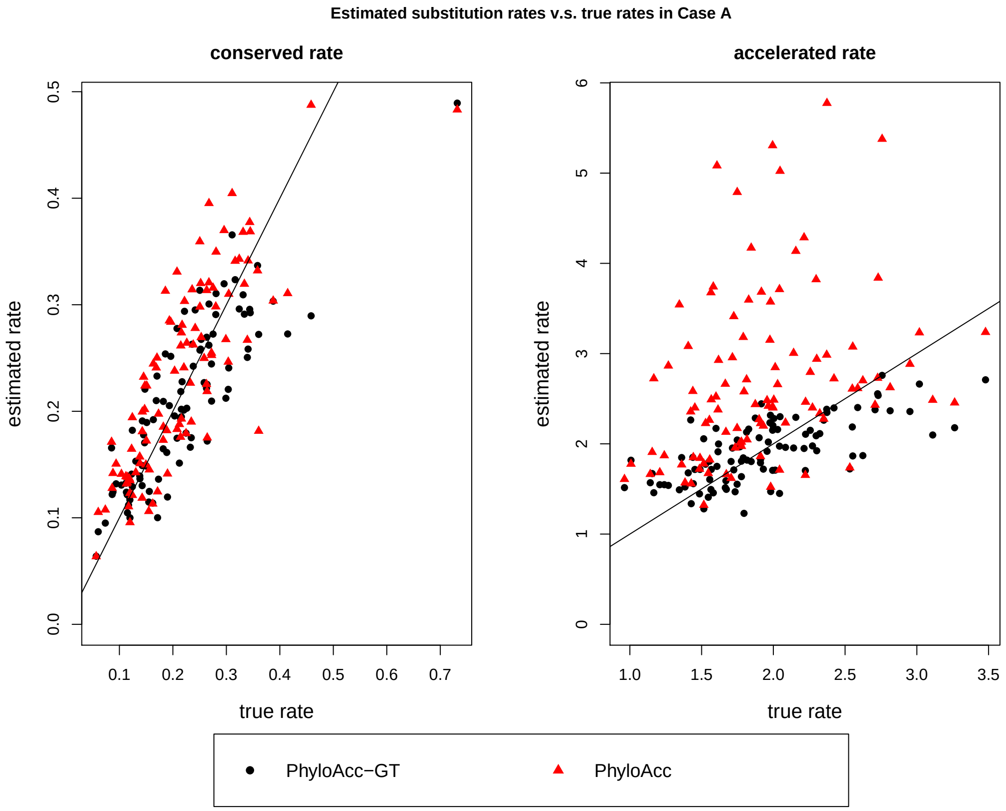

Figure S14: Comparing estimation of conserved and non-conserved rates by PhyloAcc-GT and PhyloAcc with sequences simulated with a single acceleration (Figure 2B). The line is Y=X.

S6 Details on analyzing *BEAST2 results and more investigations

S6.1 Identifying accelerated branches

As *BEAST2 does not explicitly calculate the probability of acceleration for each lineage, we use the following method to estimate $P(Z = 2 \mid \boldsymbol{Y})$ from *BEAST2 in order to compare

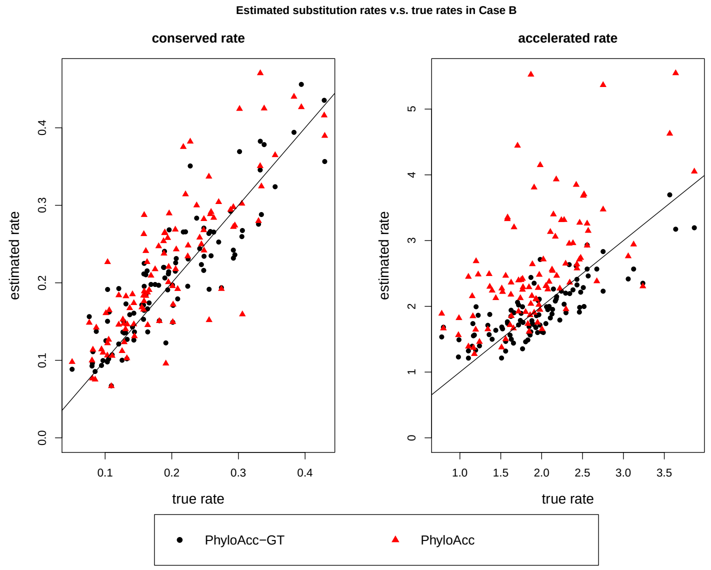

Figure S15: Comparing estimation of conserved and non-conserved rates by PhyloAcc-GT and PhyloAcc with sequences simulated with two independent accelerations Figure 2C). The line is Y=X.

with PhyloAcc-GT and PhyloAcc.

For a locus, we output posterior samples of substitution rates on all species tree branches from MCMC (after thinning at 1000, and discarding the first 25% MCMC steps as burn-in). In each MCMC step (m), we compare the substitution rate on a branch (s), denoted as r_s^m to the substitution rate on its parent branch $(r_{pa(s)}^m)$. If $r_s^m > r_{pa(s)}^m$ or if $r_s^m = r_{pa(s)}^m$ and branch $pa(s)$ is accelerated, we treat branch s as accelerated. We estimate $P(Z_s = 2 \mid \boldsymbol{Y})$ as the proportion of times branch s is treated as accelerated out of all the MCMC runs.

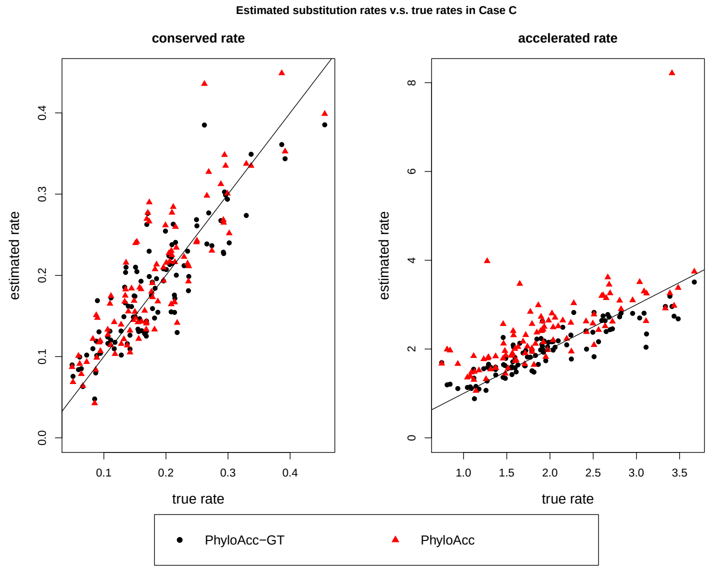

Figure S16: Comparing estimation of conserved and non-conserved rates by PhyloAcc-GT and PhyloAcc with sequences simulated with three independent accelerations (Figure 2D). The line is Y=X.

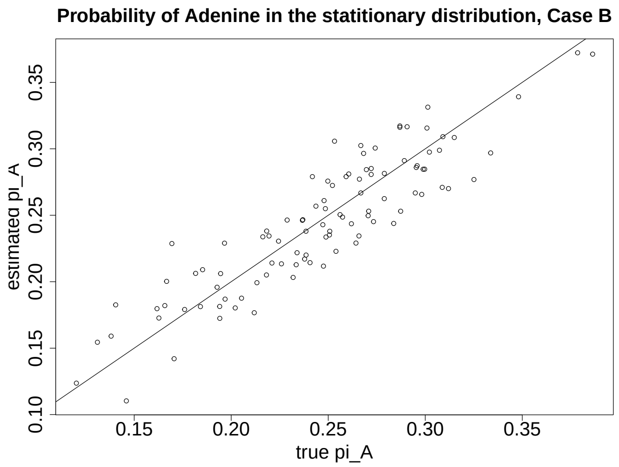

Figure S17: Estimated stationary probability of adenine v.s., the true probability with sequences simulated with a single acceleration (Figure 2B).

S6.2 Identifying accelerated branches based on other criteria

In this section, we explore the performance of *BEAST2 in identifying accelerated branches using different criteria.

For each branch s, we record all of its posterior samples of substitution rates. As a point estimate $\hat{r}_s$, we use both 1) posterior mean, and 2) posterior median. We also set different thresholds when determining whether a branch is accelerated: a) $\hat{r}_s > \hat{r}_{pa(s)}$, b) $\hat{r}_s > \hat{r}_{pa(s)} + \hat{\sigma}_{pa(s)}$, and c) $\hat{r}_s > \hat{r}_{pa(s)} + 2\hat{\sigma}_{pa(s)}$. $\hat{\sigma}_s$ is the sample standard deviation of the substitution rate on branch s.

Results on average probability of acceleration in each branch of the tree under different acceleration patterns and θ specifications are plotted below. Each bar represent a probability of acceleration for a branch averaged over results from 100 loci calculated under an alternative criterion:

1. "mean, 0 threshold": Acceleration is determined by comparing the posterior mean of a branch's substitution rate to its parent's mean rate.

2. "median, 0 threshold": Acceleration is determined by comparing the posterior median of a branch's substitution rate to its parent's median rate.

3. "median, median as threshold": We first estimate each branch's substitution rate by its posterior median. We take the median of all branches' estimated substitution rates, denoted as $\bar{\hat{r}}$, as a threshold for acceleration. If a branch's estimated rate is greater than $\bar{\hat{r}}$, we treat this branch as accelerated, otherwise, the branch is not accelerated.

4. "median, std as threshold": We use the same approach as the previous one (point 3), i.e., using posterior median as the point estimate of substitution rate $\hat{r}_s$. However, we have more strict rule in selecting a branch as accelerated. For each branch, we also calculate the sample standard deviation of the posterior distribution of its substitution rate $\hat{\sigma}_s$. A branch is considered accelerated if $\hat{r}_s > \hat{r}_{pa(s)} + \hat{\sigma}_{pa(s)}$.

5. "median, 2std as threshold": similar to the previous one (point 4), but we require a branch's rate to satisfy $\hat{r}_s > \hat{r}_{pa(s)} + 2\hat{\sigma}_{pa(s)}$ in order to treat it as an accelerated branch.

We observe that the first two criteria produces similar results. They tend to give smaller probabilities of acceleration than the later three criteria. Using the first two criteria, most branches in all acceleration cases tend to have low probabilities of acceleration (below 0.5). They fail to identify accelerated branches in most scenarios. The remaining three criteria tend to give much higher probabilities of accelerations across branches. As a result, they have higher false positive rates (non-accelerated branches are falsely identified as accelerated).

S7 Additional simulations with different priors for substitution rates

S7.1 Simulation result: decreasing r_2/r_1 ratio

The ability of PhyloAcc-GT to choose the correct model, and to correctly identify accelerated branches, depends on the relative magnitude of the accelerated rate compared to

the conserved rate. In the manuscript, we fixed the ratio of the mean of r_2 to the mean of r_1 to be $2/0.2 = 10$. In this section, we gradually decrease the ratio of the means, and analyze PhyloAcc-GT's performance. In all simulations, we fix the prior for r_2 to be $r_2 \sim Gamma(40, 0.5)$, with a mean rate of 2, while changing the priors of r_1 from $Gamma(300, 0.001)$, $Gamma(400, 0.001)$, $\cdots$, $Gamma(600, 0.001)$. These Gamma distributions correspond to rates with means equal to 0.3, 0.4, 0.5, and 0.6. We generate 100 loci for each combination of r_1 and acceleration pattern.

Fig. S24 to Fig. S26 show boxplots of log Bayes Factors as r_1 gets closer to r_2 under different acceleration patterns. For both logBF1 ad logBF2, we observe decreasing trends as r_1 increases towards r_2. At larger r_1s, there are a few loci having negative logBF1, i.e., favoring the null model over the restricted model when there are one or two independent accelerations on the tree. As r_1 gets larger, it becomes harder to distinguish the conserved and the non-conserved rate. In the two scenarios where there are one or two independent accelerations, the total number of branches in the accelerated state are much fewer than the total number of branches in the non-accelerated state, hence making it harder to accurately infer the non-conserved rates, and distinguish them from the conserved rates. There are also a few loci favoring the full model over the restricted model, as r_1 increases. However, for most loci, both logBF1 and logBF2 are significantly greater than 0. Even when r_1s are around 0.6, medians of logBF1 are all above 5 in all three acceleration patterns, and medians of logBF2 are above 4.

We also plot $P(Z = 2 \mid \boldsymbol{Y})$ for all branches on the tree for each r_1 and acceleration

pattern combination. Identifying accelerated branches is relatively robust to increases in r_1. We observe a slight increase in the variance of the $P(Z = 2 \mid \boldsymbol{Y})$ on internal branches in the accelerated state (e.g., (A1,B1) in the case one independent acceleration, and (C1,C2) in the case two independent accelerations). However, for most loci, $P(Z = 2 \mid \boldsymbol{Y})$s for accelerated branches are above 0.5, and for the majority of loci, the posterior probability of acceleration is above 0.75. Variation of $P(Z = 2 \mid \boldsymbol{Y})$ increases for tip lineages D1 and F1 under the three independent acceleation case. For each lineage, D1 or F1 is the only branch that is accelerated, limiting the amount of information used to infer its substitution rate, and distinguish the accelerated rate from the conserved rate. As for non-accelerated branches, more than 95% of the time, their posterior probabilities of acceleration are at or nearly 0 even when r_1 has a mean of 0.6.

Lastly, comparing AUPRC curves between results by PhyloAcc-GT and by PhyloAcc supports that PhyloAcc-GT's model performance is increasingly better than PhyloAcc as the ratio of mean conserve rate to mean non-conserved rate decreases.

S7.2 Simulation result: two accelerated rates

We assume in PhyloAcc and PhyloAcc-GT that there are only three rate categories. All branches in the accelerated state share the same non-conserved rate r_2. In reality, this assumption may not hold. Without changing our model specifications, we analyze how PhyloAcc-GT performs when sequences are simulated from four rate categories. Specifically, we change our prior on r_2 from Gamma(10,0.2) to 0.5*Gamma(10,0.2)+0.5*Gamma(15,0.2).

When simulating sequences, we first sample two non-conserved rates $r_2^1 \sim Gamma(10, 0.2)$ and $r_2^2 \sim Gamma(15, 0.2)$ For each accelerated branch, we randomly choose its substitution rate from r_2^1 and r_2^2. Other parts of the sequence generating process are not changed. For each acceleration pattern (1, 2 or 3 independent accelerations on the tree), we simulate 100 loci, and run PhyloAcc-GT.

Under the case one independent acceleration, PhyloAcc-GT prefers the correct model (the restricted model) in 98% of the loci. While the model selection accuracy is 100% under the other two acceleration patterns. Fig. S34 show boxplots of $P(Z = 2 \mid \boldsymbol{Y})$ for all branches on the tree under each acceleration pattern. The result is similar to that obtained when we simulate with only three rate categories. In all acceleration patterns, $P(Z = 2 \mid \boldsymbol{Y})$s are close to 1 for accelerated branches. $P(Z = 2 \mid \boldsymbol{Y})$s are at 0 for all non-accelerated branches except for 2 loci under case one independent acceleration, but all posterior probabilities of accelerations are below 0.5.

S7.3 Simulation result: every accelerated branch has an unique accelerated rate

In this subsection, we go one step further from the previous scenario. When generating sequences, for each accelerated branch, we generate an accelerated rate unique to this branch from a two-gamma mixture: 0.5*Gamma(10,0.2)+0.5*Gamma(15,0.2). This greatly increases the variation in accelerated rates, and becomes harder to identify accelerated branches. As PhyloAcc-GT only assumes one accelerated rate, we think of PhyloAcc-GT as

trying to estimate an average accelerated rate.

PhyloAcc-GT still correctly identify the restricted model as the best model in most cases. Under the case one, two, or three independent accelerations, PhyloAcc-GT selects the correct model (restricted model) 100%, 98%, and 99% of times. When estimating $P(Z = 2 \mid \boldsymbol{Y})$, PhyloAcc-GT performs similarly as in the previous subsection when there are two accelerated rates. For a majority of the loci and accelerated branches, $P(Z = 2 \mid \boldsymbol{Y}) > 0.75$. However, when estimating $P(Z = 2 \mid \boldsymbol{Y})$ for non-accelerated branches, there are three loci (1% of all loci) that are falsely identified as accelerated in some non-accelerated branches.

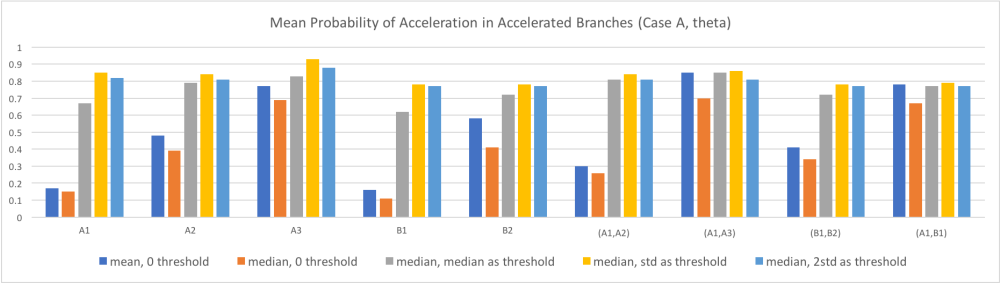

(a) Case A, θ

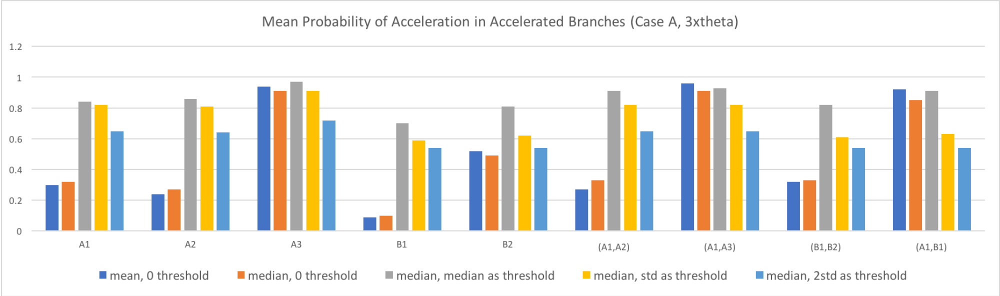

(b) Case A, 3θ

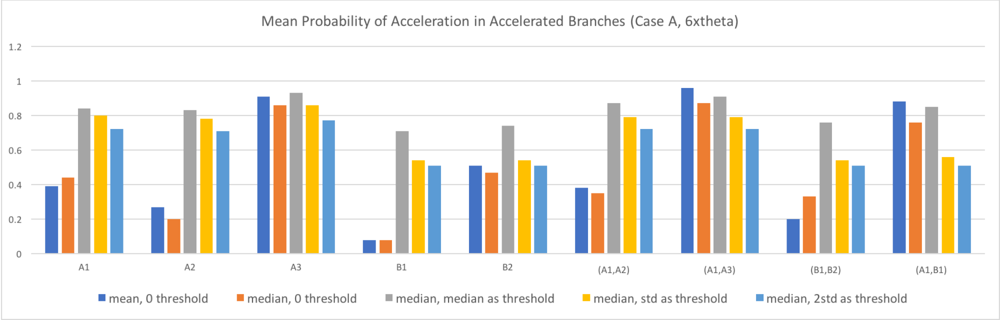

(c) Case A, 6θ

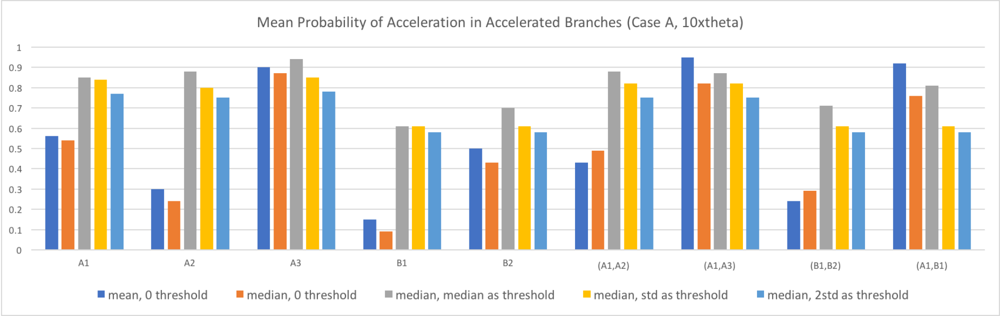

(d) Case A, 10θ

Figure S18: Average probability of acceleration for accelerated branches under Case A, and different θ magnitudes.

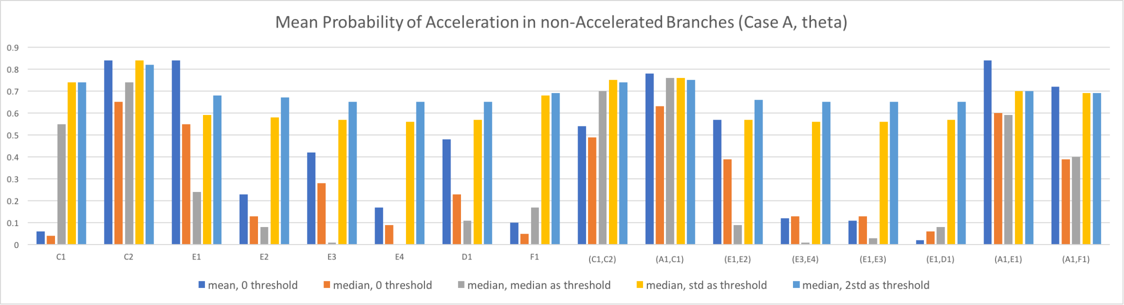

(a) Case A, θ

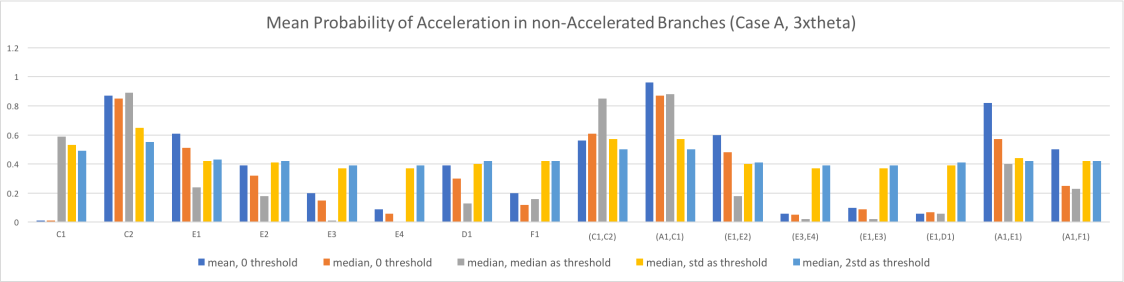

(b) Case A, 3θ

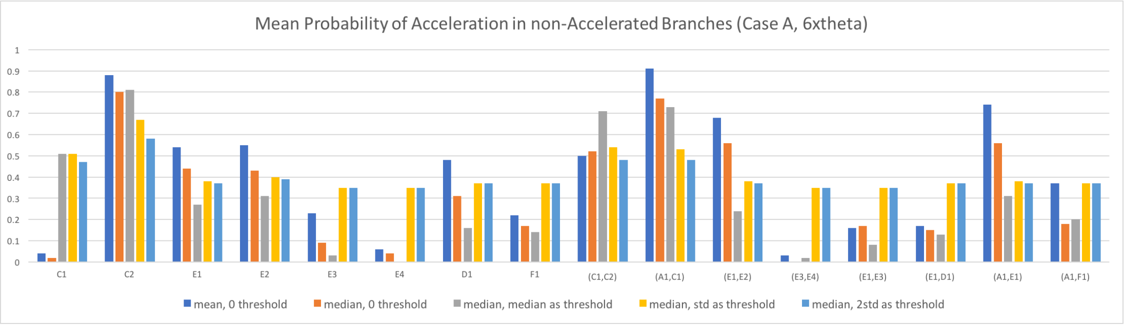

(c) Case A, 6θ

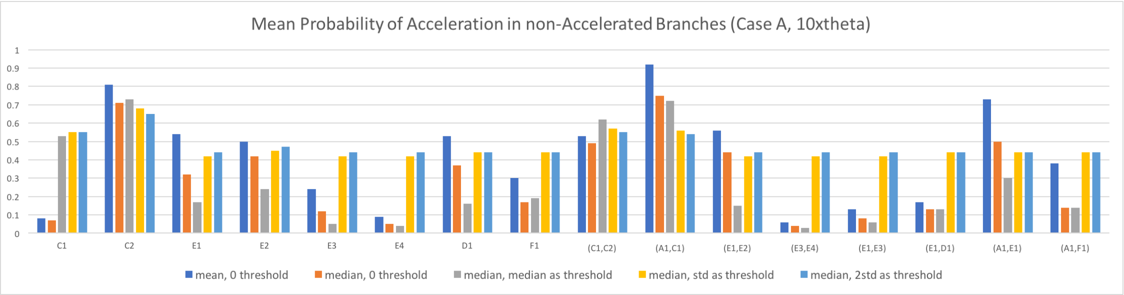

(d) Case A, 10θ

Figure S19: Average probability of acceleration for non-accelerated branches under Case A, and different θ magnitudes.

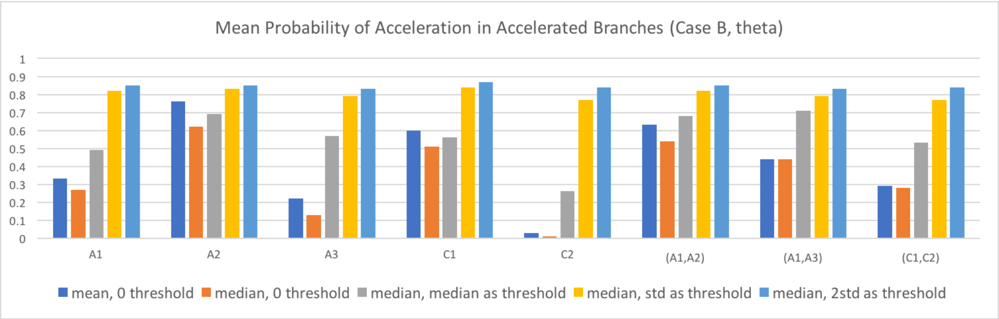

(a) Case B, θ

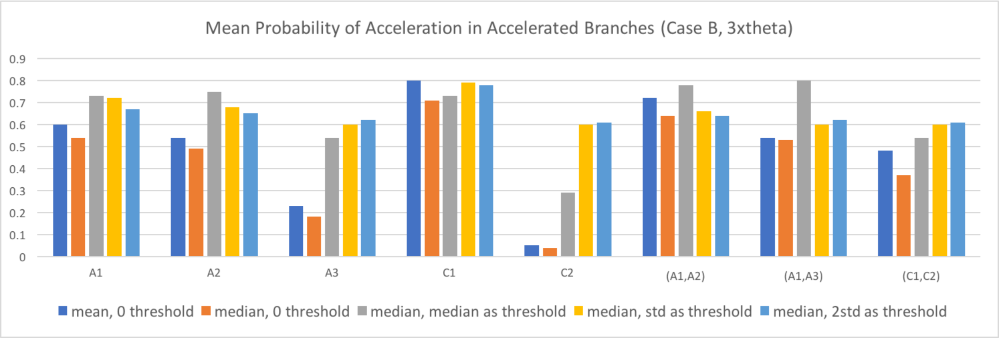

(b) Case B, 3θ

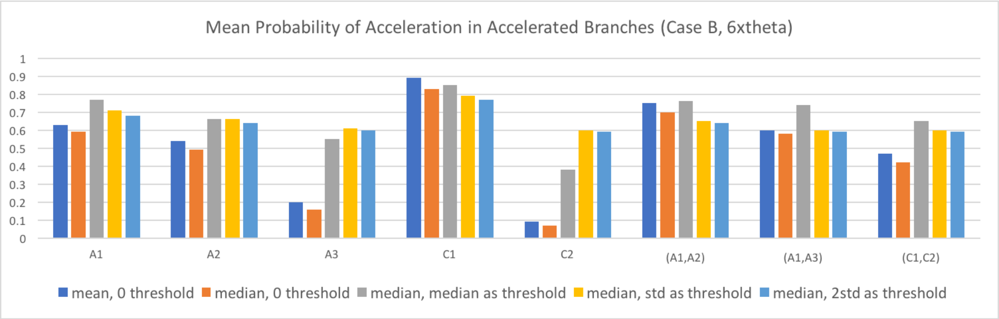

(c) Case B, 6θ

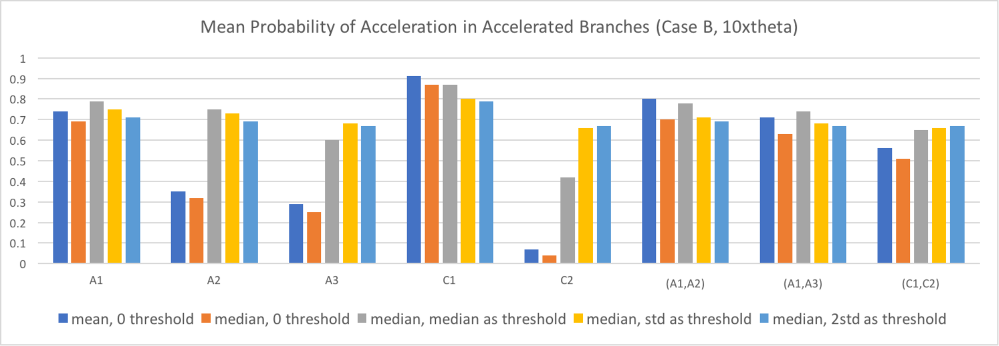

(d) Case B, 10θ

Figure S20: Average probability of acceleration for accelerated branches under Case B, and different θ magnitudes.

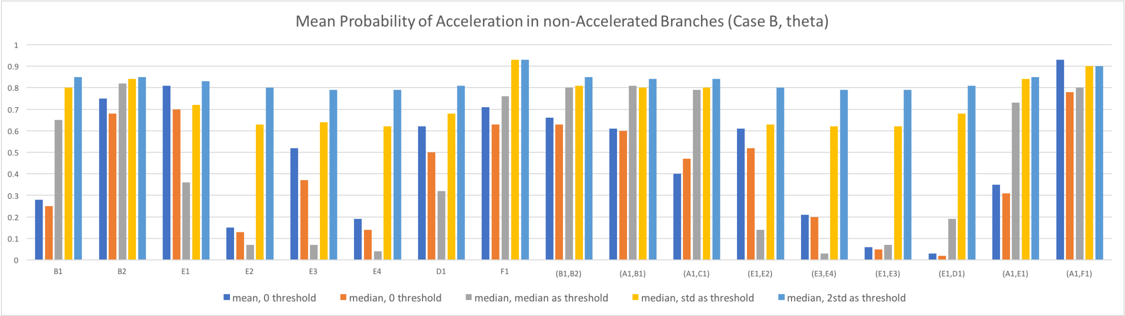

(a) Case B, θ

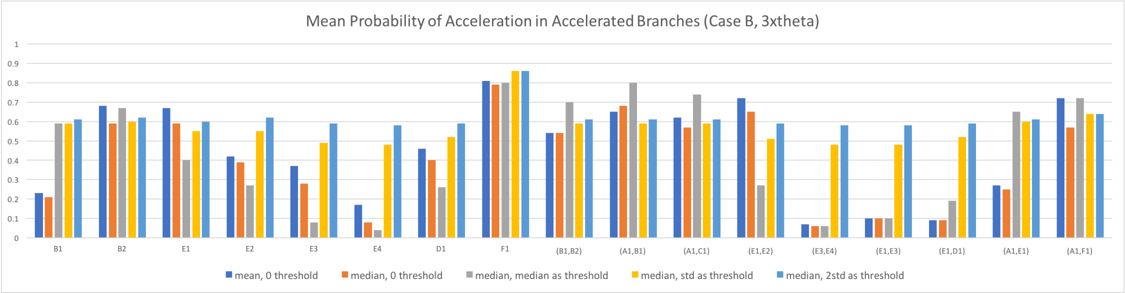

(b) Case B, 3θ

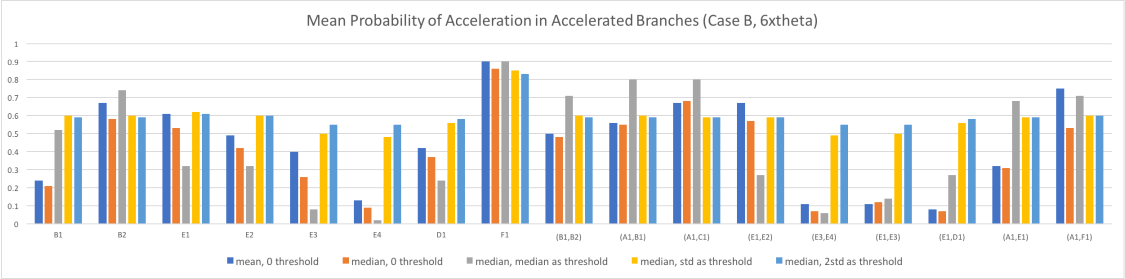

(c) Case B, 6θ

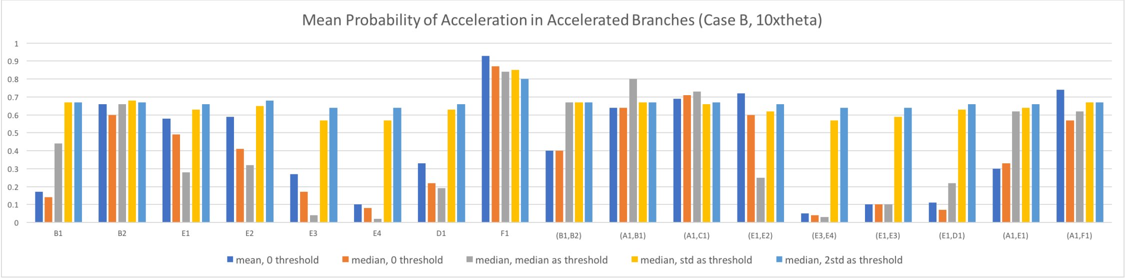

(d) Case B, 10θ

Figure S21: Average probability of acceleration for non-accelerated branches under Case B, and different θ magnitudes.

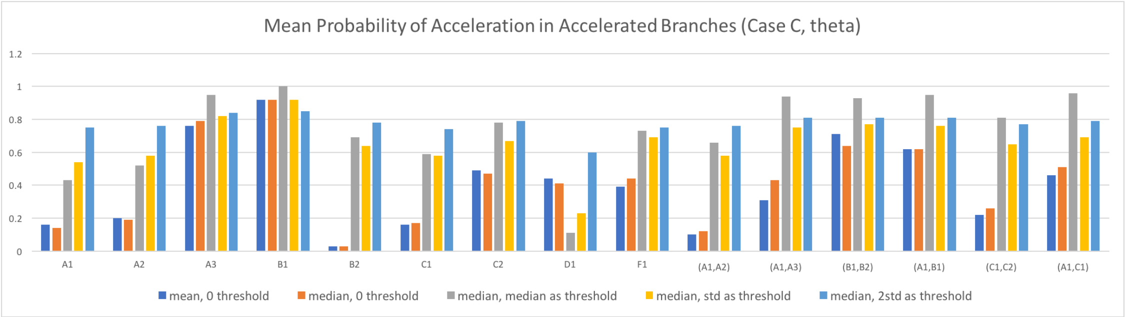

(a) Case C, θ

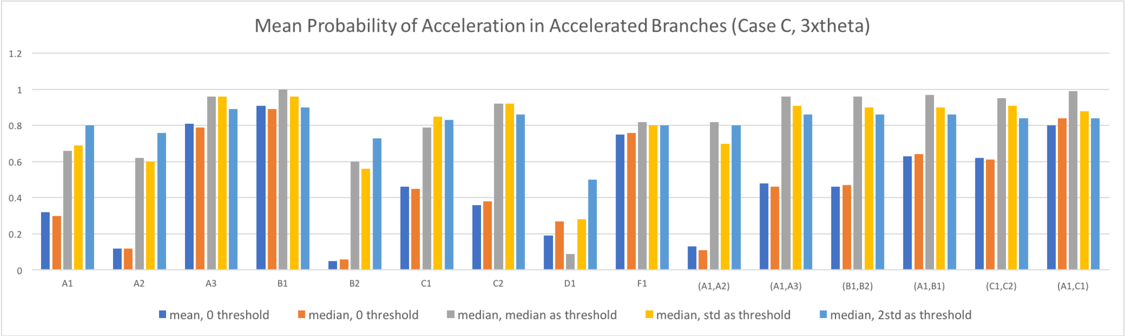

(b) Case C, 3θ

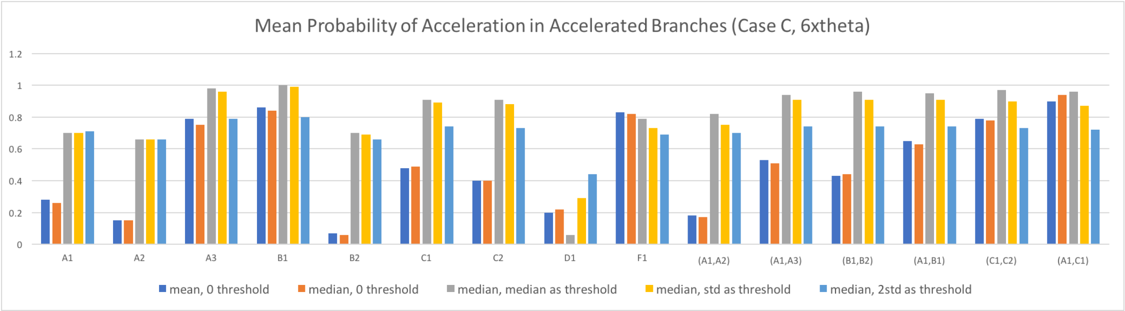

(c) Case C, 6θ

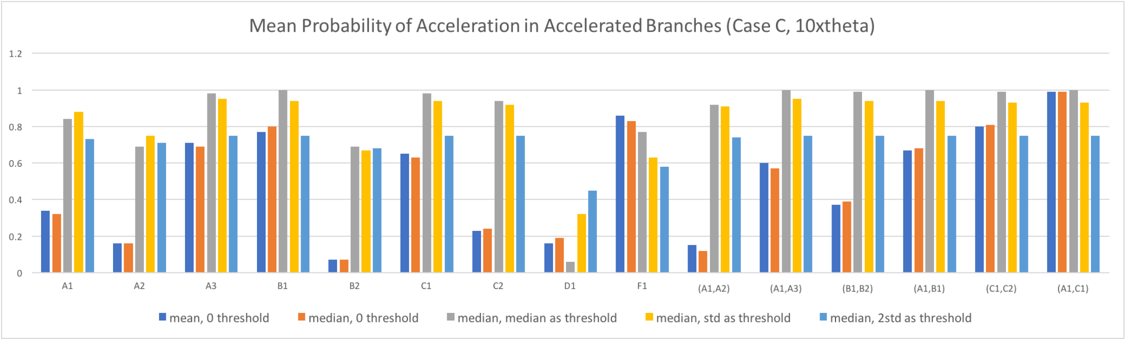

(d) Case C, 10θ

Figure S22: Average probability of acceleration for accelerated branches under Case C, and different θ magnitudes.

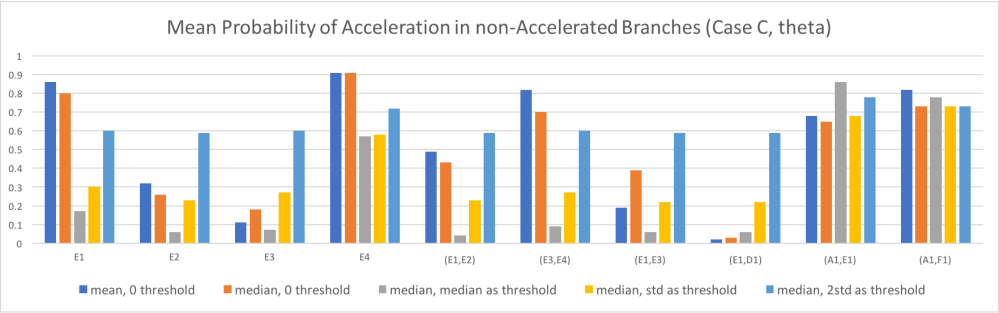

(a) Case C, θ

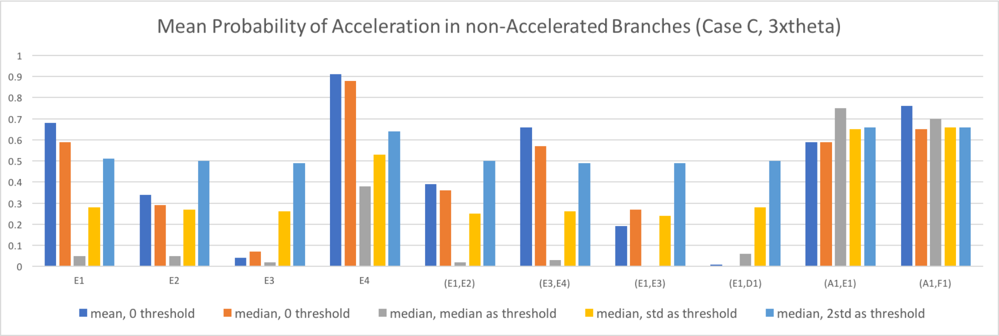

(b) Case C, 3θ

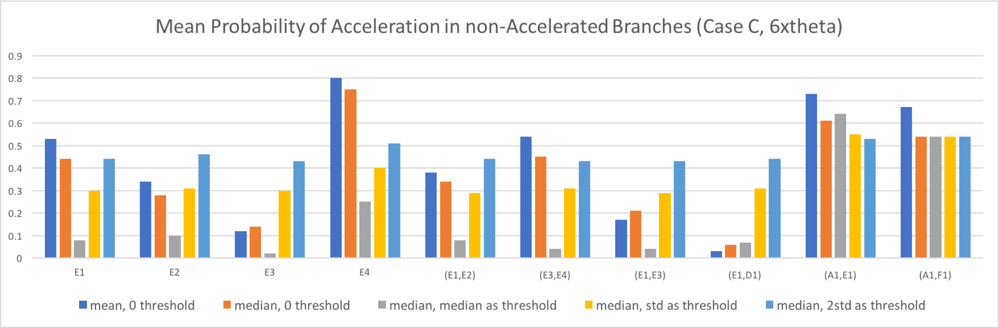

(c) Case C, 6θ

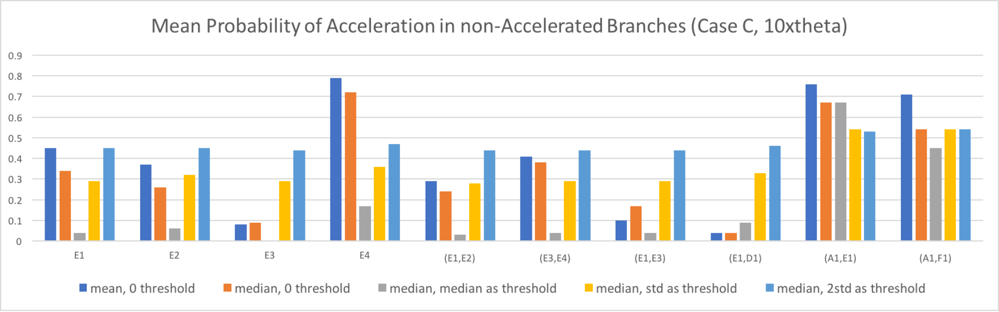

(d) Case C, 10θ

Figure S23: Average probability of acceleration for non-accelerated branches under Case C, and different θ magnitudes.

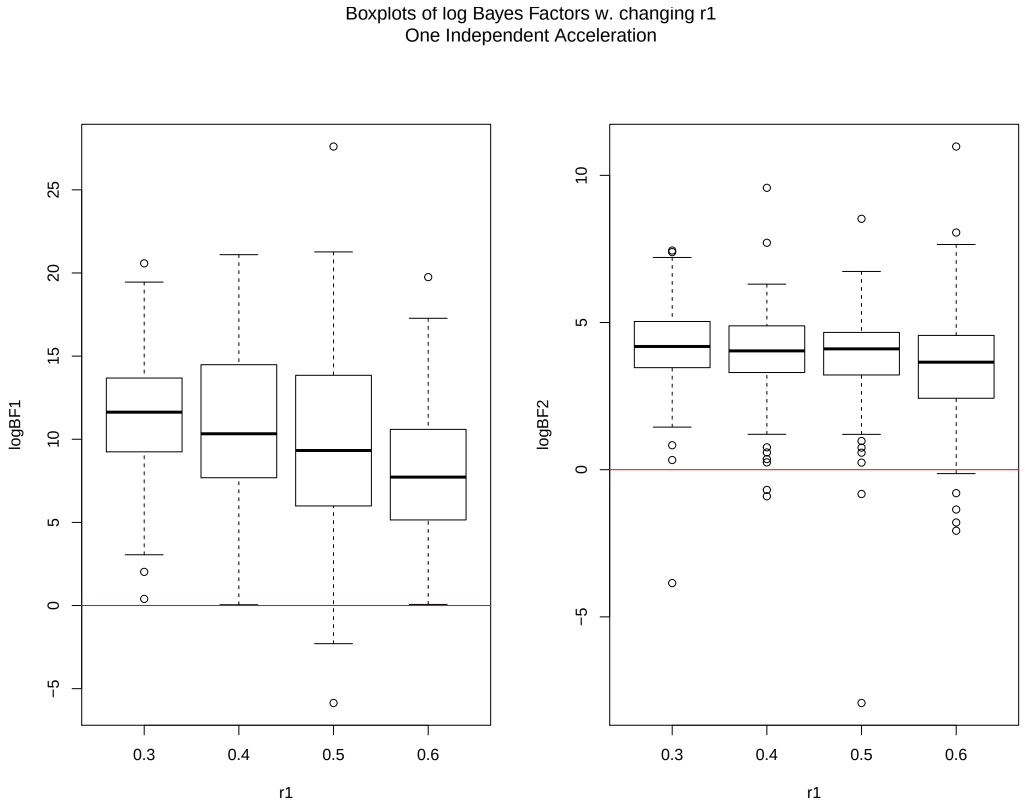

Figure S24: Boxplots of log Bayes Factors as the mean conserved rate increases. Case: one independent acceleration.

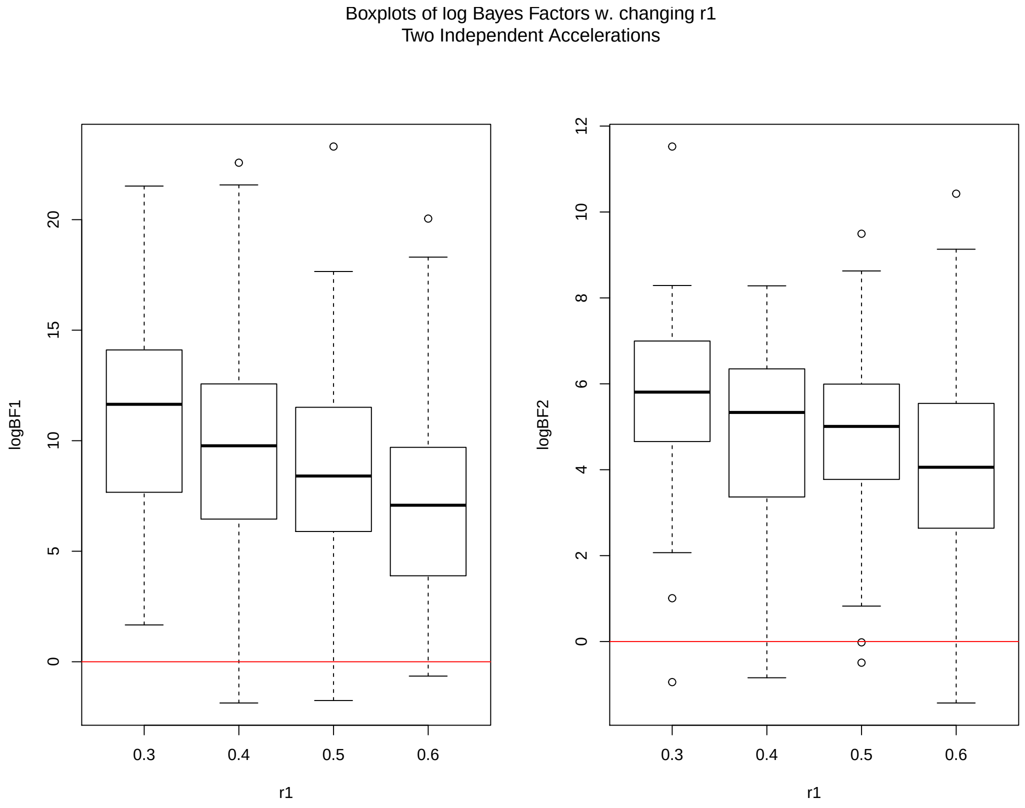

Figure S25: Boxplots of log Bayes Factors as the mean conserved rate increases. Case: two independent accelerations.

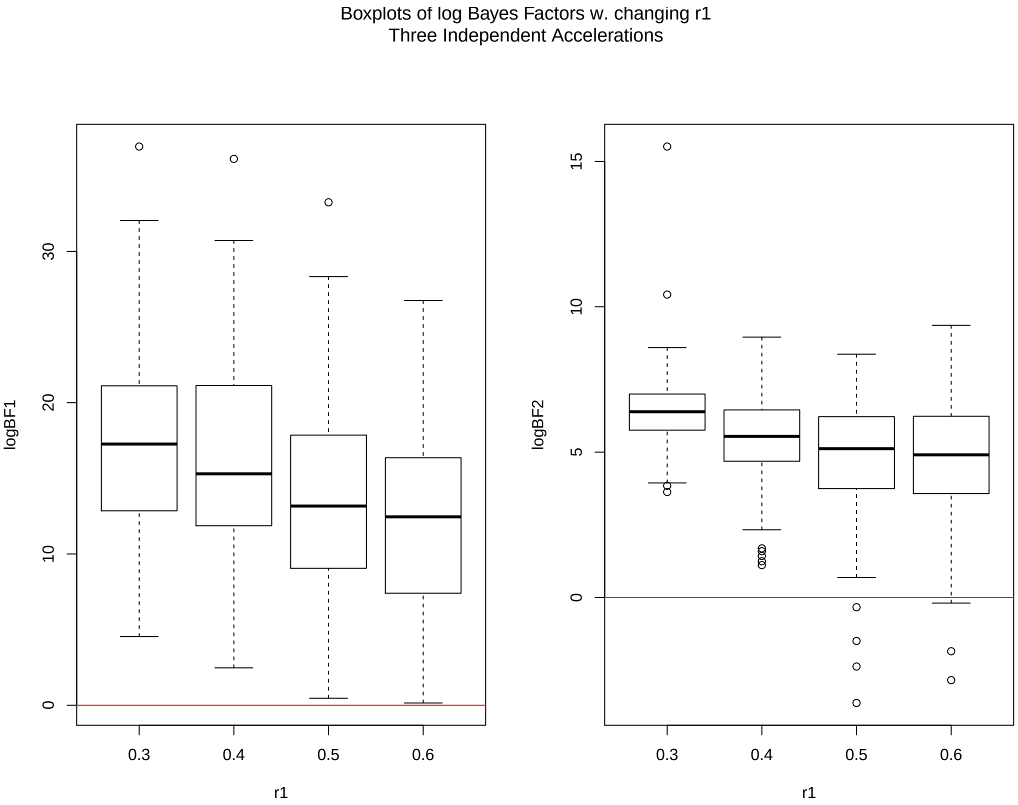

Figure S26: Boxplots of log Bayes Factors as the mean conserved rate increases. Case: three independent accelerations.

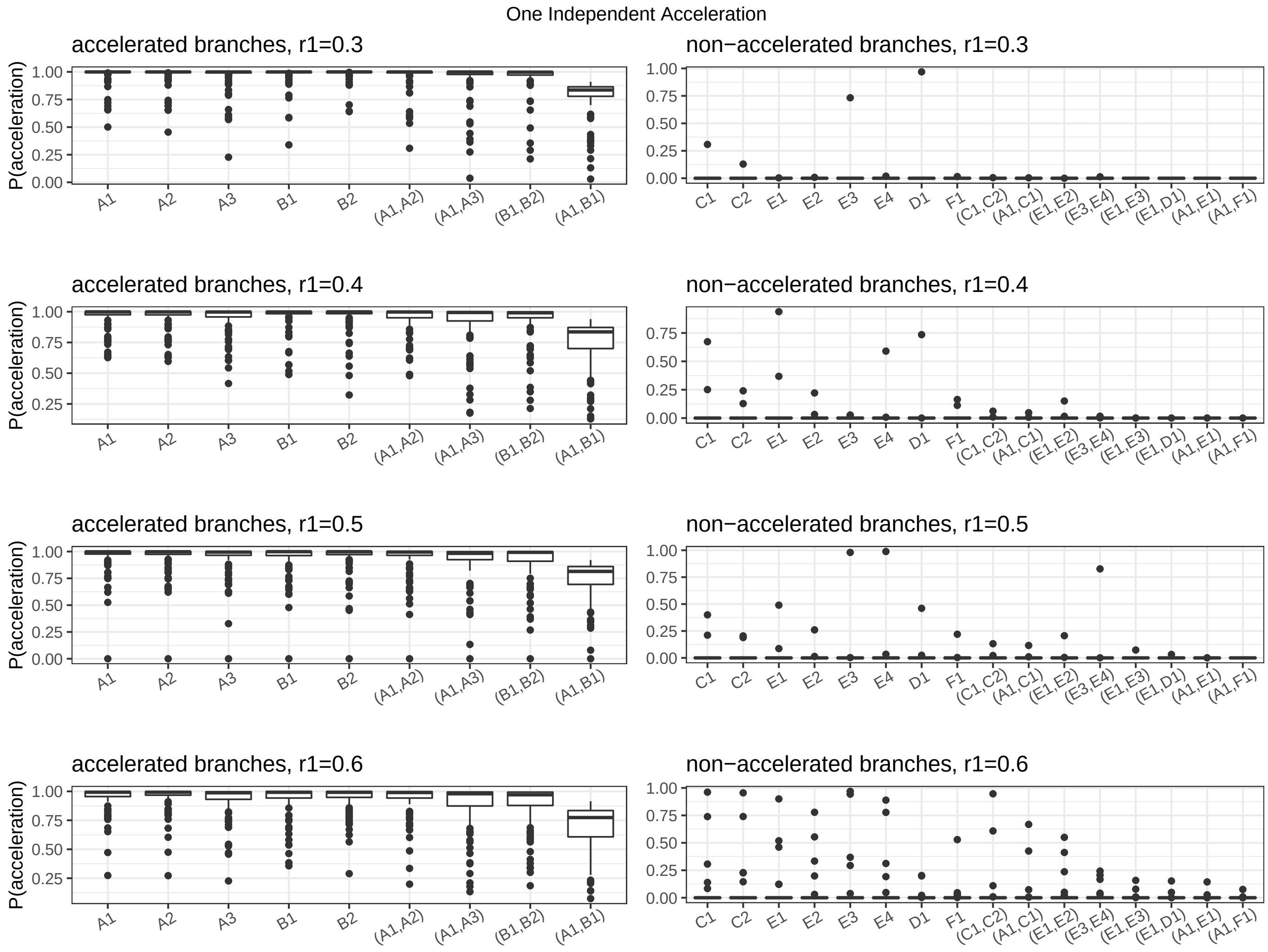

Figure S27: Boxplots of $P(Z = 2 \mid \boldsymbol{Y})$ for all branches on the tree as the mean conserved rate increases. Case: one independent acceleration.

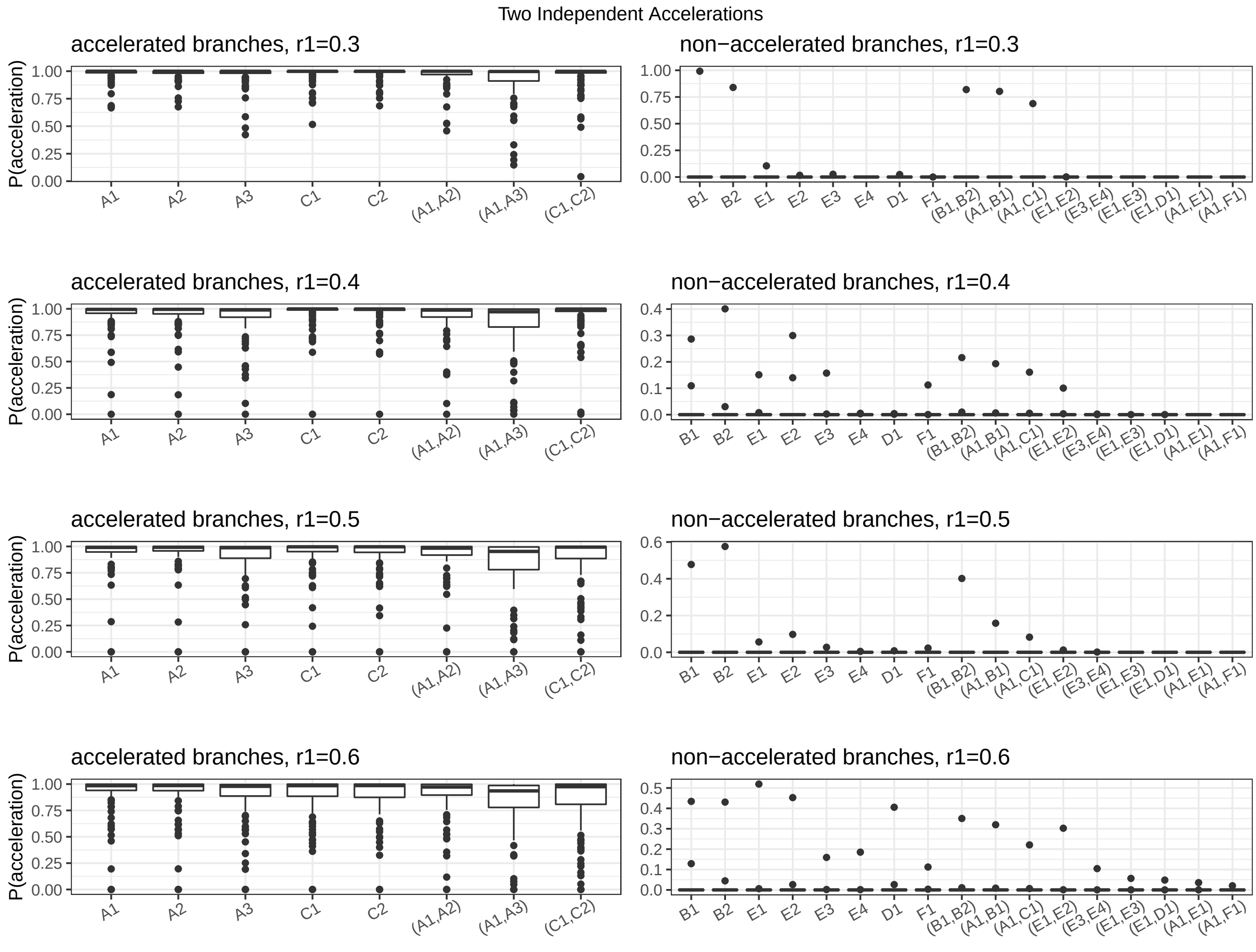

Figure S28: Boxplots of $P(Z = 2 \mid \boldsymbol{Y})$ for all branches on the tree as the mean conserved rate increases. Case: two independent accelerations.

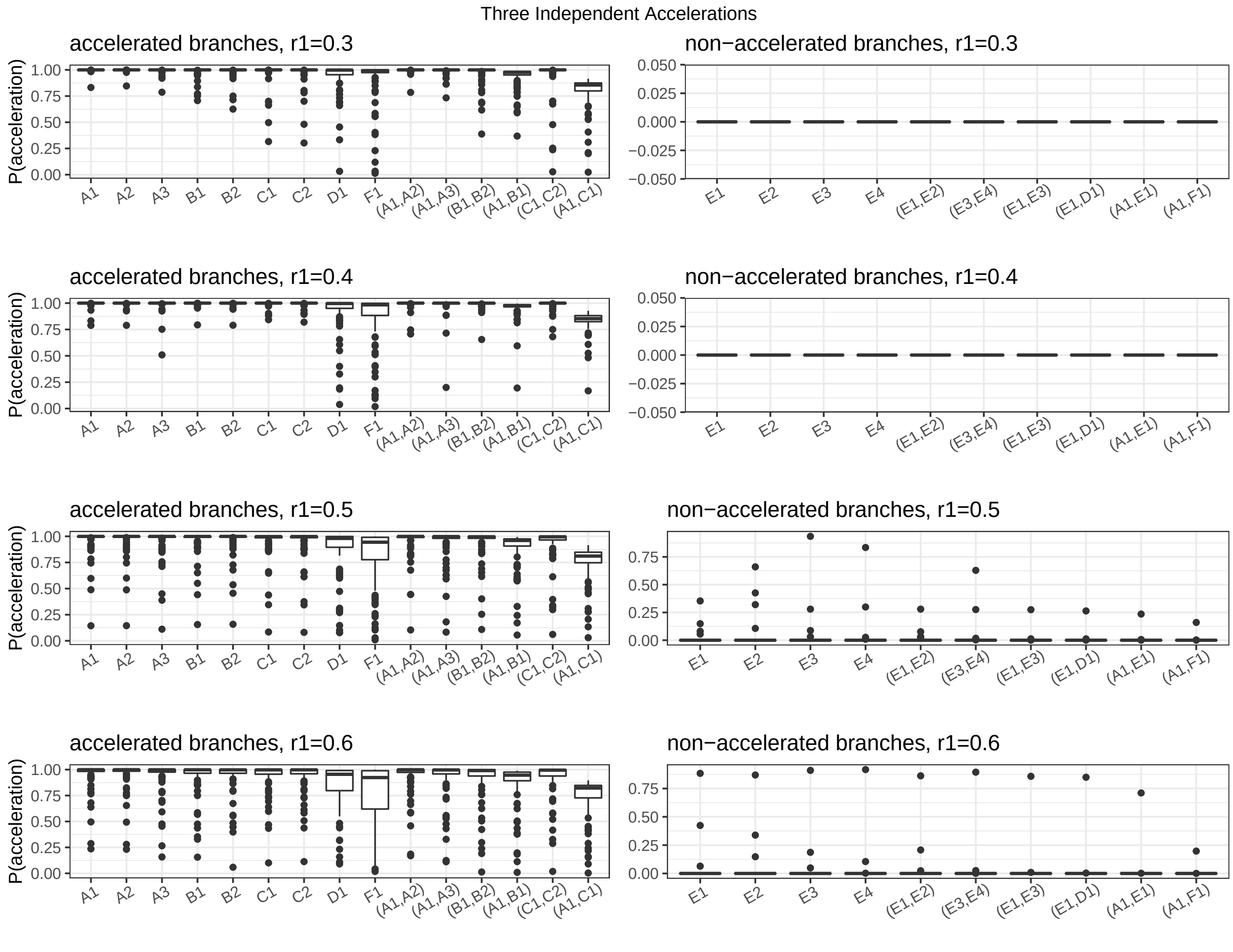

Figure S29: Boxplots of $P(Z = 2 \mid \boldsymbol{Y})$ for all branches on the tree as the mean conserved rate increases. Case: three independent accelerations.

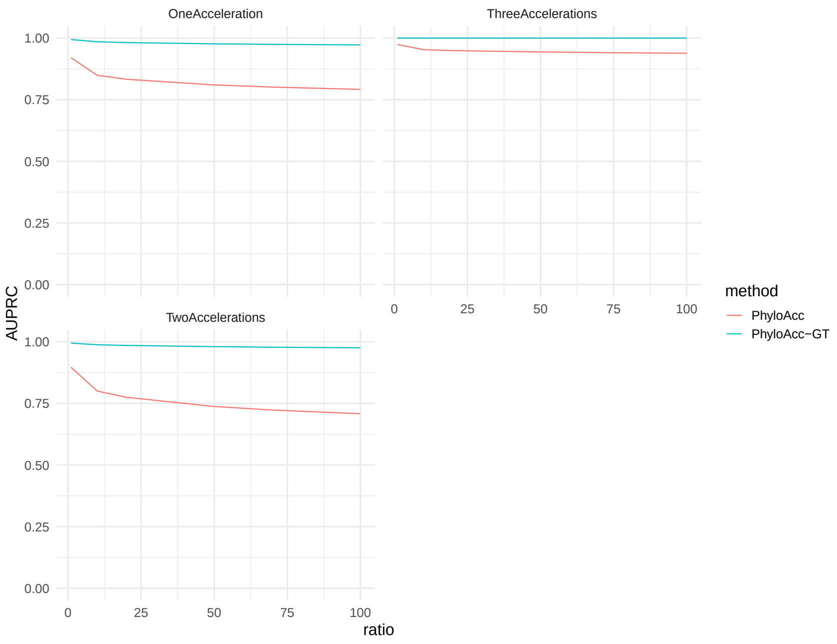

Figure S30: AUPRC curves by PhyloAcc-GT and PhyloAcc when r_1 is centered at 0.3.

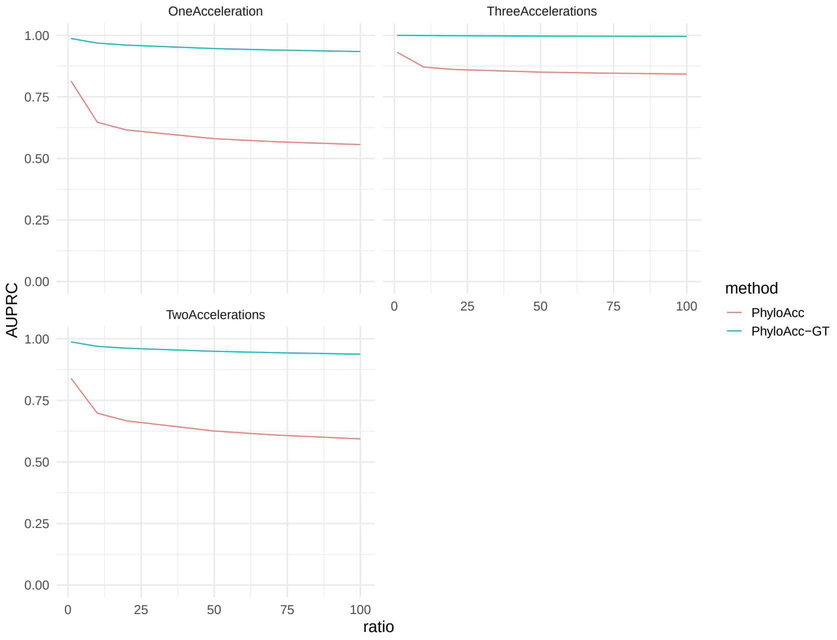

Figure S31: AUPRC curves by PhyloAcc-GT and PhyloAcc when r_1 is centered at 0.4.

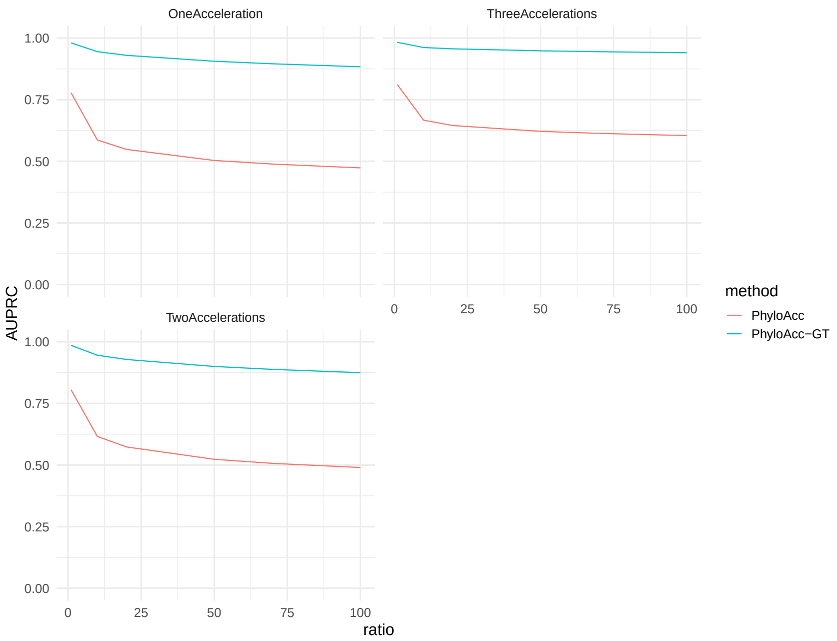

Figure S32: AUPRC curves by PhyloAcc-GT and PhyloAcc when r_1 is centered at 0.5.

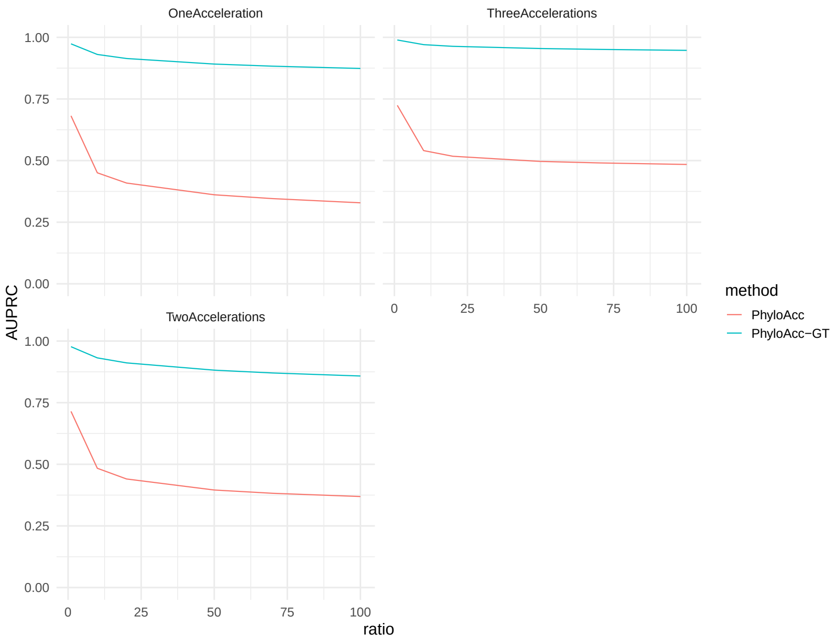

Figure S33: AUPRC curves by PhyloAcc-GT and PhyloAcc when r_1 is centered at 0.6.

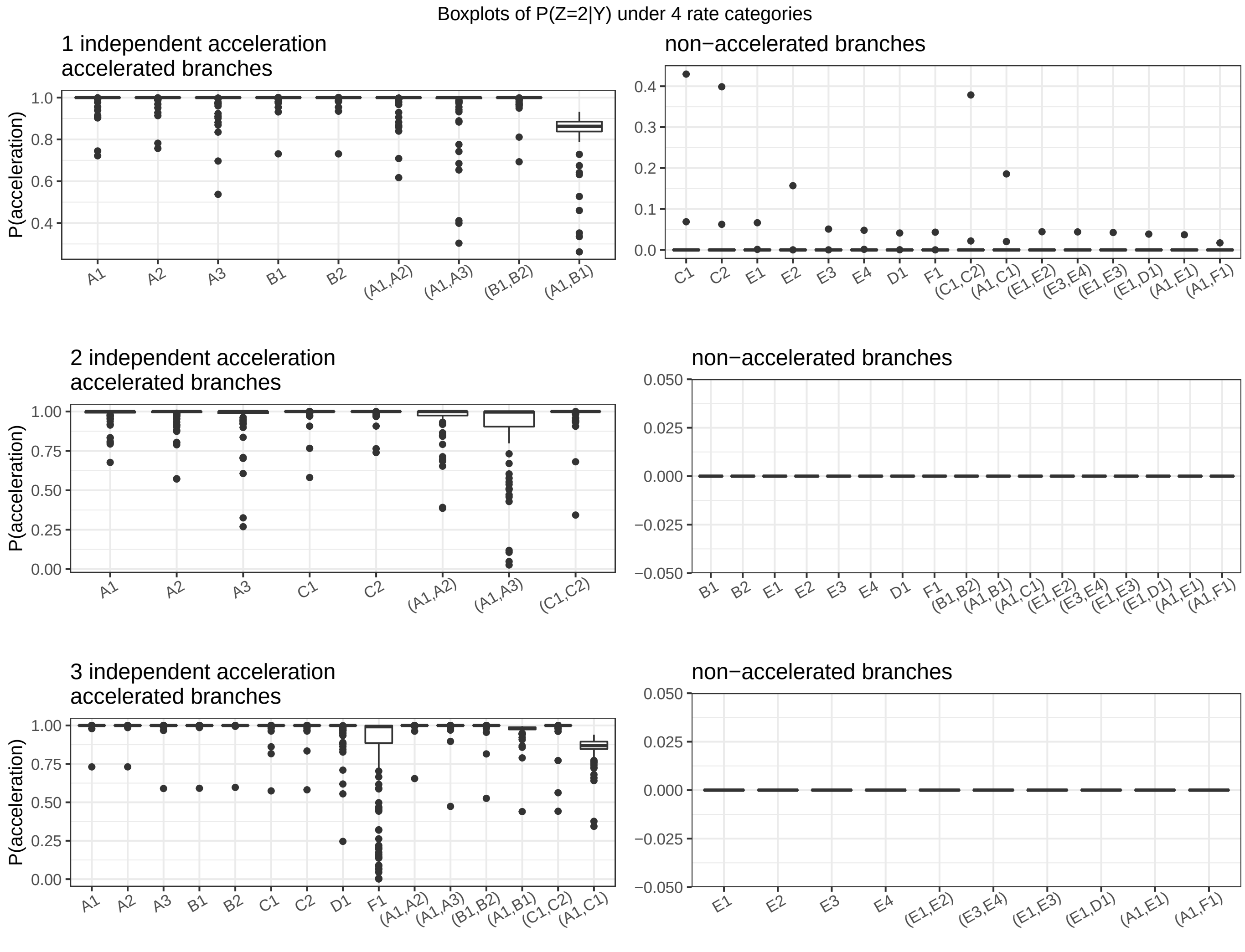

Figure S34: Boxplots of $P(Z = 2 \mid \boldsymbol{Y})$ for all branches on the tree when there are 4 rate categories

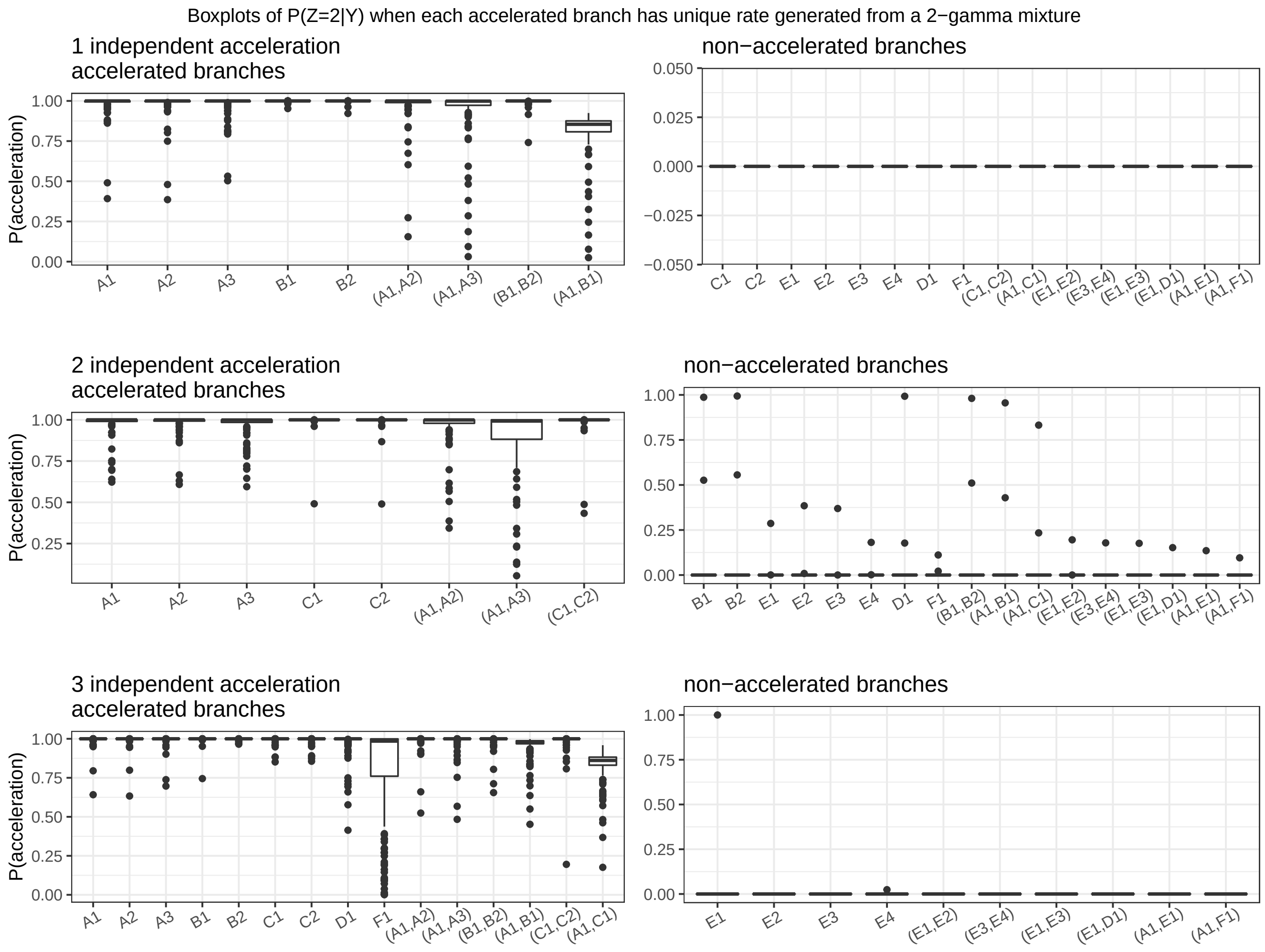

Figure S35: Boxplots of $P(Z = 2 \mid \boldsymbol{Y})$ for all branches on the tree when a locus in each accelerated branch has an unique accelerated rate.

S8 Modeling the substitution stationary distribution improves the estimation of substitution rates

The DNA nucleotide stationary distribution $\boldsymbol{\pi}$ directly affects the transition rate matrix Q. If Q is not correctly specified, it affects the estimation of substitution rates and potentially conservation states. In this section, we investigate the effect of $\boldsymbol{\pi}$ on model performance.

We use the same phylogeny as in Figure 2A, and simulate DNA sequence from the null model, i.e., no branch is accelerated. We simulate 100 loci, having 200 base pairs each. Each locus has its own $\boldsymbol{\pi}$. For 50 elememnts, we simulated $2\pi_A \sim Beta(5,5)$, and for the rest 50 loci, we simulated $2\pi_A \sim Beta(10,10)$. We have $\boldsymbol{\pi} = (\pi_A, \pi_C, \pi_G, \pi_T) = (\pi_A, \frac{1}{2} - \pi_A, \frac{1}{2} - \pi_A, \pi_A)$. Conserved rates are simulated from $gamma(5, 0.04)$. We run our algorithm for each locus at 3 treatments of $\boldsymbol{\pi}$:

1. Treatment 1: fixing $\boldsymbol{\pi}$ at truth;
2. Treatment 2: fixing $\boldsymbol{\pi}$ at the value estimated from neutral sites, denoted by $\boldsymbol{\pi}^n = (\pi_A^n, \pi_C^n, \pi_G^n, \pi_T^n)$;
3. Treatment 3: modeling the variation in $\boldsymbol{\pi}$ according to the Bayesian model in Section Methods in the main text.

When we designate the target group, for half of the loci we use sequences simulated with two independent accelerations (Figure 2C) and for the other half, we use sequences simulated with a single acceleration (Figure 2B).

Model selection accuracy is recorded in Table S1. All three treatments are highly accurate in detecting no acceleration patterns along the phylogeny. Next, we check the posterior distributions of the conserved rate. We use the posterior distribution of r_1 estimated estimated under Treatment 1 as a reference distribution. Figures S36 and S37 show that the posterior distributions are very close to the reference distribution whether we model $\boldsymbol{\pi}$ or not. Modeling $\boldsymbol{\pi}$ introduces slightly more variations in the upper tail probability of r_1 as shown in the bottom left plot in Figure S37.

Distribution to Simulate $\boldsymbol{\pi}$	Model	Model Selection Accuracy
$Beta(10, 10)$	1. Fix $\boldsymbol{\pi}$ at truth	100%
	2. Use $\boldsymbol{\pi}^n$	100%
	3. Estimate $\boldsymbol{\pi}$ from data	100%
$Beta(5, 5)$	1. Fix $\boldsymbol{\pi}$ at truth	96%
	2. Use $\boldsymbol{\pi}^n$	98%
	3. Estimate $\boldsymbol{\pi}$ from data	98%

Table S1: Accuracy in model selection and r_c estimation under the three models. Model selection accuracy is the percentage of cases the null model M_0 is selected based on Bayes Factor cutoff at 1.

The two Beta distributions we use to simulate $\pi_A + \pi_T$ are centered at 0.5. Hence, the $\boldsymbol{\pi}$'s generated are likely to have balanced weights on all four nucleotides, and do not differ too

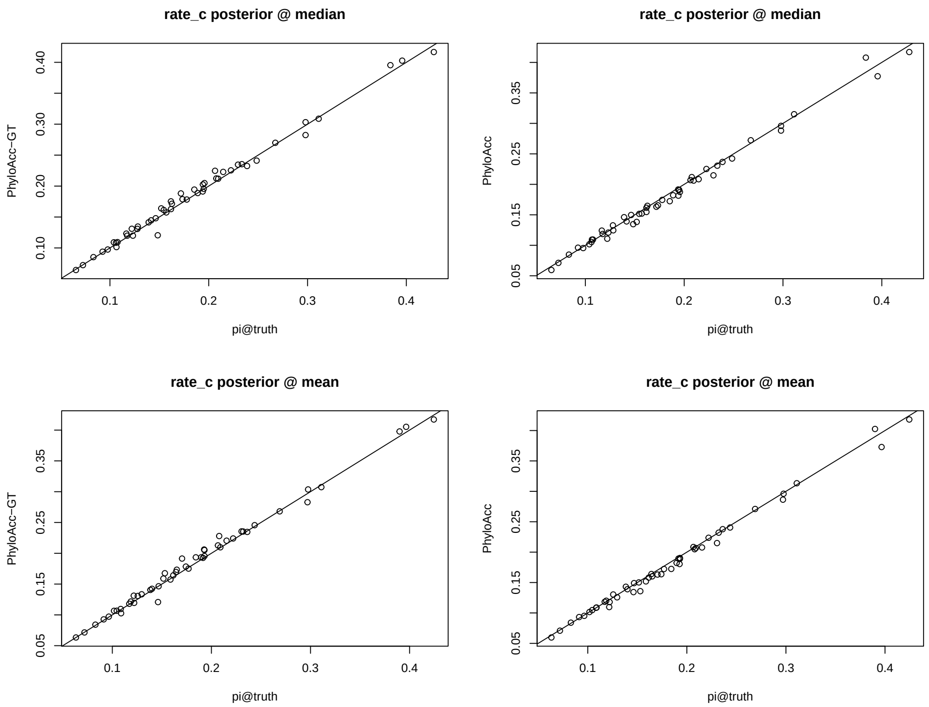

Figure S36: scatter plots comparing point estimates of r_c using the three models.

much from $\boldsymbol{\pi}^n$. The mean absolute difference between the simulated π_A's and π_A^n is 0.066. Since the differences are small, the rate matrix Q computed from $\boldsymbol{\pi}^n$ also does not differ much from the true Q's used to simulate the data. Results from the above study suggest that if $\boldsymbol{\pi}$ is misspecified by a small amount, it will not have an impact on inferring the posterior distributions of substitution rate and conservation state.

Next, we investigate whether modeling $\boldsymbol{\pi}$ will improve model performance from PhyloAcc when the input $\boldsymbol{\pi}$ value is far from the truth. We simulate 100 $2\pi_A$s from two distributions: $Beta(3,1)$ and $Beta(1,4)$. $Beta(3,1)$ tends to produce $\boldsymbol{\pi}$s that put high weights on adenine and thymine, while $Beta(1,4)$ the opposite. We filter out values of π_A that are either greater than 0.4 or less than 0.1, which gives us 56 cases. Using these unbalanced $\boldsymbol{\pi}$'s we generate

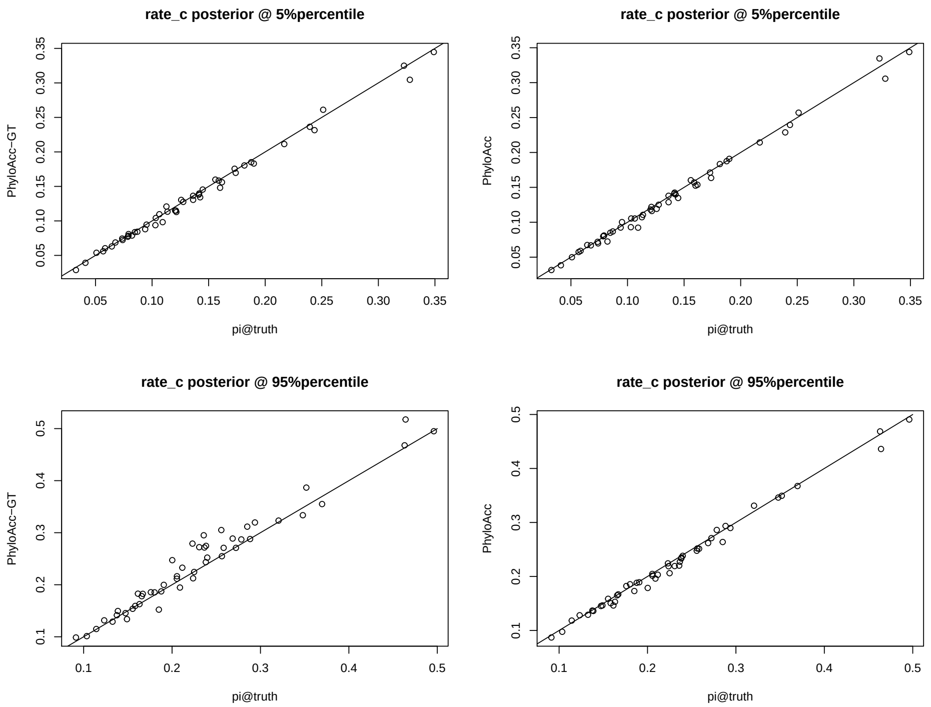

Figure S37: Scatter plots comparing tail behavior of the posterior distribution of r_c using the three models.

data and test our models. When applying PhyloAcc, we input a $\boldsymbol{\pi}$ that put most weight on cytosine and guanine if the true $\boldsymbol{\pi}$ is highly concentrated on adenine and thymine, or the other way around.

In this extreme case, PhyloAcc still achieves 100% model selection accuracy, and the accuracy is 94.4% for PhyloAcc-GT. Both are accurate in identifying $\mathcal{M}_0$ as the correct model. However, PhyloAcc underestimates the substitution rate as shown in Figure S38, while PhyloAcc-GT can still accurately inference the substitution rate. If the data set is generated with a large π_A value, most of the base-pair positions will show A or T across extant species. However, since the input π_A is very small when running PhyloAcc, the sequences

are more likely to transit from A and T to C and G. To observe the high frequency of A and T and high similarities among sequences, PhyloAcc has to infer that DNA substitution will be highly conserved.

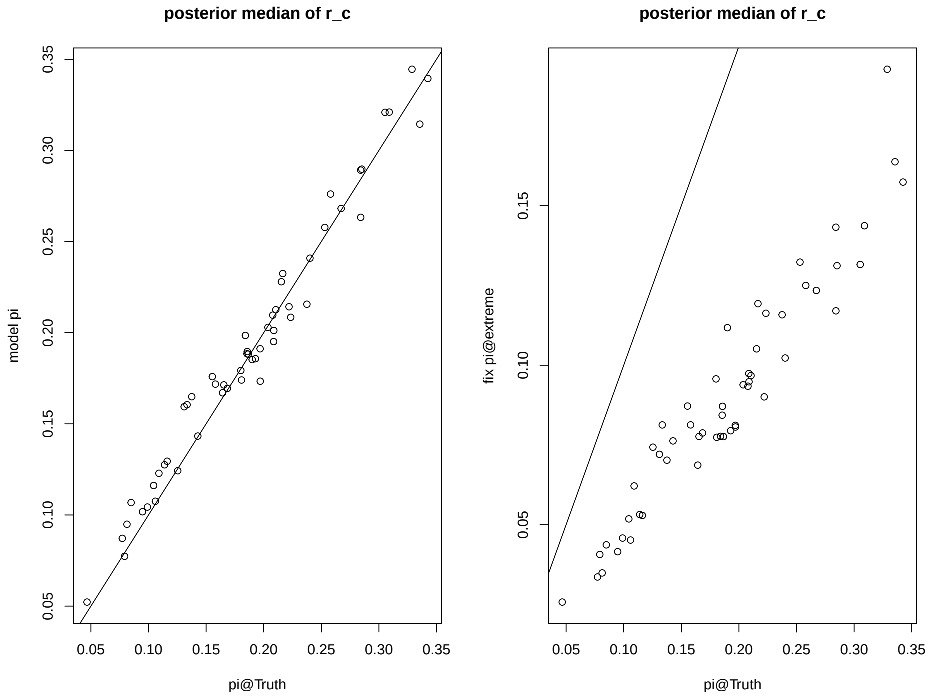

Figure S38: Comparing posterior mediums of the substitution rate from the three models

S9 Some Analyses of Posterior Gene trees and Patterns of Acceleration for loci of Interest in the Avian Dataset

In this section, we analyze posterior gene trees for locus mCE1358939 and mCE1419808 that favor model $\mathcal{M}_0$ under PhyloAcc-GT. For mCE1358939, with PhyloAcc, Southern cassowary, Little spotted kiwi and Great spotted kiwi are estimated to be in the accelerated state with posterior probability of acceleration being 0.75, 0.85 and 0.56 respectively. It is likely that the acceleration in the two kiwis occurred in their parent species ($P(Z = 2 \mid \boldsymbol{Y}) = 0.52$). Under PhyloAcc-GT, the four species are still the top four species that are likely to have experienced rate accelerations under $\mathcal{M}_1$, but only Southern cassowary and Little spotted kiwi have posterior probabilities of acceleration exceeding 0.5. Under $\mathcal{M}_1$, the gene tree at the posterior mode places the Rhea clade directly under Ostrich, and (Southern Cassowary, Emu) becomes the sibling branch of (Moa, Tinamous). The same tree topology is also the most likely topology under model $\mathcal{M}_0$. However, there are increases in the estimated gene tree branch lengths for the four branches under $\mathcal{M}_0$, and most non-accelerated branches are shorter under $\mathcal{M}_0$ than under $\mathcal{M}_1$.

Using PhyloAcc, mCE1419808 is estimated to have experienced strong rate accelerations in Ostrich ($P(Z = 2 \mid \boldsymbol{Y}) = 1$), followed by Great spotted kiwi and Little spotted kiwi ($P(Z = 2 \mid \boldsymbol{Y}) = 0.56$ for both). Using PhyloAcc-GT, the gene tree topology among ratites at posterior mode under $\mathcal{M}_0$ and $\mathcal{M}_1$ are both the same as the species tree topology.

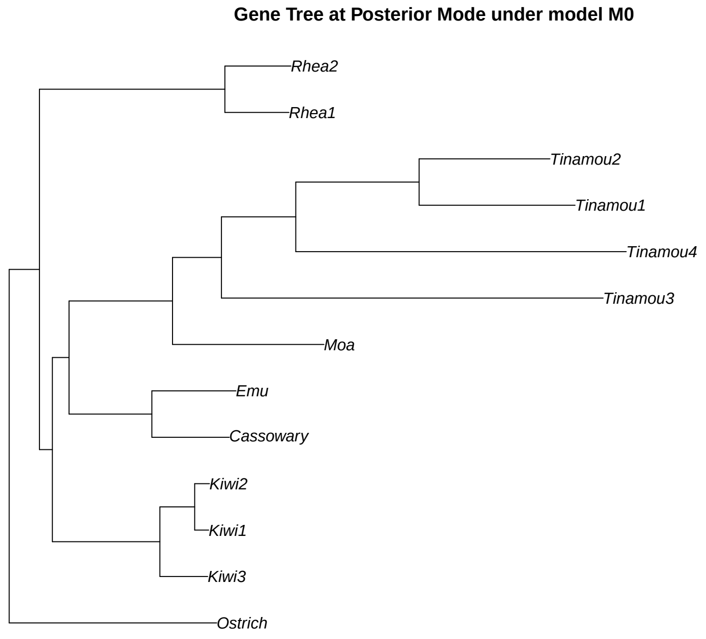

Figure S39: The gene tree topology at posterior mode under model $\mathcal{M}_0$ for locus mCE1358939.

However, the gene tree branch lengths differ from those of the species tree, resulting in different patterns of acceleration. In the posterior mode of the gene tree under model $\mathcal{M}_1$, the estimated branch length for Ostrich is 8% longer than the corresponding length on the species tree. As a result, the estimated posterior probability of acceleration in Ostrich reduces to 0.5 under PhyloAcc-GT. On the other hand, posterior probabilities of acceleration are greater in (Great spotted kiwi, Little spotted kiwi), (Greater rhea, Lesser rhea), and (Cassowary, Emu) under PhyloAcc-GT compared to the estimated probabilities using PhyloAcc, because gene tree branch lengths are estimated to be shorter than species tree branch lengths. Although

some branches are estimated to have rate accelerated under $\mathcal{M}_1$, and the ratite tree topology at the posterior mode are the same under $\mathcal{M}_0$ and $\mathcal{M}_1$, after marginalizing over the gene tree, the data supports model $\mathcal{M}_0$ the most.

S10 Additional Figures from Simulation Studies

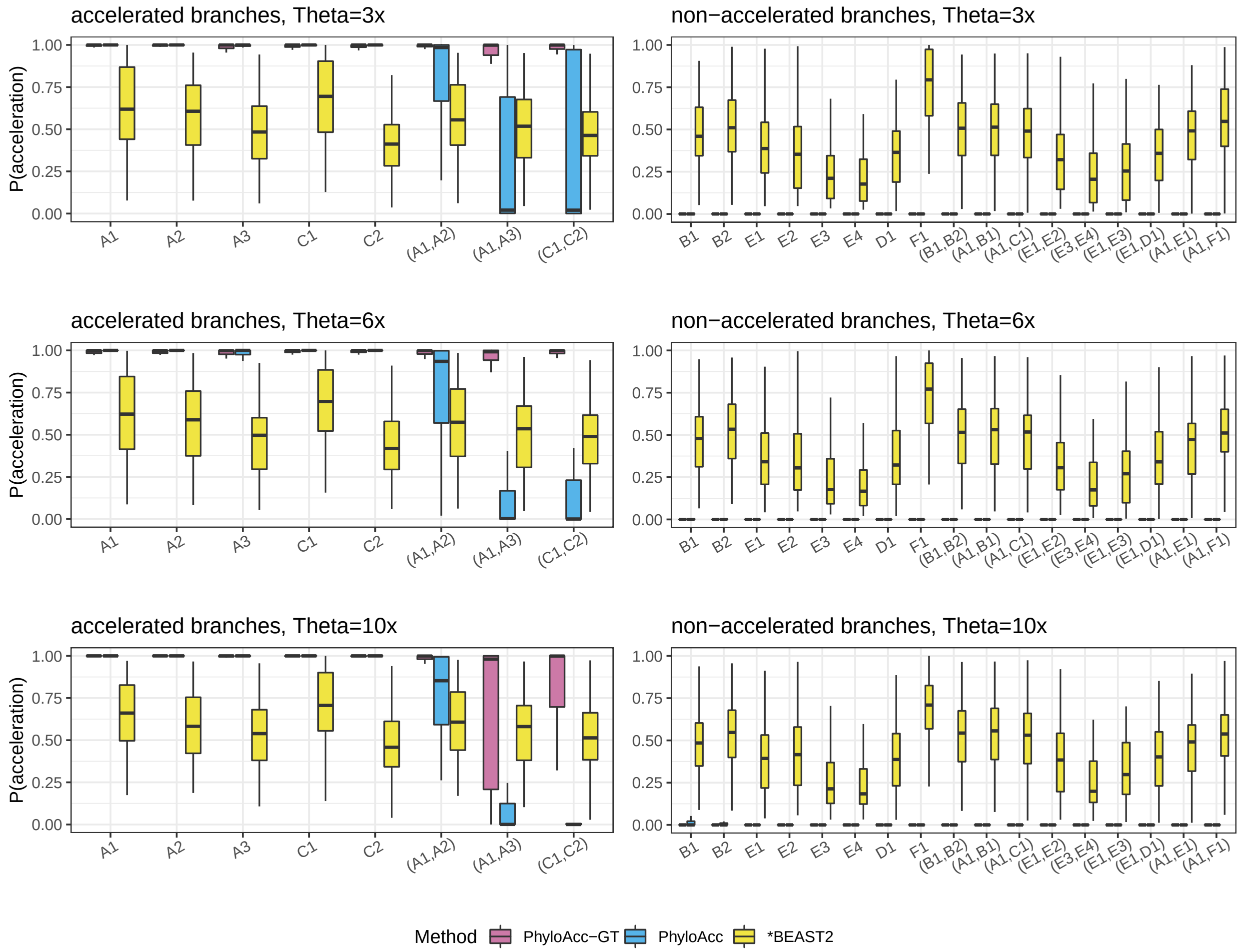

Figure S40: comparing $P(Z = 2 \mid \boldsymbol{Y})$ using PhyloAcc-GT, PhyloAcc and *BEAST2 under the two independent accelerations case (Figure 2C) as $\boldsymbol{\Theta}$ increases. Left plots correspond to truly accelerated branches, whereas plots on the right correspond to non-accelerated branches. We multiply all θ values by 3, 6 or 10, shown in top, middle and bottom rows respectively.

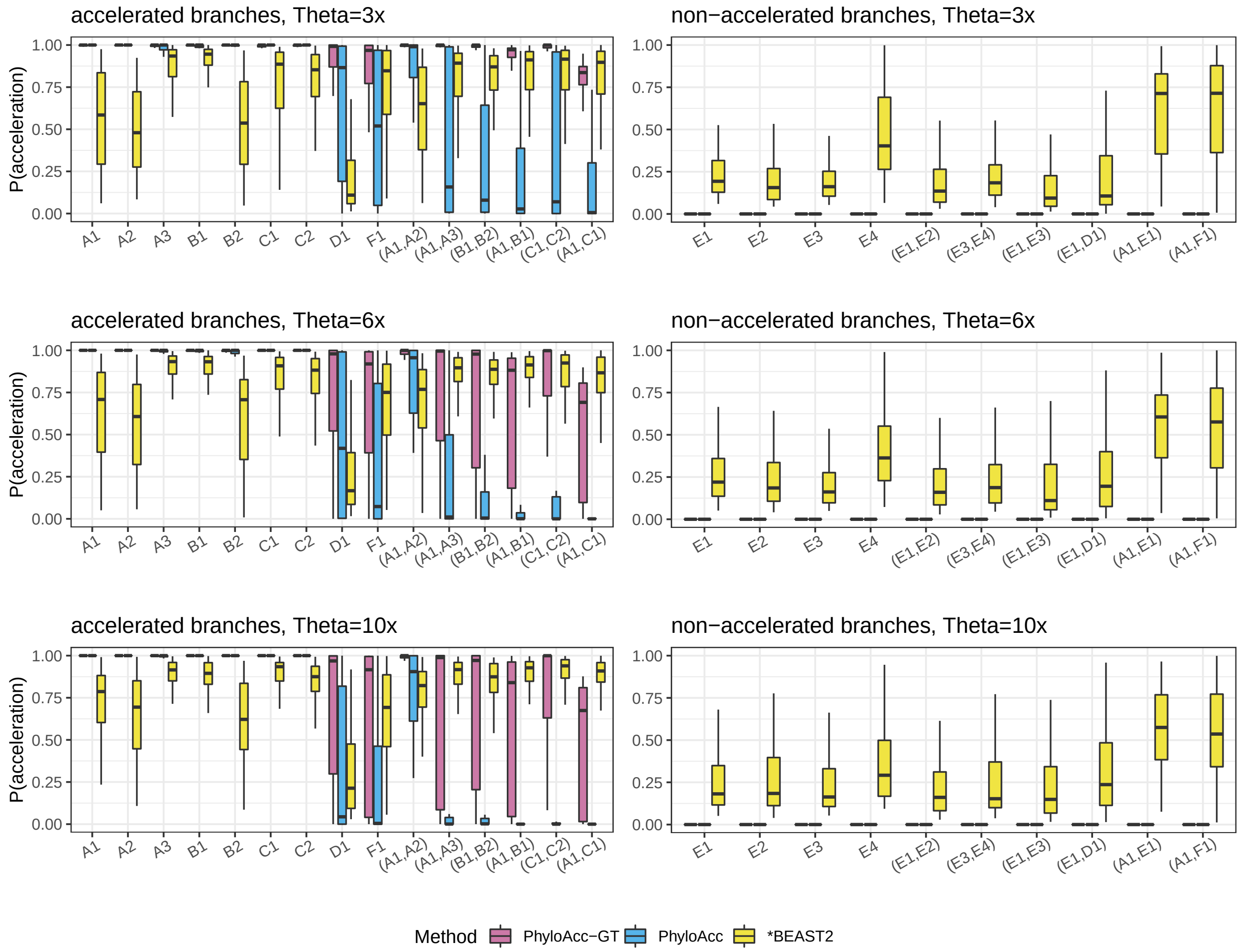

Figure S41: comparing $P(Z = 2 \mid \boldsymbol{Y})$ using PhyloAcc-GT, PhyloAcc and *BEAST2 under the three independent accelerations case (Figure 2D) as $\boldsymbol{\Theta}$ increases. Left plots correspond to truly accelerated branches, whereas plots on the right correspond to non-accelerated branches. We multiply all θ values by 3, 6 or 10, shown in top, middle and bottom rows respectively.

References

Blanchette, M., Kent, W. J., Riemer, C., Elnitski, L., Smit, A. F., Roskin, K. M., Baertsch, R., Rosenbloom, K., Clawson, H., Green, E. D., et al. (2004). Aligning multiple genomic sequences with the threaded blockset aligner. *Genome research*, 14(4):708–715.

Hu, Z., Sackton, T. B., Edwards, S. V., and Liu, J. S. (2019). Bayesian detection of convergent rate changes of conserved noncoding elements on phylogenetic trees. *Molecular biology and evolution*, 36(5):1086–1100.

Liu, L. and Yu, L. (2010). Phybase: an r package for species tree analysis. *Bioinformatics*, 26(7):962–963.

Rannala, B. and Yang, Z. (2003). Bayes estimation of species divergence times and ancestral population sizes using DNA sequences from multiple loci. *Genetics*, 164(4):1645–1656.

Sackton, T. B., Grayson, P., Cloutier, A., Hu, Z., Liu, J. S., Wheeler, N. E., Gardner, P. P., Clarke, J. A., Baker, A. J., Clamp, M., et al. (2019). Convergent regulatory evolution and loss of flight in paleognathous birds. *Science*, 364(6435):74–78.
